# Free Papers

**DOI:** 10.1155/1993/32649

**Published:** 1993


FP001

### SURGICAL TREATMENT OF PANCREATIC NECROSIS

J E Monteiro da Cunha, M C C Machado,
S Penteado, T Bacchella and J Jukemura

São Paulo University Medical School, Department
of Gastroenterology, Surgical Division,
São Paulo, Brazil

Despite progress achieved in the medical treatment of patients with acute pancreatic necrosis (APN) and the role of surgery is no longer in dispute, controversy still exists regarding the ideal time of surgery. We have reviewed our experience at SPUMS between 1980 and 1991 with 68 patients undergoing surgery for APN. Patients with pancreatic abscess were not included. The most common etiologies were biliary(n=25), alcohol (n=16) and postoperative pancreatitis (n=9). Proportion of males to females was 3.8:1 and the average age was 46 years. The APACHE II score was calculated in all patients and it was higher than 9 in 48% (n=33).

Thirty-three patients were operated before 14 days of illness onset and 35 were operated after the second week onset at the appearance of evidence of infected necrosis. The APACHE II scores for both groups were not different. The surgical procedure consisted of necrosectomy plus extended drainage. Re-operation for persistent sepsis occurred in 87.8% (n=29) in the early surgery group patients and in 34.3% (n=12) in the delayed treated patients. Infection of the necrotic tissue investigated in 61 patients was present in 83.6% (n=51), 72.5% of which were monomicrobial and was related to early laparotomy in 57% (n=29). The overall mortality rate was 26.4%. In patients with severe pancreatitis (APACHE II>9) undergoing delayed necrosectomy mortality rate was 18.7% compared to 76.5% mortality associated with early laparotomy.

Necrosectomy delayed until the second week is a suitable procedure and may achieve a low mortality rate in patients with severe pancreatitis with pancreatic necrosis.

FP002

### HEMODYNAMIC CHANGES IN PANCREONECROSIS

V A Koubishkin, V D Fedorov

A V Vishnevsky Institute of Surgery,
Moscow, Russia

We investigated 36 patients with pancreonecrosis, using radionuclear techniques, catheterising the celiac trunk and portal vein. The changes we observed do not correlate with the degree of hypovolemia, but strongly correlates with the fall in cardiac output. In patients with group II severity pancreonecrosis (L.F. Hollender, 1) we observed a mean decrease in total blood flow to the liver of 20%. In group III patients we observed a mean decrease in total blood flow to the liver of 53% in comparison with normal individuals. After 3 days of treatment there was only a mean decrease of 41% in total blood flow to the liver in group III patients. While in group II patients the results were close to the control group. After 6-7 days of treatment the results were very close to those in patients with group I disease. From our data the main reason for decrease in total blood flow to the liver were portal hypertension with increase in pressures of 2,39±0,42 kPa (in group II patients) and 3,05±0,38 kPa (in group III patients). The signs of portal hypertension as well as thrombosis of splenic vein and migration of the thrombus were observed at angiography. In our opinion hypoxemic damage to the liver played a bigger role than enzymatic aggression, in causing hepatocellular failure, according to biochemical parameters we noticed in all patients. We used local fluid infusion through celiac trunk and portal vein for treatment and prophylaxis. This method has improved the outcome of hepatocellular damage and reduced the incidence of paralytic ileus. But there was no reduction in suppurative complications and mortality in patients without operative drainage.

Ref: Hollender L.F. et al. Akute Pankreatitis.München, Baltimor, 1983.

FP003 # TOTAL COLECTOMY FOR THE MANAGEMENT OF NECROTIZING PANCREATITIS

S Smyrnis, T Gyomisis, A Babos, A Koutsikou
M Pappa, B Nikolis, N Mamoulakis

Surgical Department, Hippokration Hospital
Athens, Greece

The clinical course of necrotizing pancreatitis and endotoxaemia show similarities, and, as it is known, the gut contains large amounts of Gram - negative bacteria that continuously shed endotoxins. Using the Limulus Lysate Test in 1976 we found endotoxin in the ascitic fluid in cases of this pathology. On the other hand enteric micro-organisms are frequently isolated from different tissues in pancreatitis. All these have raised the hypothesis that the intestinal tract may play a major role in the pathogenesis of over-whelming septic complications which have been recognised as a primary cause of morbidity and mortality in necrotizing pancreatitis. So, on the basis of this in a session of the Hellenic Surgical Society in 1986 I stated: "I wonder whether a total colectomy has a therapeutic place in necrotizing pancreatitis".

In November 1990 I performed the first total colectomy in a lady 82 years old with pancreatitis and in a situation of multiple organ failure. She had a very smooth postoperative course and left the hospital in good condition after three weeks. Since then six more patients have been operated on and a total colectomy done. Our own policy is:
cholocystectomy and decompression of a dilated common bile duct if stones are present; total colectomy with an ileostomy and a mucus fistula of the sigmoid colon; scholastic removal of the great omentum and also of the mesocolon from its root. We leave the pancreas alone because the problem is the enzymatic secretion from the "sound" pancreatic tissue and not the necrotic pancreas.

In all seven patients the operative evidence of necrotizing pancreatitis was clear-cut. They recovered very soon and five left the hospital earlier than one month. One died from pulmonary embolism 17 days after operation. Another developed an iatrogenic enteric fistula at the time of drainage of a subhepatic abscess and, during the conservative management, a metabolic disorder which had been overlooked also developed and the patient died the 50th postoperative day. Independently of these two deaths, both the results and also that of the origin of endotoxin and bacteria in the gut, strongly support an indication to total colectomy in cases of necrotizing pancreatitis until some effective measures against endotoxin are discovered.

FP004

### PERCUTANEOUS DRAINAGE OF PANCREATIC ABSCESSES

E Bloom, C H Julio, P Maquin
B Pradere, J L Gouzi

CHU Purpan, Place du Dr Baylac, Chirurgie Digestive

31059 Toulouse Cedex, France

This retrospective study was aimed to assess the efficiency of percutaneous drainage of infected pancreatic necrosis.

From 1987 to 1992, 43 consecutive patients were treated for unequivocal severe acute pancreatitis with documented necrosis on CT with dynamic pancreatography. Ranson's bioclinical criteria were available in 29 patients, with an average 4.3 score. Six patients died by multiple organ failure within the first 48 hours in the I.C.U.

In the remaining 37 patients, CT scans were graded according to BALTHAZAR's classification. Sixteen patients were grade E, 13 were grade D, and 8 were grade C.

All patients underwent transcutaneous fine needle aspiration (FNA) for bacterial culture.

Eight patients demonstrated a nonseptic clinical course with negative FNA cultures. All of these patients with non infected necrosis survived with supportive treatment alone.

Among the 29 patients with positive FNA, 11 underwent surgical exploration and drainage : 5 of these patients died. Eighteen patients were treated by **percutaneous drainage**. From 1987 to 1990, we used the Van Sonnenberg 12-14 F sump drains in 7 patients, 3 of whom were subsequently operated on, with three deaths in this subgroup.

Since July 1990, 11 patients were treated by 24-F bore lavage catheters providing an immediate evacuation of large debris. All of these patients recovered without complementary surgery despite 3 colonic fistulas and one biliary fistula at the time of percutaneous drainage.

FP005 **ENDOSCOPIC SPHINCTEROTOMY LEAVING THE GALLBLADDER 'IN SITU' AS A DEFINITIVE TREATMENT FOR ACUTE GALLSTONE PANCREATITIS IN THE HIGH RISK PATIENT.**

EM Targarona, I Pros, MJ Pons, J Martinez, C Balagué, RM Perez Ayuso, E Ros, JM Bordas, J Teres, M Trias.
Service of Surgery and Gastroenterology and Endoscopy Unit. Hospital Clinic, University of Barcelona, Barcelona, Spain.

Cholecystectomy is the best stablished treatment for acute biliary pancreatitis ( **ABP**). Morbidity and mortality after cholecystectomy increases with age and associated diseases. In recent years, endoscopic sphincterotomy leaving the gallbladder 'in situ' ( **EE-GiS**) has been considered as an useful option in high risk patients with choledocholithiasis. Nevertheless, is not known the efectiveness of **EE-GiS** in **ABP**. EE may facilitate the passage of stones and papillary obstruction could be prevented. **AIM.** To study the eficacy of the **EE-GiS** as a definitive treatment of **ABP** in high risk patients. **MATERIAL AND METHODS.** 23 patients were treated with **EE-GiS** between 1988 and 1992. Mean age was 78±8 years. All patients presented a mild pancreatitis (Glasgow score <3), and **ABP** was dignosed by biological and sonographic studies. Risk factors were age> 70 y (80% patients), or associated medical diseases (20%). **EE-GiS** was performed 15±9 days (3-25 d) afetr the onset of **ABP. RESULTS.** ERCP demonstrated a dilated bile duct in 70% of cases, and bile duct lithiasis in 9 patients (39%). EE could be done in 21 patients (91%). Morbidity was 8% and mortality 4%. 3 patients were operated, 2 for technical failure (billroth II, residual stone after EE). A third patient was operated because a gallbladder cancer was found. All patients remain asymptomatic with a follow up of 23±15 (2-51) months, without relapsing of **ABP** or symptoms secondary to gallbladder stones. 1 patient died for a non related biliary disease. **CONCLUSION: EE-GiS** may be considered as an useful alternative to conventional surgery for definitive treatment of **ABP** in the aged or high risk patient.

FP006

### LONGITUDINAL PANCREATICOJEJUNOSTOMY IN THE TREATMENT OF CHRONIC PANCREATITIS

F Callejas Neto, J C Pareja, L S Leonardi

Department of Gastroenterology Surgery
University of Campinas, Campinas-SP, Brazil

From July 1982 to July 1992, 80 patients underwent longitudinal pancreaticojejunostomy for surgical treatment of chronic pancreatitis. There was a predominance of male (94%) and alcohol-abusers (96%). Clinical findings were intractable pain by medical treatment (100%), Steatorrhoea (23,7%) and diabetes (12,5%). The surgery was indicated due to pain symptoms (100%), pancreatic cysts (63,7%) common bile duct obstruction (28,7%), pancreatic fistulas (8,7%), haemorrhage (7,5%) and duodenal obstruction (3,7%).

The postoperative complications were anastomosis fistulas (6,2%), wound infection (3,7%), pneumonia (3,7%), intra-abdominal abscess (2,5%). Thirty-day mortality was 2,5%. Follow up of 60 months showed persistence of pain (13,7%), exocrine insufficiency (31,2%), endocrine insufficiency (20%) and mortality (7,1%).

FP007

### SURGICAL MANAGEMENT OF PSEUDOCYSTS IN CHRONIC PANCREATITIS.

R.C.N. Williamson

Department of Surgery, Royal Postgraduate Medical School and Hammersmith Hospital, London

Most pseudocysts in chronic pancreatitis are intrapancreatic and associated with parenchymal destruction; treatment is controversial. A personal series of 169 patients with chronic pancreatitis undergoing operation between 1978-1992 included 72 with recurrent pseudocyst (42%). There were 55 men (76%) and 17 women, age 15-75 yr (mean 41 yr). Aetiological agents were alcoholism in 50 (69%), recurrent acute pancreatitis (9) and unknown (13). Cysts arose insidiously (57) or followed an acute exacerbation of pancreatitis (post-necrotic) in 15. Multiple in 16 (22%), cysts were sited in the head (27), neck (17) and body/tail (44) and contained between 20-1600 cc fluid. Complications in 24 patients (33%) were bleeding (6), infection (5), persistent fistula following percutaneous drainage (5), jaundice (4), gastric outlet obstruction (2) and pancr
eatic ascites (2). Drainage procedures were performed in 28 patients (Roux-Y cyst-jejunostomy 25) with concomitant drainage of the pancreatic duct in 12; there were two re-operations but no deaths. Resection of the cyst was carried out in 44 patients by distal (29), proximal (9) or total (6) pancreatectomy; there were three re-operations and one death (overall mortality 1.3%). Recorded blood loss varied from 300 ml to 12 L and was generally greater after resection. Larger, multiple and post-necrotic cysts are mostly selected for internal drainage, whereas those replacing the body and tail are treated by distal pancreatectomy.

FP008

### HAEMORRHAGIC COMPLICATIONS IN CHRONIC PANCREATITIS

F Callejas Neto, J C Pareja, E A Chaim,
S C P Leão, V F Pilla, L S Leonardi

Department of Gastroenterology Surgery,
University of Campinas, Campinas-SP, Brazil

We present our experience in the management of a group of 10 patients who developed haemorrhagic complications due to pseudo-aneurysms secondary to alcoholic chronic pancreatitis. The bleeding was located in pancreatic cysts in four patients, in the digestive tract in four patients and in the peritoneal cavity in two patients. The surgery was on an elective basis in six cases and emergency in four cases. Surgical procedures performed were distal pancreatectomy (50%) and ligation of the artery with pseudo-aneurysm associated with longitudinal pancreaticojejunostomy (50%). There was no operative mortality and no haemorrhage recidive in the follow-up at 36 months. In selected cases the combination of ligation and pancreatic drainage could be performed at the same time to manage the pain and the haemorrhagic complication of chronic pancreatitis.

FP009

### SURGICAL TREATMENT OF HEPATIC HYDATID DISEASE

P Chervenkov, R Madjov, R Radev and N Tzolov

Department of Surgery, Med University,
Varna, Bulgaria

Hepatic echinococcosis is a widespread zoo-anthroponous disease in the Balkans and our country. For the last two years there is a tendency towards increasing an incidence, especially those with complicated cysts. For the last 5 years, 101 patients with hepatic echinococcosis were hospitalised in our Clinic, 43 male and 58 female (mean age - 50,1). Most common localisation of hydatid cysts was right hepatic lobe (72 patients) left hepatic lobe (14 patients). In 6 patients both lobes were affected. In 7 cases hydatid cysts were diagnosed simultaneously in patient's liver and lungs. Solitary cysts were found in 74 patients, two cysts in 18 and 6 patients were with numerous cysts.

Most common signs were: heaviness and discomfort in the right hypochondrium (57,4%) pain (31,6%) dyspepsia (40,6%), palpable mass (18,6%), fever (14,6%), jaundice (12,7%) asymptomatic (16,9%).

Complications in our patients were: suppuration (11) rupture into biliary tree or peritoneal cavity (10) jaundice (13), biliopleural fistulas (3).

US and CT scan were most useful diagnostic methods. The following operative procedures were performed: echinococcectomy (63), echinococcectomy and cholecystectomy (9), liver resection (6), pericystectomy (3), echinococcectomy and cholecystectomy and T-tube drainage (5), echinococcectomy and cholecystectomy and choledochoduodenostomy (6), thoracotomy and diaphragmotomy and echinococcectomy and lobectomy (5).

Post operative mortality - one patient (diffuse peritonitis, caused by rupture of purulent cyst).

The results suggest that surgical treatment of hepatic hydatid disease should be governed by the size, location, number and complications of the cysts.

FP010

### THE SURGICAL MANAGEMENT OF THE HYDATID DISEASE OF THE LIVER. OUR EXPERIENCE OF 50 CASES

P.Alfaras,S.Baratsis,K.T.Delis,

S.Drakopoulos,M.Stoccos,

A.Papakonstantinou,E.J.Hadjiyannakis.

A'Surgical Department of the GH
"The Evangelismos of Athens".

Hydatid disease,caused by the tapewarm Echinococcus Granulosus,is characterized by worldwide distribution and multilocular involvement,showing a prevailling occurrence in the liver.In this paper we aim at presenting our experience from the surgical treatment of 50 patients with Hydatid disease of the liver,who have been managed in our department through the years 1986--1991.

Our group of patients consists of 28 males and 22 females their ages ranging from 15 to 81 years old (mean 52,8).Solitary cysts were encountered in 25 cases, multiple cysts in 14,whereas multilocular abdominal dissemination,including the liver,in 11 subjects.Management of the uncomplicated liver cysts (17),was provided as follows.In 5 cases the cysts were totally excised, with relative ease, but in another 5 patients atypical hepatectomy was inevitable.The residual cyst space in the remaining 7 was managed by removal of all living cyst elements, ligation of the leaking bile radicles,sterilization of the cavity and drainage by tube placement.13 of the 33 complicated cysts had ruptured into the bile ducts necessitating,beyond the aforementioned treatment,as for simple cysts,cholecystectomy,bile duct exploration and T tube insertion. In all patients with infected cysts(20),the cavities were treated as it is mentioned above,but we proceeded with omentoplasty and drained both the residual space,by placing a three way Folley catheter,at the subhepatic pouch,by fixing an elastic tube (pen-rose).

Our results have been mostly unincidental postoperatively demanding no reoperation in all cases but one, where further drainage had to be instituted.We experienced only 1 (2%) casualty. Presentation of our technique and results are elaborated, along with a brief review of the updated literature.

FP011

### HEPATIC HYDATIDOSIS. ARE DRAINAGE OPERATION JUSTIFIED?

C Vagianos, E Tzorakoleftherakis, J Petrochilos, C Panagopoulos, S Kakkos, J Androulakis

Department of Surgery, Rion Hospital, University of Patras, Patras, GREECE

Surgery offers the only possibility for cure in hepatic hydatidosis (HH). Proposed operations are divided in "radical", including removal of the pericyst (pericystectomies, hepatectomies) and "conservative", including drainage operations. Conservative operations are criticized for inferior results as for early (sepsis, biliary fistulas) and late (recurrence) postoperative complications.

From 1985 to 1991 we treated 83 consecutive patients with HH, exclusively by drainage operations. (Isolation of the peritoneal cavity, wide unroofing of the cyst, careful evacuation of the cyst contents, sterilization with hypertonic saline, interlocking haemostatic suturing of the cyst edges and postoperative drainage with closed drainage systems). Omentoplasty was added in 27 patients. Cholecystectomy was performed in 33 (40%), when biliary pathology coexisted. Intraoperative cholangiogram, performed in all cholecystectomized patients, yielded true, choledochal pathology, of 12%, treated by common duct exploration.

There was postoperative mortality of 1% (cerebrovascular accident) and morbidity of 12% (two abscesses, eight biliary fistulas). One abscess was treated by surgical and one by percutaneous drainage. Three fistulas were treated expectantly (healing required three to 12 months) and five by endoscopic sphincterotomy and nasobiliary tube drainage (healing required 7 to 14 days). No statistical improrement was shown by adding an omentoplasty. There was a recurrence rate of 8% (follow up from one to seven years, achieved in 50 patients).

Conclusion : Drainage operations, when carefully performed, achieve very satisfactory results with minimum perioperative risks. Omentoplasty does not further improve these results, while biliary complications can be successfully treated endoscopically.

FP012 **TACHOCOMB, A NOVEL COMBINATION OF A COLLAGEN FLEECE WITH FIBRIN GLUE: A NEW APPROACH IN LIVER SURGERY.**

**Dr. E. Samhaber**
HAFSLUND NYCOMED PHARMA AG
International Medical Affairs
Triesterstr. 50, A-1100 Vienna / Austria

A combined application of sheets of collagen covered with freshly prepared fibrin glue improved local haemostasis to a great extent. Large areas of capillary bleedings can be treated successfully. But due to the relatively complicated preparation required at the operation site, this method has not been used on a large scale. These drawbacks have been overcome with the latest development in this field - a sheet of collagen covered with a fixed layer of the solid components of fibrin glue (fibrinogen, thrombin and aprotinin).

In a prospective study, 225 cases of liver resections due to metastases (43 %), primary liver carcinomas (16 %), intraoperational injuries of Glisson's capsule (10 %), liver ruptures, benign tumours, bleeding subsequent to punch biopsies, echinococcus cysts and others were included. The assessment of the haemostyptic properties of TachoComb was "very good" and "good" in 95 %, "satisfactory" in 4 % and unsatisfactory in 1 % of the cases.

In several cases, particularly difficult bleeding situations could be controlled, e.g. those with massive coagulation disturbances or hepatic stasis. No complications attributable to TachoComb occurred.

So far, more than 280 patients with liver resections have been treated successfully with TachoComb in clincal trials. In a liver transplantation study with 27 patients, haemostasis with TachoComb was assessed as "very good" and "good" in 96 % of the cases.

FP013

### PREDICTION OF BLOOD TRANSFUSION DURING LIVER RESECTION

G Borgonovo, N Bourokba, C Vons, D Mariette, C Smadja, D Franco

Service de Chirurgie, Hôpital Antoine Béclère, Clamart, France

Liver resection are often associated with operative bleeding and blood transfusion. The purpose of this work was to determine which patients were particularly at risk of intraoperative blood transfusion. Between October 1990 and July 1992, 100 patients had liver resection for a liver tumor : hepatocellular carcinoma in cirrhosis (37 patients), primary liver cancer in normal liver (14 patients), liver metastases without previous chemotherapy (16 patients) or following chemotherapy (3 patients) and benign liver tumor (30 patients). There were 12 atypical resections, 39 segmentectomies, 25 major hepatectomies, 15 extended hepatectomies and 9 liver resections associated with portal, IVC or biliary reconstruction. Clamping of the portal triad was used in all patients and hepatic vascular exclusion in one patient with IVC replacement. Twenty two (22 %) patients required blood transfusion. It was significantly ($p < 0.01$) more frequent in patients with cirrhosis (41 %) or following chemotherapy (66 %) than in patients with a normal liver (4.5 %). The rate of blood transfusion was almost similar during atypical (17 %), segmental (5 %) or major (16 %) liver resection. It was slightly higher after extended (40 %) and significantly higher during resection with associated procedures (89 % $p <$ O.O1). The mean volume of blood transfusion per transfused patient was around 5 units of blood whichever tumor was resected and whichever resection was performed. These results suggest that blood transfusion is not frequent in patients will a normal liver undergoing liver resection when no associated procedure is performed. Autotransfusion, hemodilution, hepatic vascular exclusion (HVE) are not required in such cases. Blood transfusion is frequent and abundant in cirrhotic patients and patients with chemotherapy undergoing an extended liver resection or a liver resection associated with reconstruction of the portal vein, IVC or bile duct. Only in such patients a combination of HVE, hemodilution and autotransfusion might decrease transfusional needs.

FP014

### 3-D DISPLAY OF INDIVIDUALIZED LIVER ANATOMY

MS van Leeuwen[1], MA Fernandez[1], HW van ES[1].

RS Stokking[2], HG van Meurs[2].

H Obertop[3], AH Hennipman[3],

ThJMV van Vroonhoven[3].

Department of Radiology[1], Computervision[2],

Department of Surgery[3]. University Hospital

Utrecht, Netherlands.

MATERIAL and METHODS

In anatomical liver resections the intrahepatic vascular anatomy and tumors have to be related to the external landmarks on the liversurface in order to define the precise extent of the planned resection.

The relation between internal anatomy and external landmarks on the liversurface was studied in 10 volunteers, using 3-D reconstructions of MRI acquisitions.

RESULTS

Two out of 10 subjects showed a normal pattern of 3 hepatic veins, middle and left vein sharing a common trunk. The remaining 8 subjects had 9 accesory hepatic veins, 4 right, 2 middle and 3 left. Additionally, 1 subject had a separate origen of the left hepatic vein and right hepatic vein.

Two out of 10 subjects showed a conventional portal branching pattern with a separate right anterior and posterior trunk. Two subjects had a separate branch to the right liver which originated from the main portal vein before it's division in right and left portal trunk. 4 subjects showed a common trunk to segment 5 and 6 and 4 others showed a trunk to segment 7 and 8.

On average the gallbladder was located 6 degrees to the right of the middle hepatic vein (range 1-18). The right hepatic vein was allways located in the coronal plane or slightly posterior to it. The transverse fissure could not be assigned in 3 out of 10 patients due to the presence of more than one right portal trunk.

CONCLUSION

Marked individual variations in hepatic and portal vein anatomy, known from in vivo and in vitro studies, can be shown with 3-D liver imaging, thus providing the possibility of an individualized preoperative planning procedure.

FP015

### REMOTE RESULTS OF SHUNTING EFFLUENT PANCREATIC BLOOD OPERATION IN INSULIN-DEPENDENT DIABETIC PATIENTS

E I Galperin, T G Diuzheva, P F Petrovskyi, S E Rabinovich, A U Tchevokin, L V Platonova

Moscow Medical I M Sechenov Academy, Russia

In 409 patients with insulin-dependent diabetes mellitus, distal splenorenal venous shunt was made to divert pancreatic blood rich with hormone glucagon into the general circulation to meet injected insulin (ins) in the peripheral tissues. After the operation the ratio of exogenous insulin and endogenous glucagon in the tissues (hepatic and peripheric) seems to be more optimal for their interaction. Angiographic control was made in 137 of them at a period of 8 months after surgery and included renal venography, selective splenic venography. In 114 patients (83%) splenorenal shunt proved to be permeable and in 23 patients (17%) opacification of the splenic vein was not achieved after using all the above methods.

After the shunting operation, the patients with permeable anastomosis showed disappearance of, or diminishing complaints of leg pains, general weakness, clinical manifestation of hypo- or hyperglycaemia. The dose of injected exogenous ins. reduced from 0,95 $\pm$ 0,05 Un/kg to 0,76$\pm$ 0,04 Un/kg ($P<0,05$) without changing the preparation and schedule of injections. In 16 patients improvement took place without changing the dose of exogenous ins. The level of glycosilated haemoglobin ($HbA_{1C}$) was 9,3$\pm$ 0,3% against 13,3$\pm$ 0,3 prior to surgery ($P < 0,05$). At the same period of time after the operation patients with non permeable anastomosis had the same complaints and the same dose of ins. as before surgery. The level of $HBA_{1C}$ remained high: 12,8$\pm$0,3%. It was proved that development of splenorenal collaterals in patients with permeable anastomosis reduced the efficiency of the shunting procedure, therefore we now perform additional ligation of vv. gastrica sin. and gastro-epiploica sin. Hepatic function did not suffer after surgery.

Stabilization of clinical state and metabolism after the operation and dependence of remote results on permeability of applied anastomosis witness efficiency of the new approach to treatment of insulin-dependent diabetes mellitus.

FP016 HUMORAL FACTOR(S) ASSOCIATED WITH ACUTE PANCREATITIS INDUCE LEUKOAGGREGATION

KS Guice, KT Oldham
Divisions of General Surgery and Pediatric Surgey
Department of Surgery
Duke University Medical Center, Durham, NC 27710

Local and remote tissue injury results from the development of acute pancreatitis. In particular, the sequestration of neutrophils (PMNs) occurs at sites of capillary endothelial cell injury in the lung and pancreas. Other data from our laboratory indicate a pathogenic role for these PMNs. It is not known whether this PMN sequestration results from specific humoral factor(s), or from alterations in the target endothelial cells, or both. This study was designed to assess whether circulating plasma in animals with acute pancreatitis contains humoral factor(s) which enhance PMN adhesion. METHODS: Secretagogue-induced acute pancreatitis was generated in 200 gram Sprage Dawley rats by continuous intravenous infusion of supra-physiologic doses (5μg/kg/min) of caerulein, a CCK analog. Blood was obtained after 3 hours by venipuncture from the inferior vena cava and plasma PMN aggregation potential was quantitatively determined electronically with a Coulter™ Counter by a method previously developed and described.

RESULTS

| GROUP | MEAN ± SE |
|---|---|
| C - 15 min | 9.80 ± 3.4 |
| NS - 15 min | 12.2 ± 2.3 |
| C - 30 min | 34.8 ± 8.0 * |
| NS - 30 min | 2.20 ± 1.2 |
| C - 60 min | 43.9 ± 13.7 * |
| NS - 60 min | 8.50 ± 7.5 |

*p<0.05 compared to time matched NS control
(C = Caerulein infused; NS = Normal Saline infused)

Conclusions:
Plasma from rats with caerulin-induced acute pancreatitis has significantly greater leukoaggregation potential than plasma derived from saline infused control animals. These data offer compelling evidence for the presence of humoral factor(s) which enhance PMN adhesion following acute pancreatitis. The nature of these factors is the subject of ongoing investigation.

**FP017** THE INFLUENCE OF SERUM-ENDOTOXIN ON THE PROGNOSIS OF ACUTE EXPERIMENTAL PANCREATITIS

U. Sulkowski, C. Boin, J. Brockmann, A. Holzgreve

Department of General Surgery, Münster University Hospitals, Germany

In a controlled prospective randomized trial serum-endotoxin levels were measured after induction of acute experimental pancreatitis and synchronous colonic irrigation or creation of a cecostomy. Thus, after inducing acute pancreatitis in male wistar rats by injecting a 2% taurocholate-solution into a temporarily closed duodenal loop according to a technique first described by Orda and co-workers (Arch. Surg. 1980), 40 animals underwent cecostomy (group B), in 36 the colon was irrigated with normal saline (group C), and 36 served as controls (group A). Altogether, twelve animals succumbed intra- or immediately postoperatively. As a consequence 31 remained in group A, 36 in group B, and 33 in group C. Before the end of scheduled follow-up (15 days) the mortality rate in the control group was 22.6%, 16.7% in group B and 9.1% in group C, with the difference between group A and C being statistically significant at $p < 0.05$. This difference was parallelled by a significant difference of serum endotoxin levels between the groups (219 ng/l in group A, 79.2 ng/l in group B, and 71.7 ng/l in group C). Also the number of serum endotoxin positive animals was significantly higher in group A than in group B and C (7 in group A vs. 2 in group B and C). Our results suggest that endotoxin might play an important role in the pathophysiology of acute pancreatitis and that endotoxin absorption might be reduced by cecostomy or colonic irrigation.

FP018

### EFFECT OF L-GLUTAMINE ON ESSENTIAL FATTY ACID-INDUCED DESENSITIZATION OF PANCREATIC BETA CELLS

EC Opara, M Garfinkel, SK Lee, OE Akwari

Department of Surgery, Duke University Medical Center, Durham, NC 27710, USA

The essential fatty acid, linoleic acid, constitutes about 60-70% of the clinically available lipid emulsions, which are now routinely provided to patients receiving parenteral nutrition to avoid the development of essential fatty deficiency. However, it has been shown in experimental models, that the infusion of polyunsaturated fatty acids (PUFA) into rats and isolated perifused islets, will stimulate insulin secretion, but will render the beta cells unresponsive to glucose. The mechanism of both the stimulatory and desensitization effects of PUFA was shown to be linked to fatty acid oxidation. The AIM of the present study was to explore the possibility of restoring the glucose effect by the provision of L-glutamine, a major fuel source and a precursor for the biosynthesis of the antioxidant, glutathione (GSH) to the PUFA perifusate of isolated islets. METHOD: In each experiment, a batch of six islets microdissected from three female CD-1 mice were preperifused for 1 hour at 37°C at the rate of 1ml/min with a Krebs-Ringer bicarbonate buffer, containing 5.5mM glucose (basal), 2% bovine albumin, 100 KIU/ml trasylol and maintained at pH 7.4 by continuous gassing with 95%/5% $O_2/CO_2$. Basal effluent samples were then taken before the glucose concentration was raised to 27.7mM for 20 minutes; immediately followed by 20 mins basal glucose "washout" in the absence or presence of 20mM glutamine (GLN) or 3mM glutathione (GSH). Linoleate (10mM) was then added to the basal perifusate without or with GLN or GSH. Effluent perifusate samples were collected on ice at 2 min-intervals and stored frozen until radioimmunoassay for insulin. RESULTS: The stimulatory effect of 27.7mM glucose on insulin secretion assessed as the incremental area under the curve were 1552.8 ± 276.3 and 220.4 ± 163.9 pg/20 mins ($p<0.001$, $n=6$) respectively, before and after linoleic acid treatment alone. In experiments, in which islets were treated with the fatty acid in the presence of L-glutamine, there was no difference in the insulin response to 27.7mM glucose before and after linoleate treatment (2051.8 ± 420.5 vs 2159.2 pg/20 mins respectively, $n=6$). When GSH was substituted for L-glutamine, qualitatively similar results were observed. CONCLUSION: The presence of glutamine or GSH completely blocked the linoleate-induced desensitization of beta cell secretory response to glucose, suggesting similar a mechanism between their actions. These data support an efficacious role for L-glutamine during hyperalimentation.

FP019

### SURVIVAL FOLLOW UP OF A RAT PANCREATIC CANCER TREATED BY PHOTODYNAMIC THERAPY

S Evrard[1], P Keller[1], G Balboni[2], C Damgé[2], M. Aprahamian[3] & J. Marescaux[1]

1: Department of Surgery A, University Louis Pasteur, Hôpital Civil, BP 426, F - 67091 Strasbourg Cedex; 2: INSERM, Unit 61, 3 avenue Molière, F - 67200 Strasbourg

At the present time surgical resection is the sole chance of cure in pancreatic cancer but only for 5-10% of the patients. Efficiency of surgical resection could be perhaps enhanced by an adjunctive intraoperative photodynamic treatment. The aim of this work was to study the survival of a model of rat pancreatic cancer treated by Pheophorbide A (PPA) photodynamic therapy (PDT).

The tumoral model was an acinar pancreatic cancer induced by azaserine and transplanted in the pancreatic tail of Lewis rat. The survival time of this model do not exceed 33 days. The photosensitizer used (PPA) was a chlorophyll derivative at a dosage of 9 mg. $kg^{-1}$ intravenously administered 24 hours before illumination and achieving a ratio of 12:1 between tumor vs surrounding normal pancreas. A DCM laser pumped by a copper vapor laser was used to deliver a fluence of 100 $J.cm^{-2}$ at 660 nm. The spot size of laser light was 3 $cm^{-2}$. The tails of the pancreas (bearing a tumor of 1 cm in diameter) of 18 rats (9 with PPA and 9 without) were exposed under a midline laparotomy. The surface temperature of the tumor was monitored throughout the illumination. The maximum tumor temperature rise was less than 2 °C. This elevation was not considered in favour of thermal effects. Rats follow up was established according with Kaplan Meier method. Autopsies were performed on dead rats. Rats surviving more than 4 months were sacrificed for histological examinations.

Control rats (9 illuminations without PPA, 9 PPA without illumination and 9 without any kind of treatment) did not survive more than 33 days with evidence of metastatic spread and carcinomatosis. At this time, the rate of survival in the PDT group was 89% (log rank test, $p < 0,001$). Six animals (66%) were still alive 4 months after PDT. Histological examination of the survivals showed evidence of pancreatic tail necrosis without metastatic spread.

Further experiments were conducted histologically in order to determine the threshold of PDT damage. 33 and 75 J. $cm^{-2}$ induced a surrounding œdema of pancreatic acini but failed to destroy tumor cells. Tumor destruction was achieved only at a fluence of 100 J. $cm^{-2}$ . It should be observed that normal pancreas was immune to PDT damage even at a fluence of 100 J. $cm^{-2}$.

The efficiency of photodynamic therapy using pheophorbide A was assessed not only by histological selective necrosis of pancreatic tumor but also by 2/3 survival of PDT rats. This work suggests that intraoperative adjunctive PDT may improve therapeutic results of pancreatic carcinoma surgery.

FP020 **CHLOROQUINE ATTENUATES THE INTENSITY OF EDEMATOUS ACUTE PANCREATITIS IN RATS**

**A L Montagnini, M S Kubrusly, R Yamauchi, A M M Coelho, A Bonizzia, S N Sampietri, M C C Machado**

**Department of Gastroenterology, Hospital das Clinicas, São Paulo University, São Paulo, Brazil**

Trypsinogen activation by cathepsin B in acinar cell zymogen occurs only at low pH (3-4) and is an important step in the initiation of acute pancreatitis.

**Aim:** Determine if chloroquine, a lysosomotropic agent that rises intra zymogen pH, can alter the intensity of edematous acute pancreatitis induced by supramaximal doses of cerulein.

**Method:** During 3 days 25 male Wistar rats (220 - 250 g) received by gastric tube diphosphate chloroquine 50mg/kg body weight/day (Group I) or saline solution 2ml (Group II) and stayed under overnight fasting between days 2 and 3. At day 3, two hours after the last dose Group I and Group II rats were given a subcutaneous injection of cerulein (20µgr/kg body weight) and one hour later an intravenous injection of cerulein (20µgr/kg) and Evan's blue (20µgr/kg). Rats were sacrificed after one hour and the pancreas isolated for water and Evan's blue determination. The amount of dye in pancreatic tissue correlated with pancreatic edema. A control group (III) was included in the study. Group III rats received saline solution instead of cerulein.

**Results:** mean ± SEM

| | Group I<br>n=11 | Group II<br>n=14 | Group III<br>n=19 |
|---|---|---|---|
| % Water | 78,5±1,5 | 84,0±1,0# | 72,4±0,7*** |
| Evan's Blue<br>µgr/gr dried tissue | 197,5±30,0 | 302,1±30,1£ | 69,3±7,3°* |

(*)P<0,05 vs GI (**)P<0,01 vs GII (#)P<0,01 vs GI (°)P<0,01 vs GII (£)P<0,05 vs GI

**Conclusion:** Chloroquine attenuates the intensity of edematous acute pancreatitis induced by cerulein in rats. The alkalinization of zymogen granule and lower activity of cathepsin B may play a role in this protective effect.

FP021

### PHOTODYNAMIC IMAGING (PDI) OF A RAT PANCREATIC CARCINOMA USING PHEOPHORBIDE A (PPA)

P. Keller[1], F. Heisel[2], S. Evrard[1], C. Damgé[3], M. Aprahamian[3] & J. Marescaux[1]

1: Department of Surgery A, Hôpital Civil, BP 426, F-67091 Strasbourg Cedex. 2: GOA, CNRS, 3: INSERM, Unit 61.

The use of optical spectroscopy is a potential new approach for the diagnosis of pancreatic malignant tumours. We will report the use of laser induced fluorescence of a new photosensitizer (PPA) which presents tumour-localising properties.

The tumoral model used in this experiment was an acinar pancreatic cancer induced by azaserine and transplanted in the pancreatic tail of Lewis rat. The PPA is a chlorophyll derivative used in our experiment at a dosage of 9 mg. $kg^{-1}$ IV 24 hours before illumination.
Fluorescence emission spectra of 5 tumours and their surrounding pancreas obtained under 400 nm excitation showed a broad double peak with a maximum at 678 nm. Autofluorescence from tissue was also observed at 463 nm. A reduction of PPA fluorescence intensity was found at the surface of the tumours because of their heavy blood staining. Consequently, PPA fluorescence signal alone was unable to provide a photodynamic image of pancreatic carcinoma.
In order to avoid problems related to variations in distance, surface topography, drifts in laser intensity, detection efficiency and overall cancer blood staining, we have developed a concept based on a dimensionless function. This function was established by dividing the intensity fluorescence signal of PPA by autofluorescence. We called it *Rt* for the tumour and *Rp* for the surrounding pancreas. The fluorescence contrast $C = Rt / Rp$ should be greater than one to allow obtaining of PDI of a tumour.
To determine the best excitation we have tested the previously established excitation wavelengths of PPA (355, 400, 510, 530 and 610 nm) on 5 tumours and surrounding pancreas. Obviously, 355 nm gave the best fluorescence contrast ($C = 1.5$) and was used to perform our preliminary imaging of an intrapancreatic tumour and its intraperitoneal metastasis.
We used a Nd : YAG laser at 355 nm as excitation source. Fluorescence was recorded by a CCD camera through 2 interferential filters: in the red for PPA signal and in the green for autofluorescence. A dimensionless contrast function has been calculated for each spatial location using the values in corresponding pixels in the two images. A resulting artificial image was formed with false colours coding. High contrast images have been obtained from the tumour and peritoneal metastasis.

PDI of pancreatic carcinoma using PPA as a dye seems to be effective. Such an imaging process may perhaps allow in the future to detect and cure the metastatic spread in the time course of debulking surgery of a pancreatic carcinoma.

FP022

### PROTECTIVE EFFECT OF REDUCTION OF PANCREATIC ENZYME CONTENT IN EXPERIMENTAL ACUTE PANCREATITIS IN RATS

A M D M Coelho, M S Kubrusly, A Bonizzia, M C C Machado

Department of Gastroenterology, Hospital das Clinicas, São Paulo University, Brazil

We have shown previously that under nutrition reduces the mortality of acute experimental pancreatitis by decreasing the pancreatic enzyme content.

Cerulein in physiologic doses reduces the enzyme content of the pancreas without any harmful effect to the pancreas.

The aim of this study was to assess the effect of acute reduction of pancreatic enzyme content by using inframaximal doses of cerulein in the outcome of acute pancreatitis (AP).

**Method:** Two groups of rats were studied: Group I- control animals fasting 12 hours and Group II- animals receiving cerulein in inframaximal doses (0,2μg/kg/h) with free access to water and food. Enzymatic content of the pancreas was studied in both groups. Acute pancreatitis was induced by retrograde injections of 0,5 ml of 2,5% Na-taurocolate into the pancreatic duct.

**Results:** Cerulein administration resulted in 71%, 55%, 60%, 62% and 41% decreases in pancreatic content of chymotrypsin, trypsin, proelastase, amylase and cathepsin B respectively ($p < 0{,}01$). Mortality was 56% in the control group and 23% in the cerulein group ($p < 0{,}05$).

**Conclusions:** These results indicate that decreasing enzymatic content of the pancreas reduces the severity and gives a protective effect in acute pancreatitis in rats.

FP023

### LIVER TRANSPLANTATION WITH OR WITHOUT OCCLUSION OF THE INFERIOR VENA CAVA COMPARISON OF TWO TECHNIQUES

D. Cherqui, N. Rotman, J.L. Hingot, J.Y. Lauzet, D. Dhumeaux, M. Julien, P.L. Fagniez

Hôpital Henri Mondor 94010 Créteil, France

Regular orthotopic liver transplantation (OLT) technique includes occlusion of the inferior vena cava (IVC) and portal vein with or without use of venous bypass. A new technique was recently described permitting to avoid IVC occlusion (1). It includes : (a) recipient hepatectomy with preservation of the IVC, (b) closure of the graft IVC at its upper and lower ends, (c) side partial clamping of the recipient IVC, and (d) side-to-side anastomosis between the graft and recipient IVCs. The aim of this study is to compare the results of the regular and the modified techniques used successively at the same center.

From 1989 to 1992, 89 OLTs were performed in 88 patients. Five patients with a major technical variant were excluded (2 clusters and 3 reduced-sized grafts from a split procedure). Until September 1991, the regular technique was used in 41 patients (42 OLTs) using venous bypass in cases of hemodynamic intolerance to clamping (group 1). Since that date 42 consecutive patients were operated on with the modified technique associated to a temporary end-to-side portacaval anastomosis (group 2).

Results

| | group 1 | group 2 |
|---|---|---|
| number OLTs | 42 | 42 |
| operative time * | 9.3±1.9 | 7.5±1.7 |
| red-cell units transfused ** | 20.3±14.4 | 12.4±6.5 |
| venous bypass | 69% | 0 |
| hospital mortality *** | 2.4% (1/41 pts) | 12% (5/42) |

* p<0,01 ** p<0,005 *** NS

The use of the modified technique was possible in all cases. Early portacaval anastomosis was very useful for recipient hepatectomy. In 2 cases, the IVC had to be occluded for a 5-minute period because of hemorrhage during hepatic vein dissection. There were no deaths due to the technique used.

The modified technique allows to avoid venous bypass in all cases and to reduce significantly operative time and intraoperative blood loss. This technique has proved to be simpler and as safe as the regular one and, therefore, can be recommended.

1. Belghiti et al. Surg Gynecol Obstet 1992;175:271-2

FP024

### INCIDENCE, MANAGEMENT AND OUTCOME OF PORTAL VEIN ABNORMALITIES AT ORTHOTOPIC LIVER TRANSPLANTATION

B Davidson, A Burroughs, K Rolles

Hepatobiliary and Liver Transplantation Unit, Royal Free Hospital and Medical School, London

Partial or complete occlusion of the portal vein (PV) is considered a relative or complete contra-indication to orthotopic liver transplantation (OLT). We have analysed the incidence, management and outcome of this group of patients.

Of 140 consecutive patients undergoing OLT during the period October 1988 to October 1992 14 patients had either partial (n=7) or complete (n=7) occlusion of the PV at surgery. In 13 of the 14 portal inflow was re-established by flushing, Fogarty balloon embolectomy or passage of a graduated dilator. In one patient complete fibrous obliteration necessitated a PV to right gastro-epiploic vein anastomosis.

On follow up there have been 6 deaths in this group (6/14 = 43%) from recurrent cancer (n=1), sepsis (n=4) and cardiac and renal failure (n=1). Four of these 6 patients had confirmation of PV patency on imaging. The remaining 8 patients are alive and well (median follow up 28 months, range 1-44 months)

Further PV thrombosis (PVT) occurred in 3 of the 14 patients (21%) with PV abnormalities. At the time of surgery in one and at 7 and 12 days post op. This was successfully treated by thrombectomy in two and required re-transplantation in one with associated arterial occlusion. Of the 126 patients with a normal PV at surgery post op PVT occurred in two (2/126 = 1.6%) at 24 hours and 14 days post op and was again successfully treated by thrombectomy.

Portal vein abnormalities found at the time of OLT are common (10%) and these patients are high risk (43% mortality). Further thrombosis of PV is significantly increased in this patient group (3 of 14 vs 2 of 126. $X^2$ = 14.4, $p<0.001$)

FP025

### USE OF T-TUBE STENT IN BILIARY RECONSTRUCTION DURING ORTHOTOPIC LIVER TRANSPLANTATION - A PROSPECTIVE RANDOMIZED CONTROLLED TRIAL

V Vougas, N D Heaton, M Rela, E Gane,
R Williams, K C Tan.

Department of Liver transplant surgery and the Institute of Liver studies, King's College Hos.

Different surgical techniques have been used for biliary tract reconstruction following orthotopic liver transplantation (OLT). These techniques include the Roux-en-Y choledocho-jejunostomy, gallbladder conduit and direct ductal anastomosis.
Biliary complications range from 12%-50% depending on the technique utilized and the presence of vascular insufficiency.

Over a full year period (1992), 127 patients underwent 145 liver transplantations in King's College Hospital. From these, 58 patients have been randomized in a prospective study of their biliary reconstruction. Excluded from this study were patients with previous Roux-en-Y anastomosis and retransplants. In 28 patients (age range of 13-64 years) a T-tube was used (T-T), whereas in 30 patients (age range of 19-66 years) no stent or T-tube (NT-T) was used during a direct end to end bile duct anastomosis.
Complications occurred in 8 patients (28.5%) from the T-T group and in 4 patients (13.3%) from the NT-T group. In the T-T group 7 had early (<3 months) complications including: accidental dislodgement (2), bile leakage (3), cholangitis from bile stasis (1) and T-tube blockage (1). There was a late complication (>3 months): localized peritonitis after T-tube removal.
In the NT-T group 4 patients experienced bile duct stricture: one early and 3 in the late postoperative period. All patients were treated with endoscopic baloon dilatation but 2 required surgical reconstruction and one was retransplanted because of hepatic artery thrombosis. Fifty two patients survive a mean follow up of 5.7 months. Six patients died (10%) from causes unrelated to biliary complications.

In biliary anastomosis during OLT there was significant increase in morbidity with the use of a T-tube, when compared to those without a T-tube stent (28.5% vs 13.3%).

FP026 BILIARY COMPLICATIONS AFTER ORTHOTOPIC LIVER TRANSPLANTATION.

D Mirza, G Batignani, B Gunson, S Olliff, AD Mayer, JAC Buckels, P McMaster.

The Liver and Hepatobiliary Unit, Queen Elizabeth Hospital, Birmingham, U.K.

Despite improvements in survival after orthotopic liver transplantation (OLT), biliary complications continue to cause significant morbidity. In April 1988, we changed our technique from a gall bladder conduit to a direct donor bile duct anastomosis to either the recipient bile duct (DD), or to a Roux loop (DR). Up to August 1992, 487 OLTs were performed, of which 383 were in 339 adults (median age 48). Biliary reconstruction was in the form of DD (326), DR (56), and choledochoduodenostomy(CD) (1). Biliary complications occurred in 86/383 OLTs (22%), 12 following DR (21%), 73 following DD (22%), 1 following CD (100%).

Bile leaks occurred in 38 cases due to anastomotic leaks (17), 9 of whom subsequently developed strictures, asymptomatic leak diagnosed on protocol T tube cholangiography (9), leak following hepatic artery thrombosis (4), leak following T-tube removal (5), cystic duct leak (2), segmental hepatic duct leak (1). Biliary obstruction occurred in 48 cases due to anastomotic stricture (7), intrahepatic stricture (9), papillary stenosis/ recipient duct obstruction (8), sludge syndrome (14), hepatic artery thrombosis with stricture (3), retained T-tube/ stent (3), recipient duct calculi (2), cystic duct stump granuloma (2). Bile duct complications were not related to prolonged preservation.

Surgical treatment in the form of biliary reconstruction, was carried out in 27 patients, and one patient required retransplantation. There were 4 deaths related to biliary complications, and one graft loss. In conclusion, biliary complications continue to be a significant cause of patient morbidity.

FP027 RISK FACTORS OF LATE BILIARY COMPLICATIONS IN LIVER TRANSPLANTATION

S. Landen, J.F. Desjardins,
F. Siriser, E. Bardaxoglou,
C. Stasik, B. Meunier, B. Launois
Department of Digestive Surgery and
Transplantation Unit
Pontchaillou Hospital, Rennes, France

Improval in survival following liver transplantation has brought to light late complications, particularly those concerning the biliary tract. The aim of this study is to determine the risk factors of these late biliary complications.

Between April 1978 and June 1992, 135 orthotopic liver transplantations were performed. During the harvesting procedure (rapid technique as of 1978) the bile ducts were rinsed by injection of saline via the gallbladder. The anastomosis was choledoco-choledochal, choledoco-jejunal and cholecysto-jejunal in 102, 27 and 3 cases respectively. Three patients died intraoperatively, prior to biliary anastomosis.

Among 110 functional grafts at 1 month, 12 late biliary complications were observed (10.9%). Six of these late biliary complications consisted of an association of strictures and dilatations of the intrahepatic bile ducts. Among the group (n=12) with late biliary complications, 4 showed stenoses of the hepatic artery or one of its branches, whereas only one of the patients in the group (n=98) without late biliary complications showed arterial lesions ($p<0.001$). Excluding patients with hepatic artery stenosis or thrombosis, mean cold ischemia time of liver grafts that developed late biliary complications was $859 \pm 259$ minutes versus $583 \pm 283$ minutes for those having not developed biliary lesions ($p<0.01$). Late biliary complications were not correlated with the type of biliary anastomosis or the presence of a positive cross-match. Seven reoperations and 1 retransplantation was performed for late biliary complications.

These results suggest that prolonged cold ischemia times and reduced arterial inflow increase the incidence of late biliary complications.

FP028 Combined Heart-Liver transplantation: refinement in surgical technique.

KC Tan, ND Heaton, A Khagani*, M Pepys+, R Williams and sir Magdi Yacoub*

King's College Hospital, Harefield Hospital* and Hammersmith Hospital, London.

Combined heart-liver transplantation had been performed sporadically with mixed success. Since the first case was performed in 1984, the procedure has met with severe intra-operative difficulties such as haemorrhage and cardiac dysfunction. The main reason for these had been the simultaneous implantation of the two organs under cardio-pulmonary bypass. In order to circumvent this a staged procedure was described where the liver was grafted 2 weeks after the heart. The obvious disadvantages are that the organs were from different donors and two major surgical procedures were required.

We described a modification of the technique whereby the organs were grafted sequentially in a single operation. This was performed in two patients with great success. The first patient was a 33 year old woman with familial hypercholesterolaemia and severe coronary artery disease. the other was a 60 year old man with familial amyloidotic polyneuropathy in moderate heart failure. The latter was the first time such an opeation was performed for this condition. We encountered no intraoperative problem and except for a mild rejection in the first patient their postoperative recovery was uneventful.

FP029

### OCTREOTIDE IN THE CONTROL OF POST-INJECTION SCLEROTHERAPY HAEMORRHAGE

**S.A. Jenkins, R. Shields, A.N. Kingsnorth, R. Sutton, S. Ellenbogen**

**Department of Surgery, Royal Liverpool University Hospital, Liverpool, U.K.**

Bleeding from oesophageal ulcers, oesophagitis or from the varices themselves after injection sclerotherapy is occasionally massive and difficult to control. Since our current experience with octreotide suggest that it is a safe and effective treatment for the control of the acute variceal bleed, we have examined its efficacy in these post-sclerotherapy problems.

Seventy-seven patients experienced a significant gastrointestinal bleed (blood pressure < 100mm Hg, pulse > 100 b.p.m. or the need to transfuse 2 or more units of blood to restore the haemoglobin levels). The source of bleeding was varices in 42 patients (Child's Group A = 1, B = 9, C = 32), oesophageal ulcers in 31 (Child's A = 3, B = 3, C = 25) and oesophagitis in 4 (Child's A = 2, B = 2). All patients received a continuous intravenous infusion of octreotide (50 $\mu$g/h) for between 40 and 140h.

Haemorrhage was successfully controlled by an infusion of octreotide in all patients with oesophagitis, in 30 of 31 patients with oesophageal ulceration and in 38 of 42 patients with bleeding from varices. In the 1 patient with persistent haemorrhage from ulcers and in 2 of the 4 with continued bleeding from varices, haemostasis was achieved by hourly boluses of 50 $\mu$g octreotide for 24h. No complications were associated with octreotide administration.

The results of this study clearly indicate that octreotide is a safe and effective treatment for the control of the severe haemorrhage after technically successful injection sclerotherapy.

FP030 **ENDOSCOPIC SCLEROTHERAPY IS MORE COST EFFECTIVE THAN OESOPHAGOGASTRIC DEVASCULARIZATION AND TRANSECTION IN THE LONG-TERM MANAGEMENT OF BLEEDING OESOPHAGEAL VARICES: INTERIM ANALYSIS OF A PROSPECTIVE RANDOMIZED CONTROLLED TRIAL.**

JEJ Krige, PA Goldberg, PC Bornman, J Terblanche.
Department of Surgery and MRC Liver Research Centre, University of Cape Town and Groote Schuur Hospital, Observatory, Cape Town, South Africa.

Fifty-two patients (35 male, mean age 45.8 years, range 18 - 65 years) with variceal bleeding were randomised after emergency endoscopic sclerotherapy to continued endoscopic sclerotherapy (ES) using 5% ethanolamine until variceal obliteration followed by regular check endoscopy (n=27) or to oesophagogastric devascularization with transection (OGDT;n=25). Childs C score > 11, those over 65 yrs and high risk operative patients were excluded. Thirty-seven pts had alcoholic cirrhosis; 7 were Childs A, 26 Childs B and 19 Childs C. Mean follow-up was 19 months (range 6 - 58 months).

All data were analyzed on an intention to treat basis. Mortality during the first month after randomization was higher in the surgical group (2/25 v 0/27) but late deaths in the OGDT pts were fewer than among the ES group (5 vs 9). Varices were eradicated in 24 of 27 patients in the ES group after a mean of 5 injections (range 2-10). Three patients in the ES group died before eradication at a mean of 96 days. One patient in each group required dilatation for an oesophageal stricture. No patient died from rebleeding.

During follow-up there were no significant differences between the ES and OGDT groups with regard to number of patients rebleeding from varices (6 vs 7), number of bleeding episodes (8 vs 6) number of units of blood transfused per patient (0.7 vs 0.4), total number of hospital admissions (79 vs 70), mean number of days in hospital per pt (52 vs 45) or mean days per admission (15 vs 16). The mean cost per patient was however significantly more in the group undergoing operation (OGDT=$12,475; ES=$7,542). We conclude that while ES and OGDT are equally effective in eradicating varices and preventing rebleeding, sclerotherapy is significantly more cost effective than operation.

FP031

### SUGIURA PROCEDURE VS. PORTACAVAL SHUNT - RESULTS OF A PROSPECTIVE RANDOMIZED STUDY

G Borgonovo, D Mariette, C Vons, C. Smadja, D Grange, D Franco.

Services de Chirurgie, Hôpital Louise Michel, Evry, et Hôpital Antoine Béclère, Clamart, France.

The purpose of this work was to compare the results of Sugiura procedure (SP) and portacaval shunt (PCS) in the elective treatment of cirrhotic patients with previous variceal bleeding. Fifty-four patients were included in the study between January 1986 and April 1989. Twenty-seven patients were randomized into the PCS group and underwent a direct side-to-side portacaval shunt (22 patients), an interposed portacaval shunt (1 patient), or a mesocaval shunt (4 patients). Twenty-seven patients were randomized into the SP group and had a Sugiura procedure (26 patients) or a mesocaval shunt (1 patient). The intention to treat principle was applied to this study. The two groups were comparable according to etiology of cirrhosis, liver function tests, number and severity of bleeding episodes and size of varices. Total and variceal recurrent bleeding episodes were more frequent after SP (33 % and 22 %) than after PCS (11 % and 4 %). The difference however was not significant. In each group, one patient died from recurrent variceal bleeding. Encephalopathy occurred in 8 patients after SP and in 15 patients after PCS. The rate of chronic encephalopathy was significantly higher after PCS (40 %) than after SP (7 %, $p < 0.05$). One-, 2- and 3-year survival (Kaplan-Meier) were respectively 93 %, 81 % and 67 % after SP and 78 %, 66 % and 39 % after PCS. The difference between survival curves (Log-Rank) was significant ($p < 0.01$). These results suggest that, although it is slightly less efficient in preventing recurrent bleeding, the Sugiura procedure is better tolerated and associated with longer survival than portacaval shunt. The Sugiura procedure is an operation of choice when surgical prevention of recurrent variceal bleeding is contemplated.

FP032

**PORTAL PUMPING: A NEW PERSPECTIVE FOR TREATMENT OF VARICEAL HEMORRHAGE AND LIVER FAILURE IN END-STAGE CIRRHOSIS?**

Jorge E. CARDOSO, Mahmud EL METEINI, Yvon CALMUS, Michel VAUBOURDOLLE, Didier HOUSSIN.
Laboratory for Surgical Research and Department of Surgery, Cochin Hospital, Paris, France.

In end-stage cirrhosis complicated by variceal hemorrhage, treatments such as portosystemic shunts which aim to reduce portal pressure decrease sinusoidal perfusion at the same time, with the risk of impairment of liver function. A new concept was put forward that encouraging portal flow to pass through the cirrhotic liver by mechanical action could result in a decrease of distal (splanchnic) portal pressure on one side, and improvement of liver function on the other side. The aim of this work was to evaluate the hemodynamic and functional effects of a short term pump driven increase of portal blood flow through the liver of 13 patients with end-stage cirrhosis before the anhepatic phase of liver transplantation. In 10 of them, portal flow was increased peroperatively during 30 minutes, using a pump-driven venovenous bypass in a portoportal closed circulation. Basal portal flow ($800 \pm 270$ $ml.min^{-1}$) was increased two-fold (n=10) or four-fold (n=9). When flow was doubled, splanchnic portal pressure decreased 18% (from $31.8 \pm 5.7$ to $26.0 \pm 5.8$ mmHg, $p<0.001$); when flow was increased four fold, splanchnic portal pressure decreased $39.2 \pm 15.4\%$ (from $32.8 \pm 5.0$ to $19.9 \pm 6.0$ mmHg, $p<0.001$). Comparison of indocyanine green clearance between basal and doubled portal flow demonstrated an increase of $32.1 \pm 26.9\%$ (n=5; p=0.053). Histological analysis demonstrated sinusoidal dilatation in 3 out of 10 livers. According to these results, and with previous studies using isolated perfused cirrhotic rat or human livers, we suggest that portal pumping should be explored as a new perspective of treatment for some cirrhotic patients, sclerotherapy-resistant, with variceal hemorrhage and liver failure.

FP033

### A 19 YEAR EXPERIENCE WITH SELECTIVE SHUNTS FOR PORTAL HYPERTENSION

H Orozco, MA Mercado & J Hernández

Portal Hypertension Clinic
Instituto Nacional de la Nutrición

Selective shunts were described in 1967 and were initially performed in our hospital in 1973. Herein we report our experience with this kind of operation.
Material and Methods: In a 19 year period, 192 patients were operated in an elective function. In 170 cases, liver cirrhosis was demonstrated and in 85 alcohol was the etiology. Mean age was 50 (range 11-76). Most of them were low risk patients (132 Child A patients, 45 Child B and 15 Child C). The postoperative results, regarding operative mortality, re-bleeding, encephalopathy, and long term survival were analyzed.
Results: 140 distal splenorenal shunts were done and 52 splenocaval shunts (30 direct, 22 indirect). Operative mortality was 16% for the whole group, and 12% for the Child A group. Postoperative encephalopathy was 16%, but only 7% was incapacitating. Shunt thrombosis was demonstrated in 4% of the cases. Survival for the whole group (Kaplan-Meier) was 67%% at 1 year, and 56% at 5 years. Survival for Child A group was 75% at 1 year and 65% at 15 years. In 72% of the surviving patients, a good quality of life was recorded.

FP034

### NEW COMBINED OPERATION FOR LIVER CIRRHOSIS IN CHILD C GROUP PATIENTS

A V Shaposhnikov, S A Shaposhnikov

Department of Surgery, Rostov Medical Institute, Rostov-on-Don, Russia

34 patients with Child C grade liver cirrhosis underwent combined operation that included:

Marginal resection of 150-200g of liver tissue and intra-operative application of He-Ne laser radiation (670nm) for 10 min; ligation of splenic artery and omentohepatopexy; resection of $300cm^2$ of peritoneum on both sides of the abdominal wall - peritoneal-muscular shunt.

The main purpose of the first procedure was stimulation of liver regeneration; the second - decreasing portal hypertension. The last part of the operation was made for the reduction of ascitic liquid in the peritoneal cavity. The mortality rate at the first month after operation was 8.8%. Follow-up results were investigated for 1-5 years after operation (31 patients, group A) in comparison with non-operated patients with Child C liver cirrhosis (35 patients, group B). Number of survivors present in the table:

| | Years after operation | | | | |
|---|---|---|---|---|---|
| | 1 | 2 | 3 | 4 | 5 |
| Group A (31) | | | | | |
| N | 22 | 20 | 17 | 16 | 16 |
| % | 71.0 | 64.5 | 54.8 | 51.6 | 51.6 |
| Group B (35) | | | | | |
| N | 30 | 22 | 12 | 4 | 1 |
| % | 85.7 | 62.8 | 34.3 | 11.4 | 2.8 |

These results showed the effectiveness offered by the operation for patients with Child C liver cirrhosis. The operation can be useful in cases where it is impossible to perform shunting procedures or liver transplantation.

FP035

### INFLAMMATION IMPAIRS NEURALLY MEDIATED CONTRACTION OF GALLBLADDER MUSCLE

ML McKirdy, HC McKirdy, CD Johnson

University Surgical Unit Southampton
Ceneral Hospital, Southampton UK

It is known that patients with gallstones may have reduced contractility of the gallbladder. Evidence is accumulating that physiological gallbladder contraction is neurally mediated. The effect of inflammation in the gallbladder on its contractility is unknown. This study was designed to correlate response to neural stimulation with severity of inflammation in strips of human gallbladder muscle.

Strips of gallbladder muscle were suspended in an organ bath and attached to an isometric tensiometer. Electrical field stimulation (EFS) was applied by 5-10 second trains of electrical pulses (0.3 msec, 10Hz) to stimulate nerve cells. Response to a fixed low dose (15nM) of CCK octopeptide was measured by the increase over unstimulated tension after 10 minutes. Inflammation was assessed by a numerical score of nine architectural and cellular features graded absent, present or extensive.

41 gallbladder strips responded to EFS; 44 did not. In responders the median inflammation score was 7 (range 3-12) and in non-responders it was 11 (5-16; $p<0.001$). All gallbladders responded to CCK-OP. There was a significant ($p=0.0032$) inverse relationship between inflammation score and increasing tension.

The EFS stimulation characteristics selectively stimulate nerves. These results show that neurally mediated contraction in the gallbladder is likely to be lost in the presence of extensive inflammation. Contractility in response to CCK was reduced by increasing inflammation. These findings may be related to loss of nerve tissue or impaired muscle cell function in inflammation.

**FP036** **SPHINCTEROTOMY PRESERVING THE GALLBLADDER 'IN SITU': Effect on gallbladder physiology in an experimental model in the rabbit.**

**Pros I, Targarona EM, Martinez J, Balagué C, Casals E, Bassa P, Solà M, Trias M.**
**Service of General and Digestive Surgery, Biochemistry and Nuclear Medicine. Hospital Clinic. University of Barcelona, Barcelona, Spain.**

Endoscopic sphincterotomy leaving the gallbladder 'in situ' (EE-GiS) has emerged as an useful alternative to surgery, but little interest has been paid to gallbladder function after ES, and several studies report contradictories results. **AIM:** To study the modifications in gallbladder function after sphincterotomy in an experimental model of lithogenesis in the rabbit. **MATERIAL AND METHODS:** 38 male New Zeland rabbits were used. Group I, Control. Group II, Surgical sphincterotomy (SE). Group III, Gallbladder lithiasis induced by a lithogenic diet (colestanol 5%) during 2 weeks (Group IIIa) and 6 weeks (Group IIIb). Group IV. SE after induction of gallstones. Group V. SE previous to the induction of gallstones. A biliary scintigraphy with HIDA, weight of the gallstones, hepatic and gallbladder bile composition and hepatic blood function test (GGT, Alkaline Phosphatase (APh) an Bi) were measured. **RESULTS:** HIDA observed the gallbladder filling in all cases with a increased excretion fraction in the SE animals. In all the groups, gallstones were induced, but SE precluded the formation of gallstones in Group V. Dry weight of the stones was lower in the SE animals than in controls. Hepatic function blood test were in normal range in all cases.(Table I).

| Groups | % lithiasis induction | Stone weight (mg) | HIDA Eject.frac. | APh (ui/l) | GGT(ui/l) |
|---|---|---|---|---|---|
| Control | | | 57.6±15 % | 267±64 | 7.2±3.1 |
| Surrg Sphincterot. | | | 51.6±13 % | 160±36 | 7 ±2.8 |
| Galltosnes 2 w. | 100 % | 66.4±59mg | 72.5±6 %† †† | 232±60 | 6.7±1.1 |
| Litiasis 6s | 100 % | 276.5±319 mg | | 183±44 | 13.4±5.8 |
| Litiasis + EE | 100 % | 105.3±105 mg* | 86.8±5 %† | 158±36 | 7.6±3.1 |
| EE + litiasis | 66 % | 58.1±69 mg* | 91.8±3.6%†† | 113±47 | 6.8±1 |
| | *p=0.0007 | *p=0.02 | † ††p=0.01 | | |

SE decreased colesterol levels in gallbladder bile, and increase lecithin range in hepatic bile. **CONCLUSIONS:**1.Colestanol gallbaldder- stone induction is inhibited by SE. 2. After SE, gallbladder filling by isotope is preserved, but the empting of the gallbladder is increased, demonstrated by biliary scintigraphy with HIDA. 3. SE may inhibit lithogenesis because it facilitates a better empting of gallbladder.

FP037

### INTRAMURAL NEURAL PATHWAYS BETWEEN THE DUODENUM AND SPHINCTER OF ODDI IN THE AUSTRALIAN BRUSH-TAILED POSSUM IN VIVO.

J Toouli. JR Harvey, RA Baker, & GTP Saccone

Department of Surgery, Flinders Medical Centre, Bedford Park, South Australia 5042.

Balloon distension of the duodenum can provoke a change in sphincter of Oddi activity. The mechanism(s) which mediates such a response has not been defined. The aims of this study were to determine if: (i) electrical field stimulation of the duodenum influences sphincter of Oddi activity ; (ii) this response is neurally mediated ; (iii) these pathways are intramural and (iv) nicotinic and/or muscarinic receptors are involved. Twenty eight anaesthetized Australian Brush-tailed possums (Trichosurus vulpecula) were used. Electrical field stimulation [70V, 0.5ms, 5-60Hz, 10-20s] on the anterior serosal surface of the duodenum at 2, 3, 4, and 6 cm proximal or distal to the sphincter of Oddi, was used to stimulate neural pathways. The sphincter of Oddi phasic contractions were recorded by manometry. Tetrodotoxin (9µg/kg) was administered by close intraarterial injection to achieve neural blockade. Hexamethonium (30 mg/kg) and atropine (30 µg/kg) were administered i v. sphincter of Oddi activity was quantified by measuring the area under phasic contractions ($mm^2$ per 30 sec). The response was expressed as a % of the pre-stimulus activity.

All possums displayed spontaneous sphincter of Oddi phasic contractions. Electrical field stimulation of the proximal and distal duodenum produced excitatory sphincter of Oddi responses in all animals. These responses were frequency-dependant and maximal at 30Hz. The responses were produced when the duodenum proximal to the sphincter of Oddi (up to 4-5 cm, not at or beyond the pylorus ; n=4) or distal to the sphincter of Oddi (up to 4 cm; n=4), was stimulated. The responses were abolished by either pretreatment with tetrodotoxin (n=4), or transsection of the duodenum between the site of stimulation and the sphincter of Oddi (n=3). Hexamethonium did not significantly alter the sphincter of Oddi response to proximal (n=6) or distal (n=8) duodenal stimulation. Atropine reduced sphincter of Oddi response to proximal duodenal stimulation by 58.5 ± 8.5 % (n=6) and to distal duodenal stimulation by 34.0 ± 9.1 % (n=8) (both $P<0.03$, Wilcoxon Test).

In conclusion, we have demonstrated the existence of intramural neural pathways between the sphincter of Oddi and the segment of duodenum 4cm proximal and 4cm distal to the sphincter of Oddi. These postganglionic pathways involve muscarinic receptors.

FP038 BILE STASIS AFTER TRUNCAL VAGOTOMY

R Patankar, I Bailey, A Sanderson
C D Johnson

University Surgical Unit Southampton
General Hospital Southampton UK

The increased incidence of gallstones after truncal vagotomy has been attributed to bile stasis. CCK is known to be the principal hormonal mediator of gallbladder contraction. Our aim was to study the effect of truncal vagotomy on gallbladder contraction and CCK levels in response to a meal.

We studied 7 patients after truncal vagotomy and pyloroplasty and 13 normal subjects. After an overnight fast, gallbladder volume was measured in the fasting state and at 15,30, 45, 60 and 90 minutes after a solid fatty meal using the Ellipsoid formula (Dodds). CCK was measured by a specific radioimmuno assay at the same times.

TABLE

| | vagotomy median (range) | Normal Median (range) | |
|---|---|---|---|
| Baseline CCK pmol | (1.12(0.92-2.29) | 0.56(0.31-1.15) | p<0.01 |
| Maximum CCK pmol | 16.49(9.12-18.04) | 5.62(2.49-10.84) | p<0.01 |
| Integrated CCK | 621.07(307.7-846.0) | 300.5(160.9-642.9) | p<0.02 |
| Fasting volume | 17.55(12.27-31.33) | 13.52(7.7-40.31) | p<0.05 |
| Residual volume | 6.55(4.49-15.23) | 3.03(1.07-6.84) | p<0.01 |
| Ejection fraction | 63.4(51.38-68.2) | 78.29(61.3-90.67) | p<0.01 |

Gallbladder contraction was triphasic in both groups with a phase of relaxation separating two contraction phases.

Plasma CCK was significanty elevated after truncal vagotomy and may contribute to post vagotomy symptoms. There is decreased contractility of the gallblader with a lower ejection fraction and a higher residual volume after truncal vagotomy with consequent bile stasis. These findings suggest that reduced gall-bladder emptying after truncal vogotomy diminishes feedback inhibition of CCK release.

FP039

### EFFECT OF BILIRUBIN FREE RADICAL ON THE PRECIPITATION OF T-TUBE BILE

XS Zhou, DR Xiu, XB Fu, T Shen

Department of Surgery, Third Teaching Hospital, Beijing Medical University
Beijing 100083, China

We had reported at the 4th World Congress of HPB Surgery that free radical (FR) appears in gallstone in vivo, and that the presence of FR was essential in pigment gallstone (PS) formation. The AIM of this study is to explore the effect of FR on the precipitation of calcium bilirubinate (CaB) and glycoprotein (GP), the main ingredients of PS, from human bile in vitro.

FR intensity of bilirubin was enhanced by $^{60}Co$ radiation (BrE) or reduced by ascorbic acid and mannitol treatment (BrR). When this was confirmed by electron paramagnetic resonance spectra, 0.5 ml of BrE or BrR solution (5mg/ml, pH=8.4) was separately added into two 5 ml aliquots from each of 14 T-tube bile samples. After 36-hour incubation, the weight of dried precipitate, amount of GP and CaB in it, contents of GP, total bilirubin (TBr) and total calcium (TCa) in supernatant were measured and compared between paired aliquots (cf. table). Weight, CaB and GP of precipitate from BrE bile were significantly higher than those from BrR ($p<0.05$). The difference of components in supernatant (although $p>0.05$) matched the differences in precipitate.

| Indices | n | BrE(x±s) | Difference(x±s) | |
|---|---|---|---|---|
| Wt of prec (mg) | 9 | 22.8±8.3 | 3.01±3.86 | * |
| GP in prec (mg) | 13 | 0.109±0.063 | 0.024±0.031 | * |
| CaB in prec (mg) | 9 | 6.38±2.48 | 1.43±1.07 | ** |
| GP in sup (mg/ml) | 13 | 3.02±2.42 | -0.09±0.29 | |
| TCa in sup (μM) | 14 | 57.3±29.7 | -4.93±13.5 | |
| TBr in sup (mM) | 10 | 0.24±0.16 | -0.30±0.67 | |

Note: GP is expressed as bovine submaxillary mucin. Difference=BrE-BrR. Paired t-test, *:$p<0.05$, **:$p<0.01$.

CONCLUSION: FR might initiate the precipitation of CaB and GP, the first step of PS formation. (Supported by National Natural Science Foundation of China)

FP040 **BILIARY, PANCREATIC, BILIO-PANCREATIC REFLUX AND GASTRIC CANCER IN RAT.**

S Houry, P Bedossa, L Nguyen Thanh, M Huguier

Department of Surgery, Tenon Hospital
4rue de la Chine 75020, Paris.

Long term duodenogastric reflux has been implicated in the genesis of gastric stump carcinoma after previous partial gastrectomy for a benign disease.

The aim of this experimental study is to assess the effect of a permanent biliary (BR), pancreatic (PR), and bilio-pancreatic reflux (BPR) on gastric mucosa

After 2 months exposure, gastric mucosa did not display any macroscopical changes wathever the kind of reflux. On histological examination no intestinal metaplasia, hyperplasia or dysplasia were found. The only lesion found was the presence of numerous intra mucosal cysts nearly the anastomosis. The mean number of intra mucosal cysts was 119 for BR (n=5), 106 for PR (n=6), 60 for BPR (n=5) and 13 for control group (gastric suture n=5)

Long term exposure (one year) was investigated on BPR (n=9). Macroscopic examination showed a protruded lesion on the anastomosis of 8 rats. On histologic examination these tumours were in 6 cases an adenocarcinoma and in 2 cases a benign submucosal adenocystic proliferation. The six adenocarcinoma occured in five cases on an adeno-cystic proliferation, and in one case on an inflammatory granuloma.

Our results showed a clear corelation between long term exposure of BPR and gastric carcinoma in rats. Histologic investigations suggest that carcinoma arise through adenocystic proliferation, condtition similar to human gastritis cystic polyposa considered as a premalignant disease. This experimental model could be usefull for other investigastions.

FP041

### SCLEROSING CHOLANGITIS INDUCED BY SCOLICIDAL AGENTS INJECTED INTO THE BILIARY TREE OF RABBITS

Z Yılmaz, İ Pekrü, E Sözüer, A Kahya, A Yeşilkaya

Department of Surgery, Gevher Nesibe Hospital,
The University of Erciyes, Kayseri, Turkey

This study was performed at the University of Erciyes in Experimental Research Laboratory between November 1991 and August 1992; to investigate the effect of scolicidal agents, especially silver nitrate 0.5%, on liver and biliary ducts and to find out whether these agents caused secondary sclerosing cholangitis or not.
Three groups rabbits were taken in this study. Each group was included 15 rabbits. After laparotomy; The first group of rabbits was given sodium chloride 0.9% into biliary tract. The second group of rabbits was given silver nitrate 0.5%, and the third group of rabbits was given formaline 5%. Blood samples for SGOT, SGPT and ALP analises and liver wedge biopsies were taken. Five rabbits from each group were sacrified at the end of the first, fourth and eighth weeks. Liver, gall bladder and choledoc biopsies for histopathologic study were done. There was no histopathologic and biochemical change in the first group. The eighth week ALP values were significantly different from the beginning values in the formaline group ($p < 0.05$). The first and eighth week SGOT values and the eighth week ALP values were significantly different from the beginning values in the silver nitrate group ($p < 0.05$). In the both of formaline and silver nitrate groups, some of macroscopic findings and more of microscopic findings of secondary sclerosing cholangitis were found.

As a result; as formaline was found responsible for sclerosing cholangitis earlier, silver nitrate 0.5% was also found responsible for sclerosing cholangitis. Therefore, In the hydatid disease, any sclerosing scolicidal solution should not be given into the cyst cavity, till the solution must be proved not to be responsible for sclerosing cholangitis.

FP042 EXPERIMENTAL SCLEROSING CHOLANGITIS IN RAT INDUCED BY FORMALIN INJECTION IN THE BILIARY TRACT.

S Houry, P Bedossa, JP Benhamou, M Huguier.

Department of Surgery, Tenon Hospital
4 rue de la Chine 75020 Paris

Sclerosing cholangitis has been reported after surgical treatment of hydatid disease of the liver, and has been hypothetically attributed to the caustic effect of the scolicidal solution diffusing into the biliary tree trough a cystic-biliary fistula. The aim of this study was to assess the effects of scolisidal solutions on biliary epithelium.

In 146 rats we have injected directly into the biliary tract 0.15 ml of : isotonic saline (control group), 20% hypertonic saline, 0.5%, and 2% formalin.

Histologic investigations performed 3 months after injection showed no change in biliary structure in the control group (n=11). In hypertonic saline (n=14), and 0,5% formalin (n=12) groups, we observed very mild change of biliary epithelium, and no fibrosis. After 2 % formalin injection (n=16), there was periductal sclerosis in 11 rats and 4 of them developed pseudo-cirrhosis.

Sequential immunohistochimical study showed no change in control group (n=24). In 2 % formalin group (n=36), a mononuclear infiltrate inside and around the bile duct occured 7 days after injection and persisted until one year. At the later stages of the disease, Lymphocytes infiltrations were mainly T-cells. Ia antigene was expressed in biliary epithelium since the 7 th day.

Cholangiographic study showed normal aspect after hypertonic saline (n=11),and 0,5 % formalin (n=11). Strictures and dilatations of the biliary tree were observed in 10 rats after 2 % formalin injection (n=11). These strictures were confined to the ampula and the convergence in 4 rats, and located in the intra hepatic ducts in the others.

Our results suggest that 20% hypertonic saline and 0.5% formalin provide epithelium changes without sclerosis. 2% formalin provide sclerosing cholangitis Immunohisto- chimical study suggests a local cell mediated immune reponse in the pathogenesis of these lesions. 2 % formalin provide an experimental model of sclerosing cholangitis.

FP043 **THE PREVENTION OF FISTULAS WITH THE FIBRIN SEALANT IN PANCREATIC SURGERY: PERSPECTIVE RANDOMIZED TRIAL**

V. Costantino, A.Alfano D'Andrea, C. Longhini, C. Pasquali, S. Pedrazzoli.

Semeiotica Chirurgica, University of Padova, Italy.

Several Authors suggested the use of human fibrin sealants in pancreatic surgery to prevent fistulas. We performed a perspective randomized study including 97 patients who underwent resective or derivative surgery, 34 females and 63 males. 43 of them (Group A) had either the pancreatic anastomosis or the pancreatic resection sealed with fibrin glue during operation and 54 (Group B) had not. Twenty patients in group A and 26 patients in group B had an inflammatory pancreatic disease, while 23 patients in group A and 28 in group B had a pancreatic tumor (exocrine or endocrine). Sixteen patients in group A and 14 in group B underwent pancreaticoduodenectomy (PD); 16 (A) and 24 (B) had a pancratico- or cystojejunostomy (PJA); 10 (A) and 13 (B) had a left pancreatectomy (LP) and 1 (A) and 3 (B) underwent a tumor excision (TE). The sealant was made adding 500 U trombin to speed up solidification and either the anastomotic edges or the pancreatic sutures were sealed with spray. All the patients were treated and followed-up by the same surgical staff. The patients were randomized at the moment the surgical treatment was chosen, dividing the patients into 2 different lists going to have either a resective or a derivative operation. We considered only radiologically assessed fistulas. After surgery weobserved an overall number of 12 fistulas in 97 operations (12.7%). Six fistulas were found in group A (13.9%) and 6 in group B (11.1%). Five fistulas (16.1%) occurred in patients with pancreatic cancer (3 gr. A and 2 group B), 6 (13.0%) in patients with pancreatitis (3 in gr. A and 3 in group B), 1 occurred in a patient in group B with an endocrine tumor. According to the surgical procedure, we observed 5 fistulas (16.6%)in case of PD (4 in gr. A and 1 in gr. B), 5 (12.5%) after PJA(2 in gr.A and 3 in gr.B), in 1 patient in group B after LP and in 1 patient in group B after TE. Our results do not show any statistically significant difference between the patients treated with fibrin sealant and the control group. We point out that the highest incidence of fistulas was observed in the patients with pancreatic cancer in group A (18.7%) and in patients who underwent PD in group A (25.0%).

FP044

### VARIATIONS EXIST IN THE PROCOAGULANT ACTIVITIES OF HUMAN PANCREATIC CARCINOMAS

A K Kakkar[1]*, M F Scully[2], S Tebbut[1] and R C N Williamson[1].

[1] Department of Surgery, Royal Postgraduate Medical School, Hammersmith, London, W12 and [2] The Thrombosis Research Institute, Chelsea, London, SW3, UK

It has long been recognised that carcinoma of the pancreas is associated with a high incidence of thrombotic complications. Perhaps cancer cells generate procoagulant activity (PCA), which would not only predispose to thrombosis but also play an important role in tumour dissemination. Antithrombotic therapy at the time of implantation of certain experimental tumours will reduce metastatic potential. Since expression of tissue factor (TF) on the cell surface may activate local coagulation, expression of TF is an important determinant of PCA.

We studied the PCA of 4 cell lines derived from human pancreatic adenocarcinoma with normal and factor VII-deficient plasma to assess the role of TF in the generation of PCA.

| Cell Lines | PROCOAGULANT ACTIVITY (PCA) Thromboplastin Units/$10^4$ cells | | |
|---|---|---|---|
| | Total | TF dependent | TF independent |
| CaPanc2 | 6660 | 6560 | 100 |
| Pancl | 70 | 60 | 10 |
| BXPC3 | 3500 | 3450 | 50 |
| Mia Panc | 120 | 110 | 10 |

There is a great variation in the ability of human pancreatic carcinomas, of different origin, to generate thrombin, and this is particularly marked in TF dependent pathways. Should this ability correlate with biological behaviour, a new therapeutic pathway using specific antithrombins might become available.

FP045

### RESULTS OF PARTIAL RESECTION OF THE PORTAL VEIN OR SUPERIOR MESENTERIC VEIN IN PANCREATICODUODENECTOMY FOR CARCINOMA OF THE PANCREATIC HEAD REGION.

JH Allema, M Reinders, T v Gulik, DJ v Leeuwen, LTh de Wit, PCM Verbeek, MN vd Heyde, Department of Surgery, HPB-unit, Academic Medical Center, Amsterdam

The aim of this study was to evaluate the value of partial resection of the portal vein or superior mesenteric vein in patients undergoing pancreaticoduonectomy for carcinoma of the pancreactic head region.
Patients and methods: Between 1983 and 1992 196 patients underwent pancreaticoduodenectomy for carcinoma of the pancreatic head region. Partial resection and reconstruction of the portal vein or the superior mesenteric vein was performed (end to end anastomosis or venous graft) in 20 pts in which infiltration of the portal vein or superior mesenteric vein was found peroperatively, in combination with subtotal pancreaticoduodenectomy (11 pts) and total pancreatectomy (9 pts).
Results: In the group of patients with venous resection hospital mortality was 10% (2pts). Major complications were seen in 11 pts (55%): haemorrhage in 5 pts, intra-abdominal infections in 5 pts and mesenteric vein thrombosis in 1 pt. Relaparatomy was necessary in 5 pts (25%) and percutaneous drainage in 6 pts (30%).
In the group of patients with standard total pancreatectomy without venous resection (24 pts) hospital mortality was 16% (4 pts) and morbidity was 77% (19 pts). In the group of patients with subtotal pancreatectomy without venous resection (152 pts) hospital mortality was 5% and morbidity 40%.
In the group of patients with venous resection, pathology showed a pancreatic carcinoma in 11 pts, an ampullary carcinoma in 4 pts and a distal bile duct carcinoma in 5 pts. Only 3 pts had a radical resection (tumour free margins). One year survival was 26% and two year survival 11%, as compared to 69% and 50% resp. in the group of patients without venous resection. The tumour size and the differentiation grade were not significantly different from those in the group of patients without venous resection.
Conclusions: Partial resection of the portal vein or superior mesenteric vein in (sub)total pancreaticoduodenectomy for infiltrating carcinoma resulted in a low rate of radical resections and did not lead to improved survival.

FP046

### PLASMA GASTRIN AND CCK RESPONSE AFTER PYLORUS PRESERVING PANCREATODUODENECTOMY & DEFUNCTIONED ROUX LOOP PANCREATICOJEJUNOSTOMY.

L.J. Formela. A.N. Kingsnorth, *D. Chen,

Department of Surgery, University of Liverpool U.K. and *Department of Pharmacology, University of Lund S223, Lund, Sweden

There are several alternative methods to re-establishing continuity of the gastrointestinal tract following Billroth II conventional (B2PD) or Longmire-reconstructed pylorus preserving pancreatoduodenectomy (L-PPPD). B2PD abolishes the postprandial gastrin response whereas the response after L-PPPD is similar to controls. In the present study a novel reconstruction after PPPD has been designed to separate the biliary and pancreatic secretions by restoring continuity in the Billroth I manner with the pancreatic remnant anastomosed to a separate defunctioned Roux loop (D-PPPD).

Gastrin pmol/L and CCK pmol/L responses were measured in the fasting state and after a standard meal of 250ml 15% protein 30% fat 55% CHO with an energy value of 525KJ. Basal gastrin and CCK were similar ($p < 0.1$) in controls, B2PD and D-PPPD patients. In controls and D-PPPD but not in B2PD, postprandial gastrin rose at 10min from 8 ± 1.9 pmol/L to 13 ± 2.3 pmol/L, and from 7 ± 2.1 pmol/L to 15 ± 1.9 pmol/L, and remained elevated for 20 minutes. In the D-PPPD patients CCK levels remained low (0.2 ± 0.09 pmol/L) after the test meal. In controls postprandial CCK rose from 0.5 ± 0.1 pmol/L basal to 11 ± 2.1 pmol/L and returned to basal at 60 min. In B2PD post-prandial CCK rose at a similar rate and remained elevated for 80 min.

CCK is trophic to the pancreas. Abolition of the postprandial CCK response after D-PPPD removes this important potential stimulus for tumour regrowth in patients resected for cancer.

FP047

### PANCREATOGASTROSTOMY FOLLOWING PANCREATO-DUODENECTOMY : A SAFE DRAINAGE PROCEDURE?

JP Arnaud, C Casa, C Georgeac, J Ronceray, R Bergamaschi

C.H.U. ANGERS - Department of Visceral Surgery

4, rue Larrey 49033 ANGERS CEDEX 01 - FRANCE

The propensity for leakage and disruption at the site of the pancreaticojejunostomy is a major reason for morbidity and death after pancreaticoduodenal resection. The purpose of this study was to evaluate the role of pancreaticogastrostomy as an alternative method of restoring pancreaticointestinal continuity after pancreaticoduodenectomy.

From January 1989 to December 1992, 32 patients have undergone pancreaticogastrostomy after pancreaticoduodenectomy at our institution. Twenty two patients were men, and ten women. The mean age was 60.5 years (range 42-74 years). Pancreaticoduodenectomy was performed for pancreatic carcinoma (16 patients) ampullary carcinoma (10 patients), duodenal carcinoma (2 patients) and chronic pancreatitis (4 patients).

There was one postoperative death, for an overall operative mortality rate of 3%. There was one pancreatic fistula (3%) which recovered with further surgery. The average postoperative stay in the hospital was 14 days.

These results agree with data concerning pancreaticogastrostomy published in literature (1). Trypsin neutralisation by gastric acidity may explain the very low incidence of pancreatic fistula reported. In long term follow-up external pancreatic insufficiency does not seem to occur. Radiologic and endoscopic examination of the pancreatic duct is easy. This method of reconstruction merits widespread utilisation due to tis simplicity and safety.

(1) DELCORE R. AND AL : Pancreatogastrostomy, a safe drainage procedure after pancreatoduodenectomy.
Surgery 1990, 108 : 641-647

FP048

### THE PROBLEM OF DELAYED GASTRIC EMPTYING IN THE EARLY POSTOPERATIVE PERIOD AFTER PYLORUS PRESERVING PANCREATODUODENECTOMY (PPPD). Possible role of gastric motor dysfunction.

W.A.J.J.M. Haagh, L.M.A. Akkermans, H. Obertop.

Depts. of Surgery, University Hospital Utrecht, Utrecht, The Netherlands.

Delayed gastric emptying (DGE) has been reported as a frequent complication of PPPD, with an incidence of about 30 %. The cause of this phenomenon is probably multifactorial. We studied gastric myoelelectric activity, gastrointestinal symptoms and gastric emptying in the preoperative and early postoperative course in pancreatic and periampullary (PP) cancer patients to elucidate the possible role of gastric motor dysfuntion in the etiology of DGE in these patients.

Gastric emptying (GE) of a nutrient-rich liquid meal was studied in 22 preoperative PP cancer patients with no duodenal obstruction as evidenced by endoscopy or radiography (13 ♂, 9 ♀; mean age 61 ± 14 yrs) using Applied Potential Tomography, a technique measuring changes in resistivity in a thick cross-section through the abdomen at the level of the stomach. Myoelectric activity was measured concurrently by surface electrogastrography (EGG) during 1 hour fasting and 1½ hours postprandially and analyzed by computerized power spectrum analysis. Thirteen of these patients were also studied within the first 2 weeks following PPPD. Also symptoms of nausea, vomiting, regurgitation, early satiety and fullness were scored on a scale from 0 to 3 both preoperatively and postoperatively. The same measurements were done in 25 sex and age-matched healthy controls. Delayed gastric emptying was defined as $t½ > 180$ min, being the mean + 2 SD of the t½ in these normal subjects.

Gastric emptying was delayed in 7 preoperative PP cancer patients (32 %). There was a significant negative correlation between gastric emptying and pre -and postprandial mean gastric frequency in these patients. DGE occured in five of 13 postoperative patients after PPPD (38 %). The postprandial: fasting power ratio in the postoperative patients was significantly decreased compared to preoperative patients and controls (resp. 1.97; 4.92; 7.60, $p < 0.05$), indicating a decrease in gastric motor activity in these patients. Three of the postoperative patients with DGE also presented with DGE in the early postoperative period (60%). Also there was a clear association between postoperative DGE and the occurence of surgical (intraabdominal) complications. Gastric dysrhythmias were not observed in the pre -and postoperative period. No significant correlation could be demonstrated between DGE and symptoms.

Conclusions: 1) Preoperative DGE occurs rather frequently in PP cancer patients, which probably involves disease-related effects. 2) Gastric motor dysfunction, which may be pre-existent, can play a (minor) role in the occurence of DGE in the early postoperative period.

FP049

### SURGICAL SPLANCHNICECTOMY IN PALLIATIVE TREATMENT OF PANCREATIC CANCER

G. MANGIANTE, C. IACONO, G. PRATI,F. NIFOSI',
A.ISCHIA*,A.MOMBELLO **,N. CRACCO e G. SERIO
ISTITUTO DI PATOLOGIA CHIRURGICA
*3th RIANIMAZIONE OSPEDALE CIVILE
**ISTITUTO DI ANATOMIA PATOLOGICA
UNIVERSITY OF VERONA (ITALY )

In the treatment of pain from pancreatic neoplasms (NPP) discouraging results have been achieved by surgical splanchnicectomy.Nevertheless, these techniques complete a palliative surgical strategy of unresectable pancreatic cancer. The first step to achieve a satisfactory and long lasting relief of NPP is the correct anatomical identification of semilunar ganglions (SG) and splanchnic nerves(SN). In this light we tried to estimate the exact location,the number,the shape,and the lenght of SN and SG in 15 corpes (mean age 39,9 years- range 21-74,F/M=5/10 ). We also discuss which surgical approach to splanchnicectomy should be preferred in the presence of tumors located on the head ,or body and tail of the pancreas.

The main results are:

Right and left NS always pierce the diaphragm laterally the crus.On the right side SN always enter the abdomen posterior to inferior vena cava(IVC) .On the left side SN pierce the diaphgram strictly thickened to the left edge of the aorta(A) in the 66,6 % of the cases,close to the left edge in 26.6 %,and close to the right edge of left adrenal gland in 6.8 %. SN slide almost horizontally on the diaphragmatic bundles toward, and reach an area delimited by celiac trunk(CT) and superior mesenteric artery(SMA). The lenght of the right SN is 4.1 cm of mean(range 2 to 5.4 cm):the thickness is between 4 and 6 cm. The left SN is shorter (mean 2.4 cm- range 1.5-3 cm ).Right SG vary from 1 to 6 ganglionar bodies with various sizes from 5 mm to 3 cm; left SG vary from 1 to 4 ( sizes between 4 mm to 2.6 cm). On the left both SN and SG are completely covered by superior edge of the head and body of the pancreas and on the left side their detection is rendered easy after splenopancreatic mobilization. The shape of the ganglions is varies from a thick streak intermingled with swellings and single masses, rosary beads-like. The ganglions are always firm, pearl-coloured, easily separated from linfoglandular nodes. The postganglionar fibers spread from GS, like a shower, in the pancreas, and surround. In the chest, right and left SN get contributions of fibers from the sixth to the tenth thoracic ganglia.These fibers become a single bundle at a distance of 3.6 cm from the diaphragm,always strictly thickened to the prevertebral fascia.From May,1990 to June, 1992 we submitted 20 pts with unresectable pancreatic cancer to biliary-enteric by- pass plus bilateral splanchnicectomy. All the pts had complained severe upper abdominal pain.Postoperative mortality was nil: at 1-month postoperative follow-up 95 % of pts achieved complete pain relief.

Mean survival time was 7.9 months, and mean pain-free interval was 5.7 months.Pain recurrence was managed by percutaneous cervical cordotony (1),cordotomy+narcotic drugs(5), and narcotic drugs only( 12 ).Two pts died of progression of pancreatic cancer being rendered completely pain free. Two patient are alive without pain recurrence after 6 and 8 months respectively from the operation.

Palliative surgical procedure offers considerable benefit for the patients with unresectable pancretic cancer. Taking into account the above mentioned results we believe that neoplasms of pancreatic head are the most suitable conditions to perform a surgical splanchnicectomy by the right side through the hepatoduodenal legamentum,with a complete mobilization of IVC, whereas the retroduodenal-pancreatic approach seems to be more difficult. On the left the approach is particularly complex. The approach trough the i[illegible]rior edge of the pancreas, suitable for pancreatic body or tail neoplasms, is complicated by the presence of the tumoral mass, as reported in our surgical series. In presence of left neoplasms the transhiatale approach by Dubois is more suitable,in spite of its complexity.

Recurrence of pain was treated by combination therapy, such as percutaneous cervical cordotomy and/or narcotic drugs.

FP050 **ADJUVANT RADIO-CHEMOTHERAPY FOLLOWING RESECTION OF PANCREATIC ADENOCARCINOMA: EARLY RESULTS OF A PROSPECTIVE STUDY**

W Kmiot: on behalf of the UK
Pancreatic Cancer Trials Group

Dept of Surgery, Dudley Road Hospital
Birmingham, B18 7QU, UK

Resection for pancreatic cancer is associated with a 5 year actuarial rate (YASR) of 5-15%. Adjuvant therapy may be useful but there has only been one study published. The North American Gastrointestinal Tumour Study Group (GITSG) phase III study reported an improvement following post-operative radiotherapy and 5-FU i.v. weekly in 20 patients with pancreatic cancer (43% 2 YASR vs 18% in control arm).

In order to ascertain the value of the GITSG protocol, this was repeated by members of the UK Pancreatic Cancer Trials Group. 27 patients with adenocarcinoma of the pancreas (N=23) or periampullary adenocarcinoma (N=4; one with resected solitary liver metastasis) have so far been recruited. There were 16 men and 11 women with a mean age of 59.4 (range 40-76) years. A standard Kausch-Whipples operation was performed in 11 patients, 13 had a pylorus-preserving head resection, 2 had a total and 1 had a distal pancreatectomy. Lymph node metastases were present in 17 cases; the histological grade was 1=8, 2=11 and 3=8. The radiotherapy was well tolerated with only one related complication. There was no significant drug toxicity. At a median follow-up of 12 (range 3-36) months the 2 YASR was 30%; 14 patients were dead, 12 alive and 1 lost to follow-up.

These results indicate that post-operative radio-chemotherapy is well tolerated. The 2 YASR was intermediate between the treated and control arms of the GITSG study. A larger phase III trial may be necessary to establish the efficacy of the GITSG protocol, unless improved survival data are obtained with further follow-up.

FP051

### REQUIREMENT OF GAMMA LINOLENIC ACID IN HGF AND TGFβ INDUCED INHIBITION OF HEPATOMA CELL GROWTH

**WG Jiang and MCA Puntis**

**Department of Surgery, University of Wales College of Medicine, Cardiff, UK**

Gamma linolenic acid (GLA) is a cytotoxic agent for certain cancer cells. We have investigated the effects of GLA and its lithium salt (LiGLA) on hepatoma cell growth as regulated by hepatocyte growth factor (HGF) and transforming growth factor beta (TGFß).

The human hepatoma cell line, PLC/PRF/5, was cultured with or without cytokines, in the presence or absence of fatty acid (GLA and water soluble lithium salt GLA-LiGLA). The cell growth was quantified by a colorimetric method and shown as percentage growth compared with cytokine and FA free control. Statistics is Student T test and significant level was taken at $p<0.05$ (*).

| | culture medium | HGF(4ng/ml) | TGFß(5RU/ml) |
|---|---|---|---|
| Control | 100% | 96.7±1.7% | 85.2±2.1%* |
| With GLA | 86.5±2.8%* | 74.3±3.1%* | 45.4±1.9%* |
| With LiGLA | 87.9±2.7%* | 77.2±3.2%* | 70.0±1.2%* |

Both HGF and TGFß showed a concentration dependent inhibition of hepatoma cell growth and this is significantly enhanced by the presence of GLA and LiGLA. Lipid peroxidation account for part of the FA effects (tocopherol causes partial block of the effect). Other enzyme inhibitors (indomethacin and Nordihydroguaiaretic acid) have no effects. The morphological changes of the cells indicate that both necrosis and apoptosis are involved.

It is concluded that HGF and TGFß are both hepatoma growth inhibitors and GLA can enhance this effects, which may have clinical benefits.

FP052

### HUMAN KUPFFER CELLS ARE CYTOTOXIC AGAINST COLON TUMOR CELLS.

G Heuff[1], B Schuurman[1], R Beelen[2], S Meyer[1].

[1] Dept. of Surgery, [2] Dept. of Cell Biology, University Hospital and Faculty of Medicine, Free University, Amsterdam, the Netherlands.

Experimental animal models have demonstrated that Kupffer cells (KC) play an important role in controlling the growth of liver metastases. It has been speculated that human KC are also cytotoxic against tumor cells. By understanding the cytotoxic mechanisms of human KC, strategies to stimulate human KC can be outlined to prevent or eradicate hepatic metastases. However, so far no method has been available to isolate fresh human KC from liver biopsy material in large quantities to study supposed tumoricidal capacities of these cells.
This study describes a new procedure for isolation of KC from liver wedge biopsies (3-5g) without time consuming perfusion techniques. Liver tissue homogenate was incubated with pronase/DNAse for 45 min with continous pH registration and neutralization. The cell suspension was freed from erythrocytes, lymphocytes and cell debris by a nycodenz gradient and KC were finally enriched by counterflow centrifugal elutriation. Purity of KC was determined with the macrophage specific monoclonal antibodies CD68. Cell-mediated cytotoxicity of KC, with or without activation of gamma-interferon (IFN) was measured by an in vitro MTT-colorimetric assay against SW948 cells (a human colon tumor cell line).
From liver wedge biopsies $2x10^6$ KC per gram were harvested with a purity of > 95%. Maximum spontaneously cytotoxicity of non-activated KC was 50% at an effector to target ratio of 10. After activation of KC with IFN, cytotoxicity against the tumor cells increased to 85% (E/T 10).

In conclusion, this new method of KC isolation is a useful and simple method to isolate fresh human KC from liver wedge biopsies without perfusion. Moreover, it enables us to study antitumor capacities of these cells. KC were spontaneously cytotoxic against a colon carcinoma cell line. Furthermore, cell mediated cytotoxicity of KC increased after activation with IFN.

FP053

### REGRESSION OF AN ESTABLISHED MACROSCOPIC LIVER TUMOUR BY IN VIVO RETROVIRAL-MEDIATED TRANSFER OF A SUICIDE GENE

Y Panis, M Caruso, D Houssin, JL Salzmann, D Klatzmann

Laboratoire de Recherche Chirurgicale, Faculté de Médecine Cochin-Port-Royal and CNRS URA 1463, Hôpital La Pitié, Paris, France

Aim of the work : we investigated whether the direct intratumoral transduction of a suicide gene might induce the elimination of an established liver tumour. To specifically target the delivery and expression of the suicide gene into dividing cells, we used retroviral-mediated gene transfer and the herpes simplex virus type 1 thymidine kinase conditional toxin (HSV1-TK). Non toxic nucleoside analogs such as ganciclovir are metabolized by HSV1-TK into compounds that are toxic for dividing cells.

Animals and methods : In 25 BDIX rats, a single liver tumour was induced by injection of DHDK12 colon cancer cells under the liver capsule. At Day 5, tumour diameter ranged between 2 and 3 mm ; rats underwent an intratumoral injection of cells producing either HSV1-TK (treated group ; n=13) or (a nuclear $\beta$ galactosidase encoding marker gene) nls-lacZ (control group ; n=12) expressing recombinant retroviral particles. At Day 10, all the rats were given ganciclovir (150/mg/kg twice daily for 5 days). Autopsy was performed at Day 15.

Results : a dramatic reduction in tumour volume was noted in the treated compared to the control group : $8.1 \pm 6.7$ and $86.3 \pm 65.1$ $mm^3$, respectively, $p < 0.0001$). In the treated animals, the residual tumours were mostly made up of a massive fibrotic reaction with few or even no remaining tumoural cells.

Conclusion : Our study shows the regression of an established liver tumour after in vivo transfer of a suicide gene. This efficient therapeutical approach might be an ultimate treatment for disseminated liver metastases in humans, and could also be applied to the treatment of a large variety of solid tumours.

FP054

### HEPATOMA CELL GROWTH STIMULATED BY INTERLEUKIN-3

**WG Jiang and MCA Puntis**

**Department of Surgery, University of Wales College of Medicine, Cardiff, UK**

Interleukin-3 (IL3) is a haematopoietic growth factor and has been used following radical chemotherapy for solid tumours However there is a growing concern as to its possible stimulation of the malignant cells. We have investigated the effects of IL-3 on hepatoma cell growth.

A human hepatoma cell line, PLC/PRF/5, was used. Cells were cultured with recombinant human IL-3 at a wide range of concentration for up to 7 days and the cell growth is shown as mean (±SEM) percentage growth compared with control (culture medium only). Statistics is Student t test.

| | IL3(0.5ng/ml) | IL3(1ng/ml) | IL3(5ng/ml) | IL3(10ng/ml) |
|---|---|---|---|---|
| growth | 103.5±1.6% | 106.4±1.5% | 114.0±2.1% | 119.2±4.9% |
| p value | =0.12 | =0.01 | =0.001 | =0.01 |

IL-3 showed a significant concentration dependent stimulation of the cell growth and the stimulation was observed from day 2 to day 7. To determine the possible relationship between IL-3 and other cytokines on cell growth, a variety of other cytokines were tested. The stimulatory effect of IL-3 was significantly enhanced by IL-4, IL-6, and IL-10, but was completely reversed by IL-8 and TGFβ1. There was no significant effect on IL-3 induced cell growth by IL-1, IL-2, IL-5, IL-7, and TNFα.

It is concluded that IL-3 is a hepatoma cell (PLC/PRF/5) growth up-regulator and therefore an important factor in controlling hepatoma growth.

FP055

### HEPATIC IMMUNE FUNCTION IN THE RESISTANCE TO BACTERIAL TRANSLOCATION INDUCED BY INTRAPERITONEAL IMPLANTATION OF BIOMATERIALS

W Guo, R Andersson, XD Wang, S Bengmark
Department of Surgery, Lund University, Lund, Sweden

### Introduction:

The liver constitutes the majority of reticuloendothelial system (RES) function, providing a barrier to the passage of intestinal bacteria and toxins to the systemic circulation. Intraperitoneal biomaterial promotes bacterial translocation from the gastrointestinal tract. However, the role of hepatic immune function against bacterial translocation is not defined. The purpose of the study was to investigate the role of hepatic RES function in the resistance against bacterial translocation after intraperitoneal biomaterial implantation.

### Methods:

Rubber drains, knitted dacron and silicone elastomer with areas of 3 and 10 $cm^2$, respectively, were implanted into the right-lower part of the abdominal cavity of Sprague-Dawley rats under aseptic condition. Hepatic reticulo-endothelial function, expressed as the phagocytic index (k) and hepatic uptake of $^{125}$I-labelled *E. coli* was measured 1 day after implantation.

### Results:

A significant elevation of the phagocytic index (k) was noted in groups with intraperitoneal implantation as compared with sham operation. The hepatic uptake of $^{125}$I-labelled *E. coli* significantly increased after intraperitoneal implantation of different biomaterials. Translocation of bacteria from the gastrointestinal tract was also observed. The increase in phagocytic index/hepatic uptake of $^{125}$I-labelled *E. coli* and the incidence of bacterial translocation depended on the size of the biomaterial implanted. An inverse correlation existed between the hepatic uptake of $^{125}$I-labelled bacteria and the incidence of bacterial translocation to the liver.

### Conclusion:

Hepatic RES function increased after intraperitoneal implantation of biomaterials, reflecting a enhanced phagocytic function of Kupffer cells. It appears that the liver is an important part of host defense in preventing bacterial translocation induced by intraperitoneal implantation of biomaterials.

FP056

### REDUCED SPLANCHNIC BLOOD FLOW FOLLOWING SURGERY IN OBSTRUCTIVE JAUNDICE IS PREVENTED BY ENTERAL CHOLESTYRAMINE.

A Houdijk, P van Leeuwen, M Boermeester and R Wesdorp.

Department of Surgery, Free University Hospital,
Amsterdam, The Netherlands.

Surgery in patients with obstructive jaundice causes deleterious hemodynamic disturbances, possibly related to gut derived endotoxemia.
This study evaluates the effects of preoperative binding of gut endotoxin by cholestyramine on hemodynamics with special reference to splanchnic blood flow.
Male Wistar rats (n=20 per group, weight 250-300 g.) received the endotoxin binder cholestyramine (CHOL,150 mg/day) or normal saline (SAL) twice daily in the same volume. This treatment started on Day 1 and was continued until the end of the experiment. On Day 7 groups were randomized to receive a SHAM operation (SH) or bile duct ligation (BDL). This resulted in 4 groups (n=10 each) i.e. SH-SAL, SH-CHOL, BDL-SAL and BDL-CHOL. Subsequently rats were subjected to a surgical trauma by performing a xyphoidectomy on Day 20 and splanchnic organ blood flows were measured the following day using the radiolabeled microsphere technique.
A portion of the splanchnic organ blood flows is shown and expressed in $ml\,min^{-1}$ (mean ± SEM). Portal blood flow was computed by the sum of the flow to the individual splanchnic organs.

| Groups | SHAM (n=20) | BDL-SAL (n=10) | BDL-CHOL (n=10) |
|---|---|---|---|
| Portal venous flow | 16.32 ± 1.08[1] | 13.13 ± 1.12[6] | 15.95 ± 0.61 |
| Stomach | 0.92 ± 0.20[2] | 1.63 ± 0.19 | 1.80 ± 0.18[11] |
| Spleen | 1.63 ± 0.20 | 1.61 ± 0.16[7] | 2.10 ± 0.12[12] |
| Pancreas | 0.81 ± 0.14[3] | 0.50 ± 0.09[8] | 0.90 ± 0.09 |
| Small Intestine | 11.02 ± 0.90[4] | 7.73 ± 0.42[9] | 8.72 ± 0.40[13] |
| Colon | 2.20 ± 0.14[5] | 1.83 ± 0.13[10] | 2.36 ± 0.15 |

SHAM vs. BDL-SAL; 1 $p<0.05$, 2 $p<0.05$, 3 $p<0.05$, 4 $p<0.01$, 5 $p<0.05$. BDL-SAL vs. BDL-CHOL; 6 $p<0.05$, 7 $p<0.05$, 8 $p<0.001$, 9 $p<0.05$, 10 $p<0.05$. BDL-CHOL vs. SHAM; 11 $p<0.05$, 12 $p<0.05$, 13 $p<0.05$. Students T-test using Bonferroni correction.

Surgery in saline treated BDL rats resulted in a significant decrease in portal venous blood flow. This was due to a decrease in flow to the pancreas, small intestine and colon. Stomach flow was increased. Cholestyramine pretreatment prevented the decrease in portal venous flow by increasing blood flow through spleen, pancreas, small intestine and colon.
It is concluded that preoperative treatment with the endotoxin binder cholestyramine prevents the fall in splanchnic blood flow following surgery in the bile duct ligated rat.
Endotoxin is important in the development of postoperative complications in the bile duct ligated rat and the reduced splanchnic blood flow might enhance translocation of gut derived endotoxin.

FP057

### THE EFFECT OF PORTAL BRANCH LIGATION IN COMBINATION WITH OR WITHOUT DEARTERIALIZATION ON AN EXPERIMENTAL LIVER TUMOUR

B G Persson, L Q Wang, S Bengmark

Department of Surgery, Lund University, Lund, Sweden

Portal blood supply may play some role in ischemic treatment of experimental liver tumours. However, normal liver parenchyma can not tolerate 60 min of repeated total devascularizations (occlusion of artery and portal vein). In this experiment portal deviation (portocaval shunt) was replaced with ligation of the portal branch supporting the tumour-bearing lobe. Twenty seven rats were subjected to sham-treatment (n=6); portal branch ligation (PBL) (n=7); 120 min of repeat dearterializations (n=7) and portal branch ligation (PBL) combined with 50 min of repeat dearterializations (n=7). The results showed that portal branch ligation alone did not alter the tumour growth compared with sham-treatment ($p>0.05$). However, 120 min of repeat dearterializations effectively decreased tumour growth ($p<0.05$ vs all the other groups). There was no growth delay following portal branch ligation in combination with 50 min of repeat dearterializations ($p>0.05$). Significant atrophy of the corresponding lobe followed PBL regardless if it was combined with repeat dearterializations or not ($p<0.001$). Aminotransferases remained normal after PBL with repeat dearterializations for 50 min ($p>0.05$).

In conclusion, portal branch ligation did not retard tumour growth by its own, nor did it increase the antitumour effect following repeat dearterializations for 50 min. This study further confirmed that repeat dearterializations for 120 min alone is to delay tumour growth. It might have implications in clinical use of repeat dearterializations for the treatment of liver tumours.

FP058

### PHOSPHOLIPID DEPLETION OF LIVER TUMOURS AFTER A SINGLE OR REPEAT DEARTERIALIZATIONS

LQ Wang, BG Persson, N Xu*, S Bengmark

Department of Surgery and Internal Medicine*, Lund University, Lund, Sweden

Depletion of Phospholipids in ischemic cells is a causal event in ischemic injury. Dearterialization of hepatic tumours might induce phospholipid degradation in tumour cells as well. Phophatidylserine (PS) and phosphatidylinosital (PI) significantly decreased in tumours undergoing a single dearterialization for 2 hours compared with sham-treatment ($p < 0.01$). Phosphatidylcholine (PC) also decreased but without a significant difference compared with control ($p > 0.05$). The ratio of tumour/liver phospholipids was significantly reduced compared with control ($p < 0.01$) after a single dearterialization. Thus, dearterialization induced a fast degradation of tumour phospholipids without affecting the normal liver.

Repeat dearterializations for 2 hours during 5 days further reduced total tumour phospholipid. PC and PE were more significantly affected by repeat dearterializations ($p < 0.01$ and $p < 0.05$ vs control respectively). The ratio PC/LPC (lysophosphatidycholine) dropped remarkably ($p < 0.01$ versus control) indicating relative accumulation of LPC.

The decrease of phospholipid was consistent with the growth delay of tumour undergoing repeat dearterializations. Phospholipid degradation was paralleled by a delay in tumour growth that was significantly retarded by repeat dearterializations ($p < 0.01$ vs control). Further, ASAT and ALAT remained normal even after repeat dearterializations for 5 days, which suggested that repeat dearterializations was selectively delivered to tumour tissue sparing normal liver cells. In conclusion, repeat dearterializations selectively induced liver tumour ischemia and efficiently retarded tumour growth without any visible damage to normal liver parenchyma.

FP059

### DECREASING PROBLEMS WITH INCREASING EXPERIENCE. ANALYSIS OF 150 CASES OF LAPAROSCOPIC CHOLECYSTECTOMY

V Pegan, M Sever

Department of Gastroenterologic Surgery, University Medical Centre, Ljubljana, Slovenia

In the year 1992 we performed 151 laparoscopic cholecystectomies. After the optimistic start certain problems arose, all resulting from a lack of experience. Fortunately, all complications but one - a hepatic duct injury - were managed without major harm to the patient. Among our patients there were 120 women and 30 men, ranging in age from 13 to 82 years. The laparoscopic procedures were performed by four surgeons, alternating in the roles of the operator, the cameraman and the assistant. Conditions such as acute gallbladder infection and large stones, formerly constituting a strict contraindication, lost their significance with increasing number of cases. Among our first 50 patients there were six cases of conversion; later on no conversions were necessary.

The preoperative assessment, including the patient's history, laboratory parameters and high-quality US scans, was considered adequate, offering reasonable guarantee that no significant bile duct pathology would be overlooked. Intraoperative laparoscopic cholangiography was performed without problems in only four cases. The mean operative time was 65 minutes and the mean hospital stay 3.5 days; the patients returned to work in seven to ten days.

**Conclusions:** For the patient's benefit, surgeons are sacrificing their three-dimensional sight and tactile capabilities. A one-handed surgeon with only one eye left would still be able to perform a laparoscopic operation. In the hands of a skilled and experienced "laparosurgeon" the method is perfectly safe.

FP060 # BILE DUCT INJURY DURING LAPAROSCOPIC CHOLECYSTECTOMY

H T Khawaja, J A Rennie, E R Howard, I S Benjamin

Department of Surgery, King's College Hospital, London SE5 9RS, U.K.

Bile duct injury (BDI) during laparoscopic cholecystectomy (LC) appears to have a higher incidence than during open cholecystectomy (OC). We report our experience with 12 patients treated in our institution for BDI sustained during elective LC.

Three cases had been converted to OC due to technical difficulties and suspicion of BDI which was confirmed in two patients who underwent immediate hepaticojejunostomy (HJ). BDI was not substantiated in the other patient, who later developed a common hepatic duct stricture which required a HJ. The remaining 9 patients had no reported technical problems during LC but represented with BDI. Three patients had generalised peritonitis due to bile leak: 1 of these was referred for definitive treatment and had a HJ, but 2 initially underwent unsuccesful repair over a T-tube and ultimately had HJ. Six patients developed a common hepatic duct stricture (with a localised bile leak in 2). Five of these underwent successful HJ: the sixth was referred with a complex hilar stricture following an HJ and was treated by percutaneous dilatation. The median time from LC to the definitive surgical procedure was 18 weeks (range: 0 - 52). None of the patients had operative cholangiography and most BDI were not recognized immediately. The mechanisms of these injuries were not always clear at reoperation.

The inherent risk of BDI may be greater in LC than in OC, but this may be further increased by distortion of the anatomy, excessive use of diathermy or inexperience. The problem of training surgeons in laparoscopic procedures persists and should be urgently addressed.

FP061 PREOPERATIVE PREDICTIVE FACTORS OF BILE DUCT STONES IN PATIENTS UNDERGOING CHOLECYSTECTOMY.

A Gainant, P Bothorel, M Minani, P Cubertafond

Chirurgie A, C.H.R.U. Dupuytren, Limoges, France.

Routine intraoperative cholangiography for detection of common bile duct (CBD) stones in patients undergoing laparoscopic cholecystectomy is discussed, and many authors prefer selective cholangiography, performed only in patients with high risk of CBD stone.

The aim of this study was preoperative identification of patients with high risk of CBD stone.

From october 1990 to december 1992, 192 patients with symptomatic gallstones, without evidence of duct stone, underwent open or laparoscopic cholecystectomy. In all patients preoperative data included, clinical examination, research of previous history of jaundice or pancreatitis, liver biochemestry measurement, biliary tract ultrasonographic examination. All patients underwent operative cholangiography (O C).

147 patients had normal cholangiogram. 45 patients were found to have duct stone on O C. They underwent CBD exploration by choledocotomy . Stones were found in 43 patients. There was two false positive. Univariate analysis of preoperative findings between patients having or not CBD stones showed that nine variables were significantly more frequent in patients with CBD stones. They were : past history of jaundice, elevation of serum levels of total bilirubin, direct bilirubin, alanine amino transferase, aspartate amino transferase, alkalin phosphatases, amylase, and bile duct dilatation on sonography. Multivariate analysis showed that only four factors had independent significance in terms of predicting CBD stones : serum levels of alkalin phosphatases > 110 uI/ml, direct bilirubin > 8 µmol/l, bile duct diameter > 10 milimeter on sonography and history of jaundice. All patients with CBD stone had at least one of these factors. When one factor at least was present its sensibility in detection of CBD stones was 100 % and specificity 45 %. When three factors were associated sensibility was 92 % and specificity 79 %.

FP062

### CHOLESTEROL STONE RECURRENCE IN THE CYSTIC REMNANT AFTER LAPAROSCOPIC CHOLECYSTECTOMY

F Cetta, F Lombardo, M Giubbolini, A Cappelli, *A Pecchi, *A Cariati,

Inst. of Surgical Clinics and *Anestesiology, University of Siena and Inst. of Surgical Clinics, *University of Genoa, Italy

In a previous study[1], we have shown that: (i) cholesterol stones can recur after postcholecystectomy stones ; (ii) in patients with associated jaundice and pancreatitis, the removal of cholecystectomy and initially form in a long cystic remnant (LCR) (11 of 63 patients with the LCR determined the complete and persistent relief of symptoms). On the other hand: (i) the LCR itself rarely causes symptoms. Therefore, the excision of a LCR without associated stones will not eliminate postcholecystectomy symptoms in the great majority of patients; and (ii) the exact percentage of patients with LCR who are going to develop non brown gallstones many years after cholecystectomy is unpredictable, even if it is not negligible. Concerning stone recurrence, LCR can be defined as "a cavity lateral to the common duct, lined by gallbladder mucosa, acting as a mini-gallbladder, regardless of its length, ranging in our series from 0.8 to 4.5 cm". After the wide diffusion of laparoscopic cholecystectomy, which deliberately leaves a long cystic stump, it is presumed that also the number of postcholecystectomy stones related to LCR is going to increase, in the long term period. Since these stones are mostly cholesterol, possible prophylaxis using bile acid therapy could be indicated in selected cases. Therefore, a double blind trial with bile acid therapy was undertaken. Twelve patients with a cystic stump longer than 15 mm after laparoscopic cholecystectomy were enrolled. Patients with a presumed residual LCR were selected among those showing a particularly long and tortuous cystic duct at cholangiography. In particular, in 2 patients the cystic duct had a very low confluence, close to the ampulla of Vater. One of the last 2 patients plus 5 of the remaining 10 with LCR were treated by tauroursodeoxycholic acid (7 mg/Kg/die) to prevent cholesterol stone reformation. The remaining 6 patients didn't receive any treatment and were used as control. Ultrasound examination was scheduled every year and cholangiography every 3 years. At the moment, after a 6-month-follow-up, no patients in either group developed symptoms or gallstones.

*1) Cetta F et al Gastroenterology: 1992; 102: A306.*

FP063 **MANAGEMENT OF COMMON BILE DUCT STONES IN THE ERA OF LAPAROSCOPIC CHOLECYSTECTOMY**

G W W Gross, P Schauer, K R Sirinek

Departments of Medicine and Surgery, Audie L. Murphy Memorial VA Hospital & The University of TX Health Science Center, San Antonio, TX

Since the recent advent of laparoscopic cholecystectomy (LC), many have advocated a laparoscopic rather than an endoscopic (ERCP) approach to common bile duct stones (CBDS). This study reviewed the use of pre/post-op ERCP to manage CBDS during our initial experience with LC. Over an 18-month period, 450 patients underwent LC. Pre-LC ERCP was done if CBDS were suspected due to biochemical or sonographic findings, and intra-operative cholangiogram (IOC) was also obtained at LC in selected patients. Failure to achieve a cholangiogram or clear CBDS at ERCP usually resulted in open cholecystectomy (OC). 105 patients initially intended for LC (of whom 87 ultimately underwent LC) received pre-op ERCP; cholangiography was successful in 92, revealing CBDS in 35, with extraction achieved in 29 and endoscopic sphincterotomy (ES) performed in 34. IOC revealed CBDS in 2 patients with "clear" CBD at prior ERCP. During this period, 11 patients underwent post-LC ERCP due to suspicion of bile leak or retained CBDS (Table):

**TABLE: Post-LC ERCP Abnormalities in 11 patients**

| Findings | # Cases | Endoscopic Management |
|---|---|---|
| Bile duct leak only | 1 | Stent placement |
| Bile duct stricture only | 1 | Dilatation/Stent |
| Stricture + leak | 3 | ES all;stent 2/3 |
| Retained CBD stones | 2 | ES/extraction |

(The unstentable stricture required hepaticojejunostomy)

Conclusion: Employing our approach to CBDS, 4 patients (0.9%) suffered significant bile duct injuries (strictures), vs. an established 0.2-0.3% rate with simple OC. Since both open CBDE and LC are known to increase the rate of bile duct injury, we anticipated that laparoscopic CBDE/CBDS extraction would only further increase this risk. Therefore, we contend that, at the present level of technologic capability, widespread application of laparoscopic CBDS management is inadvisable and that ERCP should be the favored approach to this problem.

FP064

### COMPARISON OF LUNG FUNCTION IN PATIENTS AFTER LAPAROSCOPIC AND OPEN CHOLECYSTECTOMY

M. Krawczyk, W. Patkowski, K. Zieniewicz,

B. Najnigier, P. Nyckowski, M. Frączek

Dept. of General Surgery & Liver Diseases,

Medical Academy of Warsaw, Poland

The aim of the study was to compare the lung function in two groups of patients - after laparoscopic ( LCh ) and open cholecystectomy ( O Ch ).
From June 1991 to November 1992, 225 LCh and 250 OCh were performed. Two groups of 80 patients each, age and sex matched, were analyzed restrospectively, without randomization. The respiratory system sufficiency was evaluated with spirometry preoperatively and on 1st,7th and 14th day postoperatively. The following parameters were analyzed : volumetric - VC ( vital capacity ), functional - FVC ( vital capacity during forced full expiration ), $FEV_1$ ( fored expiratory volume in 1 sec. ), PEF ( peak expiratory flow rate ), PIF ( peak inspiratory flow rate ), % $FEV_1$ ( $FEV_1$/VC ), MEF ( mean expiratory flow 25 % ), MEF 50 ( mean expiratory flow 50 % ).
Results : All of functional tests on the 1st postoperative day were diminished - 12 - 16 % in LCh group and 38 - 46 % in OCh group. Spirometric values on 7th postoperative day were normal in LCh group, when in OCh patients - were diminished about 20 %. In later group, normalization of the functional test took place after 14 days. In LCh group neither atelectasis nor pneumonia were observed postoperatively, however they occured in 10 % of OCh patients.
We conclude, that laparoscopic cholecystectomy is the operation much less affecting the lung function, when compare to open cholecystectomy.

FP065 THE ACUTE PHASE RESPONSE AFTER LAPAROSCOPIC CHOLECYSTECTOMY AND AFTER OPEN CHOLECYSTECTOMY

L.Dominioni,S.Cuffari, G.Giudice, G.Carcano, L.Nicora, R.Dionigi.

Department of Surgery, University of Pavia-Varese,Ospedali Multizonale e "Del Ponte";Varese,Italy

The aim of this work was to assess the acute-phase alterations of blood lymphocyte subsets, plasma proteins and selected hormones in patients undergoing laparoscopic cholecystectomy (LC) and open cholecystectomy (OC), to compare the effects of surgical trauma resulting from the two different procedures.

METHODS. Fifty-seven well nourished patients candidates to cholecystectomy for uncomplicated cholelithiasis, without indication to common bile duct exploration, were prospectively randomized into two groups undergoing either LC (n=30) or OC (n=27), using the same type of general anesthesia. In each patient blood samples were drawn preoperatively and at various time intervals after surgery, to determine the concentration of lymphocyte subsets CD3, CD4, CD8, OKDR and the plasma levels of C-reactive protein (CRP), cortisol and prolactin.

RESULTS. The duration of operation and of general anesthesia was similar in the two patient groups. The postoperative hospital stay was shorter after LC than after OC (3.1(0.5) vs. 7.1(1.6) days; $P<0.001$). After OC a significantly greater ($P<0.05$) depression of blood CD3 and OKDR counts was found, as compared to LC. Moreover, OC patients showed a significantly greater postoperative acute phase increase of plasma CRP ($P<0.001$), of cortisol ($P<0.05$) and of prolactin blood levels ($P<0.001$).

CONCLUSIONS. The significantly lower acute phase responses of immunologic and metabolic parameters observed after LC support the concept that the laparoscopic procedure is less traumatic.

FP066

### Prospective hemodynamic study during per-coelioscopic cholecystectomy in 15 patients ASA III and ASA IV

D.Pezet[1], C.Ferrier[2], K.Slim[1], F.Ruiz[2], V.Tuber[2], L.Fourgeaud[2], P.Schoeffler[2], J.Chipponi[1].

1)Service de Chirurgie and 2)Dept. d'Anest. et Rea. BP 69
63003 Clermont Fd France

The postoperative benefits of percoelioscopic cholecystectomy (PCC) push the clinician to utilize this procedure for patients with bad general state of health .The aim of this study was to evaluate the hemodynamic consequences of PCC in patients(pts) staged III or IV for the American Society of Anesthesiology (ASA).

Fifteen pts mean age 70,5 +/- 10,3 years (41-87) were included in this study . Mean weight of 14 pts was 62,45 +/- 5,66 kg, 1 pt was obese (180 kg) . All patients have biliary symptoms . Three pts were staged ASA IV and 11 staged III . This staging was due to cardiovascular or pulmonary failure for 14 pts and for morbid obesity for 1 pt . During the procedure radial artery catheterisation provided the arterial pressure values (systolic, diastolic, mean) and blood samples for blood gases and pH measurement . Continuous capnography was made . Cardiac output, pulmonary artery and wedge pressure were provided by a Swan Ganz catheter witch also permitted the determination of systemic and pulmonary vascular resistances . In all pts anesthesia was induced using etomidate (0,3mg/kg) and fentanyl (7 microg./Kg) . Atracurium (0,3 mg/kg) provided myoresolution. Maintenance of anesthesia was realized with fentanyl,atracurium and 1,5 isoflurane in a mixture of nitrous oxyde and oxygen ($FiO_2$:0,5).This protocol was accepted by the ethical board .The pneumoperitoneum pressure was always inferior to 12 mm of Hg . In 8 cases there was a cholecystitis, in 6 cases the gallblader was not inflammatory and in 1 case there was an empyema of the gallbladder . No conversion in laparotomy was necessary .The mean duration of PCC was 76,3 +/- 24,4 mn (52- 150). There was no morbidity or mortality with a mean hospital stay of 6,2 +/- 3,5 days (4-12). Among all parameters registrated or calculated we analysed mean value of the 15 pts of : mean arterial pressure (MAP), cardiac index(CI) (Cardiac output / s.q.m.) and systemic arterial resistances(SAR) at time (T)1 after induction of anesthesia, T2 after insuflation of pneumoperitoneum, T3 immediatly after 15° proclitic position, T4 after 30 mn of 15° proclitic position and T5 after evacuation of pneumoperitoneum . Stastistical analysis was made by Barlett test .

Results are summarised in Table I

| | T1 | T2 | T3 | T4 | T5 |
|---|---|---|---|---|---|
| MAP mmHg | 88.6+/-24.4 | 97.6+/-16.2 | 92.2+/-31.7 | 95.9+/-22.5 | 95.9+/-14 |
| CI l/m | 1.78+/-0.45 | 1.87+/-0.38 | 2.28+/-0.83 | 2.23+/-0.68 | 2.98+/1.21 |
| SAR dyn.s/cm | 3886+/-1633 | 3619+/-681 | 3442+/-1281 | 2994+/-307 | 2705+/-970 |

During the procedure the Mean arterial pressure remained stable . A decrease in systemic arterial resistances was observed but not stastistically significant . The cardiac index was increased, reaching a significant difference at T5 ($p<0.003$) . In a few pts, poor hemodynamic conditions requiered special hemodynamic treatment during the procedure : Majoration of vasodilatation using nicardipine (3pts), nitroglycerine (2pts) and inotropic support using dopamine (2pts).

Percoelioscopic cholecystectomy is possible in ASA III and IV patients if the hemodynamic parameters are monitered (and corrected) during the procedure . Vasodilatators (ie isoflurane, nicardipine and/or nitroglycerine) have larger indications to maintain the cardiac index in these patients despite the adverse hemodynamic effect of pneumoperitoneum .

FP067 **TREATMENT OF HEPATOCELLULAR CARCINOMA BY ARTERIAL CHEMOEMBOLIZATION**

Marchiori L, Mansueto GC, Bortolasi L, Dal Dosso I, Colombari R, Bellini A, Nifosi F, Nicoli N.

PATOLOGIA CHIURGICA
POLICLINICO BORGO ROMA
37100 VERONA ITALIE

From 1989 to october 1992, 102 pts with hepatocellular (HCC) carcinoma were treated by chemoembolization (CHEM). Ninety-four pts had concomitant chronic liver disease. HCC was diagnosed by US, contrast enhanced CT, fine needle biopsy and alpha-feto-protein level. Admission criteria were as follow: tumor confined to the liver with or without hilar nodal involvement, Child class A or B, white blood count superior to 2.000 and platelets more than 75.000/mmc. All the patients underwent angiographic chemoembolization with Lipiodol and Mitomycin-C (42 pts) or DADH (60 pts). In 86 pts we performed transcatheter hepatic arterial embolization (TAE) which was not possible in 9 pts due to portal thrombosis and in 7 to technical cause. In TAE group 73 pts were Child A and 13 Child B. In 22 cases HCC was single nodular whereas in 64 was multinodular. In 51 pts tumor was superior to 5 cm in diameter (in multinodular HCC only the larger lesion was taken into account). In 35 pts diameter exceeded 5 cm. In 50 pts CHEM plus TAE was repeated. Seven pts died within one month after treatment: two for myocardial infarction, two for liver failure, two for digestive haemorrage and one for necrotyzing pancreatitis. Excluding deaths within one month, long-term survival rates were investigated in relation to various prognostic factors ( anti-cancer agent, number of nodes, tumor size, Child and Okuda stage, repeated procedures) using Kaplan-Meier method. In CHEM plus TAE group survival rate at 12 months is 47% and 35% at 24 months after one procedure, while is 90% at 12 months and 54% at 24 months after repeated procedure. CHEM plus TAE can be considered an effective measure, but survival is conditioned by the stage of cirrhosis and the tumor invasiveness and size.

FP068

### VALUE OF COLOR DOPPLER SONOGRAPHY IN DIAGNOSIS OF PRIMARY LIVER TUMORS

A Weimann, J Klempnauer, H Lang, P Lamesch, B Ringe, R Pichlmayr

Klinik für Abdominal- und Transplantationschirurgie, Medizinische Hochschule Hannover,Hannover,FRG

### Introduction

Several controversial reports are dealing with the value of Color Doppler sonography in the diagnosis of primary liver tumors according to the vascularization pattern.

### Patients and Methods

29 patients with various primary liver tumors were investigated preoperatively using a pulsed Color Doppler sonography unit SSA – 270 A (Toshiba, Tokyo, Japan). Final tumor diagnosis derived in all cases from histological examination of the resection specimen or in case of irresectability (n=5) from intraoperative biopsy.

### Results

Tumor hypervascularization including pulsatile signals at the periphery, in the centre of the tumor or both was observed in:
2 AFP negative of 4 cases with Hepatocellular carcinoma (HCC), 4 of 4 with Cholangiocellular carcinoma, 3 of 8 with giant hemangioma, 7 of 7 with Focal Nodular Hyperplasia, 3 of 4 with adenoma and furthermore in one neuroendocrine tumor. The tumor was hypovascular in 2 patients with HCC – one with status after chemoembolization and one with multiple intrahepatic tumor recurrence on underlying posthepatitic cirrhosis after liver transplantation 6 years before. Furthermore, the tumor was hypo– or even avascular in 5 patients with hemangioma and in one with adenomatous hyperplasia.

### Conclusion

With regard to the vascularization pattern, yield of Color Doppler sonography is low for differential diagnosis and prediction of tumor dignity. However, valuable information about tumor extension can be obtained in central tumor lesions close to hilar structures.

FP069

### ADJUVANT REGIONAL CHEMOTHERAPY WITH CISPLATIN AND LIPIODOL AFTER RESECTION OF HEPATOCELLULAR CARCINOMA.

G Borgonovo, N Bourokba, D Musset, S Maitre,
H Farah, C Vons, C Smadja, D Franco.

Service de Chirurgie et Service de Radiologie,
Hôpital Antoine Béclère, Clamart, France.

Early recurrence is frequent after surgical resection of hepatocellular carcinoma. The purpose of this work was to assess the value of postoperative adjuvant regional chemotherapy. Cisplatin (2 mg/kg bw) diluted in 10 ml of iodized oil (Lipiodol, Lab. Guerbet) was administered after selective catheterization of the hepatic artery above gastro-duodenal artery one month after resection and whenever possible 5 and 9 months postoperatively. Between April 1991 and December 1992, 24 patients had liver resection for treatment of a hepatocellular carcinoma. Thirteen patients were excluded from the study for one of the following reasons : residual cancer nodules after operation (4 patients), markedly impaired liver function or ascites (5 patients), spontaneous or surgical total portal diversion (2 patients), failure in selective catheterization of hepatic artery (2 patients). Eleven patients received the treatment : 6 patients had three courses of chemotherapy, two patients had 2 courses and three patients had one course. Side effects of the treatment were minimal and the mean hospital stay was 3 days. Two patients had prolonged epigastric pain due to gastritis. Recurrence was looked for in each patient by ultrasonography, CT scan and measurement of serum $\alpha$fetoprotein every 4 months. One patient died on the fourth postoperative month from spontaneous bacterial peritonitis. All other patients are alive. None of them demonstrated recurrence with a mean follow-up of 10.7 months (range : 4 to 18 months). Although our results are preliminary, they suggest that adjuvant regional chemotherapy using cisplatin mixed in iodized oil might be efficient in the prevention of early recurrence after resection of hepatocellular carcinoma.

FP070

### SURVIVAL AFTER LIVER RESECTION FOR HEPATOCELLULAR CARCINOMA ON CIRRHOSIS

G Gozzetti, A Mazziotti, GL Grazi, E Jovine, A Frena, M Morganti

2° Dpt. of Surgery, Univ. of Bologna, S. Orsola Hospital, Bologna, ITALY

Liver resection remains controversial for hepatocellular carcinoma (HCC) on cirrhosis, for the lack of liver regeneration and the risk of hepatic failure and tumoral recurrence. ***MATERIALS AND METHODS***: Over the past 9 years, 123 cirrhotics were operated on for single HCCs. An accurate work-up, including angiography with Lipiodol injection and CT scan, was adopted. Eighty-three pts. (67.4%) were in the Child-Pough's group *A*, 38 (30.8%) were Child B and the remaining 2 (1.6%) were Child C. Eight pts. (6.5%) had a right hepatectomy, 36 (29.2%) a wedge resection, while in the remaining 79 cases (64.2%) an anatomical segmental resection was performed. Intraoperative US was always employed. ***RESULTS*** Ten pts. died within 30 days, with an overall operative mortality of 8.1%; in the period 1987-1992 the mortality rate dropped to 5.4% (5/92 patients). Seventy patients (61.9%) are currently alive. Recurrence occurred in 38 pts. (33.6%). The 3 and 5 year actuarial survival rates for pts with follow-up > 30 days has been 60.1% and 36.3% respectively. Univariate analysis of factors influencing survival has shown better results for pts. 1) without symptoms; 2) with AFP level < 20 ng/dl; 3) who did not receive blood transfusion during surgery; 4) who had operation shorter then 3 hours; 5) without satellite nodules around the main tumor; 6) without microvascular thrombosis and 7) when the tumor was completely capsulated. On the other hand Cox's proportional hazards regression model has shown that only 1) the presence of a complete peritumoral capsule, 2) the smaller tumor diameter and 3) the absence of microvascular thrombosis were independent factors predicting statistically better survival (p<.01). The 3 and 5 year actuarial survival rates for the group of 21 patients with all these 3 variables have both been 79.1%. ***CONCLUSIONS*** Surgery can be proposed for cirrhotics with small, encapsulated HCCs and good hepatic function. Intraoperative US is an essential tool to avoid unsuccessful operations and to guide limited anatomical resections. Long term survival could be expected for selected patients.

FP071 FOCAL NODULAR HYPERPLASIA AND HEPATIC ADENOMA NEW EPIDEMIOLOGICAL AND DIAGNOSTIC FEATURES

D Cherqui, A Rahmouni, F Charlotte, H Boulahdour, JM Métreau
M Meignan, D Mathieu, ES Zafrani, PL Fagniez, D Dhumeaux

Hôpital Henri Mondor 94010 Créteil, France

Focal nodular hyperplasia (FNH) and hepatic adenoma (HA) are both benign liver-cell tumors occurring predominantly in young women. However, their pathogenesis and natural history are quite different. HA is usually a direct consequence of oral contraceptive (OC) intake and may lead to potentially lethal complications ; therefore it should preferably be resected. FNH is not induced by OCs and has virtually no complication ; therefore it may be observed. The aim of this study of 35 consecutive female patients is to report the present repartition of these lesions and the results of systematic appraisal of newer imaging modalities in order to avoid unnecessary liver resection.

From 1985 to 1992, 35 women were referred for supected benign liver tumor. Final histological diagnosis was obatined in all cases by tumor resection (n=32) or surgical biopsy (n=3). Imaging techniques included enhanced CT, enhanced MRI and more recently scintigraphy using TBIDA biliary tracer. CT and MRI were considered positive for FNH in the presence of a hypervascular lesion with a visible central stellate element and positive for HA in the presence of a capsule and tumor heterogenicity due to necrosis and/or hemorrhage. TBIDA scintigraphy was considered positive for FNH in case of decreased clearance of the tracer by the tumor resulting in a late hot spot.

Results are summarized in the table.

| | HA | FNH |
|---|---|---|
| number of patients | 4 | 31 |
| 1985-1988 | 3 | 5 |
| 1989-1992 | 1 | 26 |
| symptoms | 4/4 | 8/31 |
| OC intake | 3/4 | 28/31 |
| number of tumors | 4 | 47 |
| size (cm) | 5-15 (m=8,6) | 1-10 (m=4,5) |
| CT + | 2/4 | 25/45 (62%) |
| MRI + | 2/2 | 39/44 (82%) |
| TBIDA + | - | 24/27 (88%) |

Our results suggest : (1) a reduction in the incidence of HA, possibly due to the use of low-estrogen-content OCs in recent years, (b) an apparent increase in the diagnosis incidence of FNH, probably due to the diffusion of ultrasonography, (c) that a hypervascular tumor with a visible central element in an asymptomatic young woman is almost certainly a FNH and (d) that enhanced MRI and biliary scintigraphy are the best imaging techniques for the diagnosis of FNH.

FP072

### RESULTS OF LIVER RESECTION FOR HEPATOCELLULAR CARCINOMA IN PATIENTS WITH A NORMAL LIVER.

G Borgonovo, L Capussotti, C Vons, H Bouzari, C Smadja, D. Franco.

Services de Chirurgie, Hôpital Antoine Béclère, Clamart France, et Hôpital Mauriziano, Turin Italie.

Twenty percent of liver resections for hepatocellular carcinoma (HCC) are done in patients with a non cirrhotic liver. The purpose of this work was to assess the results of liver resection for HCC in 40 patients with a normal liver. Mean age (27 males and 13 females) was 56 years (range : 13 to 75 years). In 85 % of the patients there were symptoms : pain, fever, or weight loss. None of the patients had liver failure or portal hypertension. One patient had jaundice resulting from invasion of bile duct. There were 14 right, 10 extended right, 6 left and 2 extended left hepatectomies, and 8 segmental liver resections. One patient had resection of the bile duct. A vena caval neoplastic thrombus was removed in 1 patient. Liver resection was curative in 35 patients and palliative in 5. Mean tumor size was 123 $\pm$ 75 mm (range : 30 to 280 mm). The tumor was encapsulated in 58 % of patients. In 64 % of those, there was capsular invasion. Satellite nodules were present in 80 % and invasion of distal portal branches in 76 % of patients. There was no operative mortality. Five patients (12 %) had a major postoperative complication : intestinal obstruction in 1 and deep jaundice in 4, which subsided in 3. In the last patient jaundice persisted leading to major liver failure. On the 17th postoperative month this patient is awaiting liver transplantation. One-, two-, and three-year survival rates were respectively 80, 72, and 35 %. Tumor recurrence occurred in 28 patients (72 %) and was confined to the liver in only 13. These results suggest that the prognosis of HCC in patients with a non cirrhotic liver undergoing resection is approximately the same as that of patients with cirrhosis. The poor long-term survival might result from the marked spreading of the cancer within the liver due to late diagnosis in a population with no survey.

FP073

### OPERATIVE RISK OF MAJOR HEPATIC RESECTION

R Polastri, H Bouzari, M M Marucci, G Mattalia
L Capussotti

Department of Surgery, Ospedale Mauriziano
"Umberto I", Torino, Italy

Indications for liver resections have expanded with the extension of surgical limits for primary and secondary malignant liver tumours and increased diagnosis of benign liver tumours. We report our experience of major hepatic resections to evaluate the intra- and post-operative risk of this procedure.

Between October 1980 and November 1992, 322 hepatic resections were performed; of these, 129 were major hepatic resections. 69 patients were males and 60 females. The mean age was 57 years (range: 2-78). 19 patients had liver cirrhosis (Pugh A). 96 patients had malignant disease: hepatocellular carcinoma (n.39), cholangiocarcinoma (n.4), mixed primary liver cancer (n.4), hilar carcinoma (n.8), colorectal liver metastases (n.33), other liver secondaries (n.8); and 33 had a benign liver tumour.

Hepatectomies were defined according to the anatomic classification of Couinaud. There were 79 right hepatectomies, 22 extended right hepatectomies, 21 left hepatectomies, 4 extended left hepatectomies and 3 trisegmentectomies. In 45 cases we have performed other organ resections in addition to hepatic resection. From 1984 intraoperative ultrasonography was performed in all patients. In 22 we have avoided abdominal drainage.

There were 7(5.4%) intraoperative complications. In 2 instances of right hepatectomies extended to the segments IV and I we have injured the left hepatic duct. In all 2 cases they are sutured using a T tube protection. In 3 patients with large tumours adherent to the cava vein we caused a small injury of the latter: total hepatic vascular exclusion was necessary for 5 minutes in 2 patients. A partial improper closure of remnant left hepatic vein was made in the sixth patient, who had a right hepatectomy extended to the segment IV. An end-to-end suture was performed in total hepatic vascular exclusion. In the last patient, who had a right hepatectomy and a portal resection for hilar cancer, we had a torsion of an end-to-end portal anastomosis and we performed a reanastomosis. The median blood transfusion requirement was 2 units (range: 0-7); 37 patients did not require blood. The median operative time was 288 minutes (range: 150-600). Intraoperative complications were strictly related to the tumour size: 6 out of 7 patients had a tumour diameter of 10 to 30 cm. (average: 18.1 cm)

Two patients (1.5%) died 1 and 6 days after operation for myocardial infarction. 68 postoperative complications occurred in 48 patients (37.2%): pleural effusion (n.34), pneumonia (n.10), subphrenic collection (n.11), bile leak (n.4), wound infection (n.3), small bowel obstruction (n.2), pleural empyema (n.1), DIC (n.1) haemorrhagic gastritis (n.1), acute renal failure (n.1). Four patients (3.1%) underwent 6 reoperations: for drainage of subphrenic sepsis (n.3), small bowel obstruction (n.2), pancreatic fistula (n.1). In addition, 8 patients underwent percutaneous drainage of subphrenic abscesses.

The mean postoperative stay was 18 days (range: 6-102). Many pre- and intraoperative factors were analysed: only intraoperative blood transfusions correlated with a higher rate of postoperative complications and a longer postoperative hospital stay ($p < 0.01$).

FP074 PROGNOSTIC FACTORS IN SURVIVAL AFTER . PORTOSYSTEMIC SHUNTS.

G.P.Spina, R.Santambrogio, E.Opocher, A.Pisani Ceretti, M.Intra, P.Garancini *, G.Gallus *.

Ist.Scienze Biomediche S.Paolo-Univ. Milano
* Ist.Scientif.S. Raffaele - Univ. Milano

Multivariate survival analyses using Cox's Regression Model correlated long-term survival with clinical data from 239 cirrhotics with porto-systemic shunts for the prevention of variceal bleeding of the 12 years (from 1980 through 1992). There were 88 patients with alcoholic cirrhosis and 151 with non-alcoholic cirrhosis. The severity of liver disease was assessed according to the Pugh's classification (A=87; B=150; C=2) and to the hepatic score index (0.56± 0.08; range 0.38-0.81) calculated on 12 clinical laboratory parameters. (1) 127 patients underwent a total shunt and 112 a selective shunt. Mean follow-up was 57.4±38 months (range 12-142 m.). Five years survival was 59%. During follow-up, 81 patients died from their liver disease and 43 from various causes urelated to their liver disease. Five years survival curves showed a significant difference between hepatic score classes (<0.55=67%; 0.56-0.63=61%;>0.64= 36%; p<0.001), alcoholic and non-alcoholic cirrhosis (45% vs 66%; p<0.05) and alkaline phosphatase values (normal=63%; abnormal=49%; p<0.05). Ten preoperative variables (Pugh's score or hepatic score index, kind of shunt, portal thrombosis, portal perfusion, age, ethiology, alcohol abuse, platelets count, alkaline phosphatase and SGOT) were tested as a prognostic factors in a multivariate analysis using the Cox proportional hazard. In this highly selected popula - tion of cirrhotics, it showed that hepatic score index was the only variable significantly related to survival (B=5.04;SE=2.04;p=0.0136) These findings suggest that in this population of cirrhotics the severity of liver disease assessed by a hepatic score index (an enlargement of Pugh's classification) is the best predictive variable for long-term outcome after shunt surgery.

(1) G.P. Spina et al. Ann. Surg. 1990; 211: 178-186.

FP075 FACTORS AFFECTING INCIDENCE OF ENCEPHALOPATHY POST-PORTACAVAL SHUNT

P Schauer, G W W Gross, B A Levine,
and K R Sirinek

Department of Surgery
The University of Texas Health Science Center
San Antonio, Texas

Proponents of selective and small diameter (8mm), interposition-graft portosystemic shunts maintain that the incidence of encephalopathy post-total portosystemic shunt is secondary to loss of hepatic portal flow across a low pressure gradient anastomosis. This study assessed and correlated the incidence of post-op encephalopathy in 72 patients with alcoholic cirrhosis, portal hypertension, and variceal hemorrhage [Childs' class A (6%), B (40%), C (54%)] undergoing a side-to-side portacaval anastomosis (25mm) who had had portal hemodynamic studies pre-op, intra-op, and post-op. Sixteen patients (22%) developed clinically-evident <u>post-op</u> encephalopathy: 9/16 were not encephalopathic pre-op, 6/16 occurred early (<30 days), 10/16 were late (sepsis 4, dietary indiscretion 3, multiple and incapacitating 3), 4/16 died post-op, there was no difference in measured portal hemodynamic parameters in these 16 patients compared to 56 patients without encephalopathy (Table). Encephalopathic patients had worse hepatic function (Childs' class C 70% vs 46%) and higher incidence of emergency shunts (38% vs 9%). Encephalopathy occurs post large diameter portacaval anastomosis as a result of failure to acutely control gastroesophageal bleeding by nonoperative means in patients with poor hepatic reserve and is not secondary to the size of the anastomosis or the post-shunt pressure gradient across the anastomosis. A large diameter (25mm) side-to-side portacaval shunt with an 8% mortality, a 22% encephalopathy rate (new 12%, incapacitating 4%), and no recurrent variceal bleeding remains the "gold standard" for the treatment of variceal hemorrhage in patients with alcoholic cirrhosis.

| Pressures($mmH_2O$), X±SEM | Encephalop(-) | Encephalop(+) |
|---|---|---|
| Preop (WHVP) | 377±12 | 428±26 |
| Intra-op Portal Pressure | 191±7 | 201±15 |
| Intra-op Shunt Gradient | 15±2 | 18±5 |
| Post-op Portal Pressure | 174±7 | 208±13 |
| Post-op Shunt Gradient | 36±4 | 45±11 |

FP076

### STRUCTURAL CHANGES OF THE SMALL BOWEL MUCOSA AFTER PORTACAVAL SHUNT IN RATS

P Sharma, I Hägerstrand, F Bengtsson, B Jeppsson

Departments of Surgery, Pathology, Clinical Pharmacology, Lund University, Lund, Sweden

Intestinal absorption of different nutrients and toxic substances is altered after porto-systemic shunting. This alteration may play a role in the pathogenesis of hepatic encephalopathy. It is still controversial if porto-systemic shunting also is accompanied by structural changes of the small intestinal mucosa. We therefore studied small bowel mucosa after portacaval shunt in the rat.

Material and methods: Standard end-to-side portacaval shunt (n=29) and shamoperation (n=29) was performed under ether anesthesia. Four weeks after surgery rats were sacrificed and segments of proximal and distal small bowel was taken in a standardized fashion for light and electron microscopy.
The specimens were examined blindly. Villus height, villus to crypt ratio and villus size index were assessed on light microscopy. Microvillar height and microvillar count was analyzed by electronmicroscopy.

Results: No differences could be detected in villus to crypt ratio, villus size index and villus height between shunted rats and rats with sham operation. The vilus size index was high in the proximal small bowel in both groups and gradually decreased along the length of the intestine. On electron microscopy no differences could be detected in microvillar count per unit length of the brush border between shunted and sham rats. On the other hand microvilli of enterocytes from control rats were significantly taller compared to those in the shunted groups.

Conclusion: In contrast to other studies we have not found any changes of the small intestinal mucosa after shunting detectable at light microscopy, but a significant reduction of the height of microvilli on electron microscopy. The role of portal venous pressure in the regulation of mucosal absorptive surface by altering the height of microvilli needs to be studied further.

FP077

### SURGICAL TREATMENT OF BUDD-CHIARI SYNDROME COMPLICATED BY INFERIOR VENA CAVA OCCLUSION

B M Wang, B Y Ye, Y Tang, Q S Cao

Department of Surgery, Chang Hai Hospital
Shanghai 200433, PR of China

Decompression of the liver by either portacaval or mesocaval shunts for the Budd-Chiari syndrome complicated by inferior vena cava occlusion is impossible either due to hypertrophy of the caudate lobe or prone to failure due to high IVC pressure, as found during operation in our patients. The outcome of spleno-pneumopexy is also poor as indicated by 2 patients in this series (one referred for mesoatrial shunt due to failure of this procedure performed elsewhere). Six patients with adequate collateral circulation between the hepatic veins and IVC have had cavoatrial shunts. Seven patients with adequate collateral circulation of the occluded IVC have had mesoatrial shunts. One patient with severe venous hypertension of the lower extremities (varicosity, edema, pigmentation, and venous ulcer) had combined mesocaval and cavoatrial shunts. All of these shunts were constructed using a 14 or 16 mm ring - reinforced polytetrafluoro-ethylene graft.

Fourteen patients treated by shunts recovered well with resolution of ascites, diminution in liver size, and improvement in IVC pressure gradients. One patient complicated by post necrotic cirrhosis died of hepatorenal syndrome after a cavoatrial shunt. All shunts in the living patients have remained patent as determined by either Duplex scanning or contrast - enhanced CT scanning, during follow-up periods ranging from 3-48 months. Shunt operation may be the surgical treatment of choice in patients with Budd-Chiari syndrome associated with IVC occlusion.

FP078

### SHUNTING PROCEDURES IN BUDD-CHIARI SYNDROME : LONG-TERM RESULTS

MJ Boudet, G Zeitoun, JM Hay, Y Flamant.

Service de Chirurgie, Hôpital Louis Mourier
178, rue des Renouillers 92701 Colombes Cedex, France

The standard surgical treatment of Budd-Chiari syndrome (BCS) is still a shunt procedure between the portal and the caval system. Depending on whether or not the inferior vena cava is obstructed, the presence of a negative or weakly positive porto-inferior vena cava (IVC) gradient precludes a shunt bypass toward the IVC system. In that feature, a shunt bypass toward the superior vena cava system is needed.
Between 1/1973 and 12/1992, we performed 71 shunts on 62 patients. In 37 cases (34 patients), portal flow was shunted toward the IVC. In 34 cases (26 patients), the portal flow was shunted toward the SVC.
From 1/1981 to 12/1986, 17 patients with a BCS and an inferior vena caval obstruction were treated by an intrapericardial shunt (IPS) (i.e. mesoatrial shunt). From 1/83 to 12/92, 17 patients were treated by an extrapericardial shunt (EPS) avoiding opening the pericardium. Since 1/87, 14 of these patients were treated by two new procedures, namely a mesoinnominate (MI) or a meso-extrapericardial superior vena cava shunt.
All patients had pre and intraoperative liver biopsies (LB). LB were classified as follows : centrilobular necrosis (CLN) alone, CLN and severe fibrosis (F), F alone. There were : 16 CLN, 28 CLN and F, 27 F.12 patients had a preoperative acute renal failure (POARF) defined by a blood creatinine level > 120 µmol/l. Of these 12 patients, 9 had F.

**Results.**

Overall results :The overall mortality was 29 %. 9 of the 12 patients with POARF died (75%). 8 of these 9 patients had F on LB (90 %) ; 1 patient over 9 with POARF and F survived (10%). The two patients with POARF and no F survived. Statistical analysis showed the following results : POARF vs no POARF ($p<0.001$) ; F and POARF vs F and no POARF ($p<0.001$).
BCS with free IVC : Total early mortality was 27 % (9/33) : 1 intraoperative death, 4 septic shocks, 3 upper digestive bleedings, 1 pulmonary embolism. 4 of the 6 patients with POARF died. All had fibrosis on LB. 2 deaths occurred during the last 10 years and none during the last 5 years. Follow-up ranges from 1 to 15 years. One patient died 5 years later of unknown reason. Actuarial survival rate is 73% at 1 year, 73% at 5 years, 66% at 10 years.
BCS with IVC obstruction :IPS : 70% of the patients had postoperative ascites. Postoperative mortality was 57%. Of note, between the second and the fourth postoperative month, 4 patients (23%) had a pericarditis (2 acute and 2 constrictive) leading to a pericardial tamponade. 3 of them died even though a pericardectomy was performed.
EPS : Of the 14 patients treated since 1987, 1 patient died of a postoperative septic shock. Overall mortality was 7%. No pericarditis occurred.

**Conclusion**

Shunt procedures 1/ give excellent long-term results ; 2/ should be precluded when POAR and F are associated.
EPS should be preferred when IVCO obstruction is present.

FP079

### LONG-TERM RESULTS AFTER PORTAL SYSTEMIC SHUNTS IN BUDD-CHIARI SYNDROME.

C Vons, G Borgonovo, C Smadja, D Franco.

Service de Chirurgie, Hôpital Antoine Béclère, Clamart , France.

Budd-Chiari syndrome (BCS) occurs in patients with patent or latent myeloproliferative or coagulation disorders resulting in thrombosis of major hepatic veins. Portal-systemic shunts are indicated in order to use the portal vein as an outflow tract from the liver to systemic circulation. Late results are not yet known. The purpose of this work was to assess the results of portal systemic shunts in 15 patients with BCS operated on between 1978 and 1991. Twelve patients had a shunt with the inferior vena cava and 3 with the right atrium. The etiology of BCS was polycythemia rubra vera in 5 patients, oral contraceptive use in 4, pregnancy in 3, disseminated lupus erythematous in 1, and unknown in 2. There was no operative mortality. Twelve patients (80 %) were alive in december 1992 with a mean follow-up of 62 ± 30 months (range : 14 to 164 months). Living patients had a normal life with no ascites. The only biological abnormalities were a slight cholestasis. One patient had transient liver failure concurrent with hepatitis C infection. Three patients died 3, 18, and 84 monhs after shunting, respectively from end-stage lupus erythematous (1patient) and from blastic transformation of myeloproliferative disorder (2 patients). In one of the latter, blastic transformation resulted in hypercoagulability and in acute thrombosis of a previously patent mesocaval graft, 7 years postoperatively. This was the only patient with a thrombosed shunt during follow-up. These results suggest that the evolution of BCS patients after portal systemic shunt is favorable. Late complications and deaths result from the underlying systemic or hematologic diseases. Such results should be taken into account in the indications of shunts vs. transplantation in patients with BCS.

FP080

### THE SUGIURA-FUTAGAWA OPERATION FOR PORTAL HYPERTENSION. A 14 YEAR EXPERIENCE

H Orozco, MA Mercado, E Prado,
T Takahashi, G Rojas, R Zepeda,
S Rivera & K León.
Portal Hypertension Clinic,
Instituto Nacional de la Nutrición

The results in a 14 year period with the devascularizating operation described by Sugiura-Futagawa are reported here. The operation was done in patients in which a shunt was not possible to perform. One hundred sixty-one patients were operated in an elective fashion, all of them with different kind of hepatopathies (112 Child A, 44 Child B and 5 Child C). In 22 patients the procedure was done in one stage (13.6% operative mortality: 3 patients) and in 76 as a two stage procedure. 19 deaths were recorded in the 112 patients of the Child A group, with a total of 179 operations. The operative mortality for this group was 16.9% and related to the numbers of operative procedures 10.6% (19 of 179 operations). 38 patients were not considered for a second stage. Re-bleeding at long term was 6.8%. Incapacitating encephalopathy was found in 3 patients among the 111 survivors (2.7%). Survival (Kaplan-Meier) was 75% (1 year), 70% (5 years), and 69.2% (10 years). 10 esophageal fistulas were observed secondary to transection. The Sugiura-Futagawa operation is an excellent complement in the therapeutic armamentarium to treat portal hypertension, with a low re-bleeding and encephalopathy rate.

FP081 EXPERIENCES WITH IMPLANTATION OF DIFFERENT TYPE OF PERITONEOVENOUS SHUNTS

L Harsányi, P Kupcsulik, W Chanis, P Kokas

1st Surgical Department, Semmelweis Medical School, Budapest, Hungary

M+M: In the past 11 years 92 patients underwent a surgical procedure because of intractable ascites. Ethiology of the ascites was alcoholic cirrhosis in 83 cases, chronic aggressive hepatitis in 6 cases, and Budd-Chiary sy. in 3 more cases. 49 LeVeen-type and 43 Denver-type shunts were implanted.

Results: In the early postoperative periode was observed a good effect (rapid diminution of body weight, a significant elevation of endogenous creatinine clearance) in 71% of the cases. The elevated serum-aldosteron (1640±280 pg/ml) and renin (3.4±0.3 ng/ml) activity decreased and normalizing tendency of hormonal response to concentration-dilution was measured. The also increased atrial-natriuretic-factor level remainded elevated however. Early complications such as bleeding (4), thrombosis of the jugular vein (3), DIC (1), hepatic coma (2) and others (4) occured in 14 cases (15%). Late complications were 9 thrombosis, 3 intraabdominal occlusion of the shunt, 4 sepsis and 5 others (22%).

Conclusion: In conclusion peritoneovenous shunt implantation proved to be usefull for reduction of complains caused by intractable ascites with an acceptable rate of complications.

FP082

### ANATOMICALLY PRECISE LIVER SEGMENTECTOMY - RESULTS OF TWO EXPERIMENTAL TRIALS IN SHEEP

H Lang, A Junge*, H Sitter*, H J Klotter*

Dept. of Surgery, Hannover Medical School, Hannover, Germany

*Dept. of Surgery, Philipps-Universität Marburg, Germany

Liver segmental resection is supposed to provide oncological effectiveness and preservation of functioning parenchyma simultaneously which is important especially in patients with compromised hepatic function. However, there is still little evidence that segmentectomies can be performed anatomically precise. Therefore, in two experimental trials a new technique of liver segmental resection (SR) was evaluated with regard to anatomical precision and alterations of biochemical profile.

**Material and Methods:**

According to previous studies the sheep liver is most suitable for segmental resections. In trial I (3 groups of 10 sheep) either segment II,III or IV was removed. Resection (SR) was performed after intraoperative ultrasound guided localisation of the corresponding segmental portal branch, its ligation after transparenchymal dissection and staining of the segment by injection of methylenblue. In trial II (2 groups of 10 sheep) a traditional resection (TR) of segment III or a sham-operation (laparotomy including 200 ccm bloodloss) were performed. Dissection of liver tissue was carried out with an ultrasonic aspirator (Sonoca, Quickborn). Precision of segmentectomy was evaluated by corrosion preparation of the liver. Clinical profile was assessed by intraoperative bloodloss and changes of liver function tests (postop. day 1-5).

**Results:**

Overall, 36 corrosion preparation were technically sufficient for assessment. In SR anatomically precise resections could be achieved for segment II and III in 66% each and for segment IV in 50%. In TR only one resection was anatomically precise ($p<0.01$, $X^2$-test). Bloodloss for resection of segment III was nearly the same in SR (mean and SD: 148 ccm $\pm$ 61) and TR (140 ccm $\pm$ 77). Liver function tests did not differ significantly between SR and TR.

**Conclusion:**

The new technique proved to be a safe and reliable surgical procedure in anatomically precise liver segmentectomy which might have advantages in patients at increased perioperative risk.

FP083

### HOW CAN WE SHARE PORTAL BLOOD INFLOW IN AUXILIARY PARTIAL HETEROTOPIC LIVER TRANSPLANTATION WITHOUT PORTAL HYPERTENSION?

I Nagashima, L Bergman, R Schweizer

Transplant Service and Surgical Research Laboratory, Hartford Hospital, Hartford, CT, and University of Connecticut School of Medicine, Farmington, CT. USA

Auxiliary partial heterotopic liver transplantation (APHLT) could be an attractive treatment for fulminant hepatic failure in which recovery of host liver might be expected. We studied the functional relation between auxiliary heterotopic partial liver graft and host liver in the presence of artificially created portal hypertension in pigs.

16 pigs underwent hepatic artery ligation and APHLT, and were randomly allocated to three groups as follows; group A(n=5): no treatment of host portal vein, group B(n=6): banding of host portal vein to make the host portal pressure higher than that of graft by 2 mmHg, group C(n=5): banding of host portal vein to make the host portal pressure same as that of graft. Postoperative immunosupression was done with cyclosporin, azathioprine and steroids.

Basically, the host and graft portal pressure was 8.5+/-0.2 (mean+/-SEM) mmHg and 10.7+/-0.3mmHg respectively. All the pigs survived well until sacrifice on day 40 post-operatively. At autopsy, all in group A had necrotic and atrophied grafts (170+/-27g) with graft portal vein thrombosis, and well hypertrophied host livers (868+/-103g). All in group B had well hypertrophied grafts (480+/-57g), and necrotic and atrophied host liver (289+/-52g). Three of 5 in group C had well hypertrophied grafts (496g+/-37g) as well as host livers (313+/-21g), although the remaining two had the same results as group A. HIDA-scan and histological inspection showed good correlation between function and macroscopical finding at autopsy.

APHLT could be a good and acceptable treatment for temporary support in fulminanthepatic failure. However, it should be mandatory to band the host portal vein to make the host portal pressure same as that of graft in cases without portal hypertension.

FP084

### PHOSPHOLIPIDS PREVENT ENTERIC BACTERIAL TRANSLOCATION FOLLOWING SUBTOTAL LIVER RESECTION IN THE RAT

R Andersson, XD Wang, V Soltesz, W Wang, A Ar'Rajab, S Bengmark

Depts of Surgery and Clinical Microbiology, Lund University, Lund, Sweden

Bacterial infectious complications, including intraabdominal sepsis and bacteremia, following major liver resection can at least partly be attributed to translocation of enteric bacteria. Attempts to prevent or treat such infections by use of antibiotics may instead result in colonization and/or overgrowth of surviving microbes. In the present study, the effect of enteric administration of phospholipids (phosphatidylcholine and phosphatidylinositol) on the prevention of enteric bacterial translocation, induced by subtotal liver resection in the rat was evaluated.

90 % hepatectomy was performed in male Sprague-Dawley rats. The animals were allocated to receive saline, phosphatidylcholine or phosphatidylinositol prior to operation. Enteric bacterial translocation, intestinal mucosal mass and enterocyte protein content were determined.

The incidence of bacterial translocation significantly increased 2 and 4 h following 90 % hepatectomy, as compared with sham operated animals. Enteric administration of phospholipids, however, significantly reduced the incidence of bacterial translocation afte 90 % hepatectomy. Phospholipid treatment also prevented the otherwise occurring postoperative decrease in intestinal mucosal mass and enterocyte protein content.

Enteral administration of phospholipids thus seems to protect against translocation of enteric bacteria and prevent from the decrease in intestinal mucosal mass and enterocyte protein content following subtotal hepatectomy in the rat.

FP085

### THE ROLE OF LACTULOSE IN PREVENTION OF BACTERIAL TRANSLOCATION IN OBSTRUCTIVE JAUNDICE

C Ozaslan, A Turkcapar, S Bengisun, M Kesenci, A Toruner

General Surgery Department, IBN-I Sina Hospital, The University of Ankara, Ankara, Turkey

Despite improvement in operative techniques, better pre-operative evaluation and post operative care and uses of new antibiotics, mortality and morbidity due to infections and endotoxemia in obstructive jaundice still remain high.

In obstructive jaundice changes in bacterial flora occur because of absence of bile acids in gastro-intestinal (GI) tract and there is an increase in colonization of especially gram negative bacteria. Bacterial translocation (BT) may occur even though there is no damage to the intestinal wall.

In this study, effect of lactulose which decreases endotoxin levels and its absorption on BT was sought. Four groups of rats were studied; control (n:10), sham ligation (n:20), bile duct ligation (n:19), bile duct ligation and oral lactulose (n:20). In the control group during the first laparotomy specimens from mesenteric lymph node, liver and caecum were taken for culture. In the other groups the specimens were taken 14 days after the operation.

In the control group, no bacterial translocation was observed. In the sham ligation, bile duct ligation and bile duct ligation with lactulose administered, group BT rats were %5, %36 and %10 subsequently.

In the group whose bile ducts were ligated, there was an increase in E. coli and proteus colonization in the caecum. On the other hand in the lactulose administered group colonization of E. coli and proteus was found to be decreased.

Lactulose which decreases the endotoxemia exerts its effect by regulating the flora in the GT tract. The effect of lactulose on flora is through lowering pH in the caecum.

FP086

### INTESTINAL ENDOTOXINS AS CO-FACTORS OF LIVER INJURY IN OBSTRUCTIVE JAUNDICE

E Yılmaz, B Menteş, E Tatlıcıoğlu, Ö Uluoğlu

G Akyol, N Sultan

Depts. of Surgery, Pathology and Microbiology,

Gazi University, Faculty of Medicine,

Ankara, Turkey

The concept of endotoxin-mediated rather than direct liver injury in biliary obstruction was investigated using the experimental rat model of bile duct ligation (BDL) and small bowel bacterial overgrowth (SBBO). Small identical doses of intravenous endotoxin (LPS) were administered to sham operated rats as well as rats with BDL. LPS caused a significantly more severe liver injury in the BDL group, determined by the histologic score of the liver damage and serum gamma-glutamyl transferase, C-reactive protein and plasma LPS levels, suggesting the possible contribution of LPS in this type of liver damage within the framework of an altered Kupffer cell-macrophage system. After sensitization of the liver to minute amounts of LPS was documented, the possible role of intestinal endotoxins in precipitating the liver injury triggered by BDL was tested. In this respect, we surgically created jejunal self-filling blind loops (SFBL), which were known to result in SBBO and therefore increased intestinal endotoxin pool. When BDL was combined with SFBL, once again a profound liver injury was observed in contrast with the control group of rats with BDL+self-emptying blind loops (SEBL).

Small amounts of exogenous LPS and/or the ordinarily innocous amounts of LPS constantly absorbed from the intestinal tract may be critical in the hepatic damage caused by obstruction of the biliary tree. Therapy directed against intestinal endotoxin pool or LPS-mediated macrophage effectors may offer a new approach to modify or hamper liver injury in these cases.

FP087

### IMBALANCE BETWEEN TNFα & TNF INHIBITOR IN PATIENTS WITH OBSTRUCTIVE JAUNDICE

**MCA Puntis and WG Jiang**

**Department of Surgery, University of Wales College of Medicine, Cardiff, UK**

Tumour necrosis factor (TNF) is an important mediator in inflammation, but excessive production may cause host damage. TNF inhibitor (TNF-inh) is a soluble protein which block TNF actitivity. We have investigated the monocyte production of TNF and blood levels of TNF-inh in patients with obstructive jaundice.

29 jaundiced patients and 23 controls were studied. Monocyte TNF production (LPS stimulated) was measured by the L929 bioassay and shown as U/ml. TNF-inh was assayed using a TNF cytotoxic inhibition assay[1] and shown as percentage inhibition of TNF activity.

Monocytes from jaundiced patients showed greatly increased TNF levels and also increased TNF-inhibitor levels compared with controls. TNF/TNF-inh ratios, which reflect the balance of TNF and its inhibitor, are shown as follows:

| | Control | Jaundiced |
|---|---|---|
| All | 1.2±0.8 | 4.0±1.5* |
| Benign | 1.3±0.9 | 1.8±0.6 |
| Malignant | 0.4±0.3 | 5.1±2.1* |

* p<0.05 vs control, by Student t test

Five patients who died shortly after testing have the highest TNF levels but relatively low TNF-inhibitor, ratio=14.9±0.8, p<0.05 compared with the rest of jaundiced patients, ratio=2.2±0.7 and this suggests that patients who lack inhibitor protection have a poor immediate clinical outcome.

We conclude that jaundiced patients have greatly increased monocyte TNF production and also have increased levels of TNF inhibitor. The regulation of the balance between TNF and its inhibitor may be an important aspect to influence patients prognosis.

1. Baughman et al, J Lab Clin Med, 1991, 118:326

FP088 **THE HAEMODYNAMIC EFFECTS OF 8 ORNITHIN VASOPRESSIN IN PORTAL HYPERTENSION AND CIRRHOSIS**

**H. Kynaston, J. Yates, N. Davies, B. Taylor, K. Parsons, S.A. Jenkins**

**Department of Surgery, Royal Liverpool University Hospital, Liverpool, U.K.**

Previous clinical studies have demonstrated that 8 ornithin vasopressin (8-OV) improves renal function in patients with hepatorenal syndrome. The aim of this study was to investigate the effects of 8-OV infusion on systemic haemodynamics and organ blood flow in cirrhotic and portal hypertensive (PPVL) rats.

Two groups of PPVL and cirrhotic Wistar rats were studied (n = 20 and 12 respectively). Half the rats in each group received a 20 min infusion of 8-OR (0.043 U/hour) whilst the remainder received saline (control groups). Pulse, mean arterial pressure (MAP) and portal pressure (PP) were monitored continuously. Cardiac output (CO) and organ blood flow were measured prior to and after infusion by a dual microsphere technique. Intrarenal shunting was determined by a renal/pulmonary passing fraction method.

In PPVL rats 8-OV caused a significant fall ($p < 0.01$) in CO (mean difference + 95% CI = 33; 20,47ml/min), pulse rate (40; 16,65/min) and PP (4; 2.2,5.6 mmHg) and a rise in MAP (27; 20,33 mmHg). Absolute renal blood flow (ml/min/g) was maintained by a significant increase in %CO to the kidneys (mean increase = 8.7% = 5-12% CI). The cirrhotic group demonstrated similar significant changes in haemodynamics but neither control group exhibited any alterations. The cirrhotic group exhibited intrarenal blood flow shunting which was significantly reduced with 8-OV.

In conclusion 8-OV improves the hyperdynamic state found in PPVL and cirrhotic rats. Renal blood flow is preserved even though the cardiac output falls and intrarenal blood shunting in cirrhosis is reduced. These effects explain in part its beneficial effect on renal function in the hepatorenal syndrome.

FP089 **DETECTION OF LIVER METASTASES FROM COLORECTAL CARCINOMA: VALUE OF IMAGING TECHNIQUES.**

B van Ooijen, M Oudkerk, PIM Schmitz, T Wiggers.

Departments of Surgical Oncology, Radiology and Statistics, Dr Daniel den Hoed Cancer Center, ROTTERDAM, The Netherlands.

One of the most important prognostic factors determining survival among patients undergoing hepatic resection for colorectal metastases are the number of metastatic deposits in the liver. Since a number of more than 3 metastases may exclude a patient from resection, precise evaluation is essential to prevent needless exploration.
A prospective evaluation of the liver by preoperative ultrasound (US), conventional computed tomography (CT), and continous CT angiography (CCTA) was performed in 60 patients with and without metastases. All patients had a history of colorectal carcinoma and all underwent laparotomy. The standard of reference were the findings at laparotomy: palpation of the liver and intraoperative ultrasonography (IOUS). The imaging techniques were assessed independently of each other.
One hundred and five liver metastases were identified in 37 patients; 42 metastases were less than 1 cm in diameter. Twenty-three patients had no metastases. CCTA had a high sensitivity of 94% (99 lesions identified), in contrast to US (48%, 50 lesions identified) and conventional CT (52%, 55 lesions identified). The superiority of CCTA is also manifest in lesions under 1 cm in diameter. However, the high sensitivity is accompanied by a high-false positive rate particularly due to variations in the perfusion of normal liver parenchyma. Overall, CCTA had the highest accuracy (74%) compared to US and CT (both 57%).
The data indicate that preoperative US and conventional CT have low sensitivity in the detection of liver metastases and that CCTA can supplement information, although the low specificity of the technique makes its application difficult. Possibly, experience with image interpretation may allow the interpreter to accurately predict the likelihood that a specific lesion represent metastatic disease. In addition, combination with other imaging techniques may increase the accuracy.
**Conclusion:** Although CCTA seems to be superior to other preoperative imaging techniques the too low specificity will hamper its routine application in patients with hepatic metastases from colorectal carcinoma.

FP090

### INTRAPORTAL INFUSION OF 5-FLUOROURACIL AND LEUCOVORIN STARTED IMMEDIATELY AFTER HEPATIC RESECTION FOR COLORECTAL METASTASES. A FEASIBILITY STUDY.

R Doci, P Bignami, F Montalto, M Cataldi, L.Gennari

National Cancer Institute, Milan

Out of 208 patients submitted to hepatic resection for colorectal metastases, 121 developed a relapse that in 43% was localized in the liver and that in a further 17% was combined with extrahepatic sites. Hepatic recurrence may be due to subclinical metastases in the residual liver still present at the time of resection or to microembolia mobilized by surgical manoeuvres. It is not known either if the best form of "adjuvant" chemotherapy after hepatic resection is systemic or regional and, in this case, if through the portal vein, the hepatic artery or both. Experimental studies have shown that portal vein infusion can be effective on the hepatic micrometastases if the drug is delivered immediately after cells inoculation. According to this rationale a pilot study on feasibility of portal infusion immediately after hepatic resection was undertaken. In 10 unselected patients a portal catheter intraoperatively was positioned. Within 12 hours a continous infusion of 5-Fluorouracil (5-FU) 750 mg/m2/day plus Leucovorin (LV) 30 mg/day per 14 days was started. No technical complications nor hepatic toxicity were observed. One patient, previously treated with systemic chemotherapy, developed bone marrow depression and mucositis. Biochemical profile during postoperative treatment of these patients was compared with that of 28 patients resected during the same period. No differencies were observed in the two groups according to parameters of hepatic systhesis and necrosis. This preliminary report states that portal infusion of 5FU and LV started immediately after hepatic resection can be delivered safely. The accrual of patients in this study and their follow-up are in progress; it is possible that within 6 months data on recurrence rate and survival will be available.

FP091 RADIOIMMUNOGUIDED SURGERY: PROBE-ASSISTED LIVER RESECTIONS FOR METASTASES FROM COLORECTAL CARCINOMA

A.Benevento, R.Galozzi, S.Cuffari, L.Festi, G.Carcano, R.Dionigi.
Department of Surgery, University of Pavia-Varese, Ospedale Multizonale di Varese, Italy

The use of radiolabelled monoclonal antibodies to tumor-associated antigens in tumour diagnosis and detection is worldwide accepted. Recently a new tecnique has been proposed, based on the development of a hand-held gamma detecting probe for intraoperative use. We report our experience using radioimmunoguided surgery (RIGS) for resection of liver metastases from colorectal carcinoma. Since 1989, 54 patients with primary or recurrent colorectal carcinoma received preoperative intravenous injection of MAb B72.3, a murine IgG-1 that reacts with the high molecular weight glycoprotein antigen TAG 72 associated to colorectal cancer. It was radiolabelled with I-125. A total of 24 patients with liver metastases (13 synchronous and 11 metachronous) were considered eligible for resection at preoperative diagnosis. At surgery the whole abdominal cavity and its viscuses were explored by the probe, in order to confirm lesions preoperatively assessed and detect possible occult tumour deposits. Intraoperative ultrasonografic scans were routinely performed and compared. Probe assisted liver resection permitted to locate accurately liver metastases, to exclude lymphnode involvement, to delineate margins before resection, to control margins during non anatomical resection and to verify clear margins after resection. Five of 24 patients (19%) were found to have a large bilateral metastatic spread not previously detected; major procedures were avoided and a catheter for locoregional chemotherapy was placed in the hepatic artery via the gastroduodenal artery. Thirteen of 20 (65%) synchronous metastases and 20 of 25 (80%) metachronous metastases were correctly localized by the probe. Twelve metastases (7 synchronous and 5 metachronous) were unrecognized. Factors influencing MAb binding to colorectal cancer cells (MAb pharmacokinetics and physiological factors within the tumor) negatively affect the radiolabelling of tumour metastases. The smaller lesion identified was 5 mm in diameter. Compared to intraoperative US-scan RIGS shows a lower sensitivity (75%) and a higher specificity (100%). In 6 of 54 patients US-scan diagnosed suspicious or benign lesions (2 angiomas, 1 serosal cyst, 3 isoechogenic lesions). Correct diagnosis of metastases was allowed by RIGS. New highly selective MAbs, fragments of MAbs and human MAbs against different cancer cell lines would improve availability of the radioimmunoguided surgery technique.

FP092

### Determinants of the Natural History of Colorectal Liver Metastases

R. Stangl, A. Altendorf-Hoffmann, C. Schmidt,
D. Mowbray, O. Wiesinger, J. Scheele
Surgical Department,
University Hospital Erlangen, FRG

Between Jan. 1, 1980 and Dec. 31, 1990 data from 1099 patients with colorectal liver metastases were recorded at the University Hospital Erlangen. Excluding all patients, who received any kind of treatment, 489 patients remained for the univariate (log-rank) and multivariate (BMDP 2L) analysis of the determinants of the natural history of colorectal liver metastases. All patients were followed up to June 1, 1992 or death. At the closing date of the study only 11 patients were still alive.
**Univariate analysis** yielded the following highly significant factors: mesenteric lymph node involvement (MLNA), grading of the primary tumor, treatment of the primary tumor, radicality at the primary tumor site, hepatomegaly, percent liver volume replaced by tumor (%-LVRT), number of liver metastases, distribution of liver metastases, alkaline phosphatase, LDH, WBC-counting, CEA, Karnofsky-index and extrahepatic tumor. With **multivariate stepwise regression analysis** (Cox) 6 independent significant factors were selected: %-LVRT, grading of the primary tumor, MLNA, extrahepatic tumor and diameter of metastases. Subsequent combination of the multivariate significant factors resulted in a factor adapted prognostic assesment (FAPA). This prognostic "tree" demonstrates the heterogenity of this patient sample with median survival times ranging from 3.1 months to 20.3 months, dependent on the presence or absence of the multivariate, relevant factors.
Because of the extreme differences between the group consisting of less than 25%-LVRT and the group with more than 25%-LVRT we also carried out a multivariate stepwise regression analysis, selectively for each of these groups. For patients with ≤ 25% LVRT mesenteric lymph node involvement was replaced by LDH as a significant variable allowing a more adequate definition of prognosis. In patients with > 25 %-LVRT only grading of the primary tumor and diameter of metastases were proved independently significant.
In view of the lack of detailed knowledge on the natural history of colorectal liver metastases the prognostic "tree" presented may improve the assesment of palliative therapeutic approaches, with respect to prognosis compared to the natural history of colorectal liver metastases.

FP093

### A PILOT STUDY OF PERCUTANEOUS INTRALESIONAL LASER HYPERTHERMIA (ILH) FOR HEPATIC MALIGNANCIES.

I McInnes*, C Christophi, P Jones, C O'Donnell, M. Scelwyn, F Dudley.
Departments of Surgery, Gastroenterology, Radiology & Pathology, Alfred Hospital, Commercial Road, Victoria, 3181, AUSTRALIA

The **AIM** of this study was to prospectively evaluate the clinical, radiological and histological effects of ILH in a series of patients with primary and secondary hepatic malignancies. Seven women and 6 men, median age 56 (range 39-77) were referred for ILH. Nine patients had colorectal liver metastases, 2 had primary liver cell carcinoma with cirrhosis, 1 had metastatic haemangiopericytoma and 1 had sarcoma. The hepatic lesions were characterised at entry by CT scanning and ultrasound and the size and number of intrahepatic lesions noted.

**METHODS** The patients were considered suitable for treatment if their lesion(s) was >5cm and <15cms in size, <10 in number and accessible to percutaneous puncture. A 19 gauge needle was inserted into the centre of the tumour under ultrasound control, a naked laser fibre was then inserted into the core and the needle withdrawn. Laser therapy was then administered by continuous firing until the lesion became echogenic. Patients were assessed clinically and by ultrasound imaging during and for the first 24 hours after therapy and again at 2 weeks. CT was performed at 8 and 24 weeks.

**RESULTS** A mean 5000 Watts (range 1449-9449) dose was administered per session and each lesion received between 1 and 3 treatments (mean 1.8). Median follow up was 31 weeks (range 7-46). There were 2 deaths during this time and 1 patient refused follow up. Six patients had completed 6 month follow up, in 3 the size of the tumour(s) decreased but 1 developed extrahepatic metastatic disease and in the other 3 progression of local disease was apparent. Two specimens (1 post mortem and 1 resection) were examined. Treated areas showed more haemorrhage and possibly greater necrosis than untreated areas. Seven patients required opiate analgesia post procedure for 12 hours and 3 patients required extended hospital stay for pain control. Other side effects included macroscopic haematuria (2), massive abdominal wall bruising (1), biliary leak (1), jaundice (1).

**CONCLUSIONS** This study indicates that ILH is a feasible option for the treatment of hepatic malignacy. Cell necrosis and decreased tumour mass can be achieved despite the significant procedure related morbidity and therapy can be easily monitored by ultrasound. The value of ILH as an adjuvant to other therapeutic options in the management of patients with hepatic malignancy is worthy of study in a randomised clinical trial setting.

FP094 CRANIAL TRANSVERSE HEPATECTOMY

M Steves and P Sugarbaker

Washington Hospital Center
Washington, D.C. USA

With recent advances in liver surgery, liver resections with curative intent are being performed at a growing rate. However, there is a group of patients with lesions at the confluence of the hepatic veins who pose a problem for the hepatic surgeon. Extended resections for these lesions have a higher morbidity and mortality than lesser procedures (segmental resection). The use of the transverse hepatic plane to resect S5 and S6 has been previously reported. This surgically created plane can also be used to resect those lesions at the hepatic vein confluence. We report two cases of right and middle hepatic vein sacrifice with segmental liver resection of S7, S8, and S4A (cranial transverse hepatectomy).

In the last three years and seventy liver resections, our institution has seen several challenging lesions at the hepatic vein confluence. Two patients underwent cranial transverse hepatectomy for metastatic colon cancer. Each patient had a solitary lesion located at the junction of the IVC, RHV, and MHV. There were no intraoperative or postoperative complications. One patient received no blood transfusion and the other 6 units. Discharge was at 14 and 12 days postoperatively. Peak bilirubin was 3.7 and 3.3. Bilirubin at POD 10 was 2.9 and 1.6. Both patients are alive at 10 and 22 months.

Cranial transverse hepatectomy is a safe and useful segmental resection for lesions located at the hepatic vein confluence. The ability of the liver to develop collateral venous drainage to the lower segments (S5, S6, and S4B) is remarkable.

FP095

### PROBLEMS IN DIFFERENTIAL DIAGNOSIS OF CYSTIC TUMOURS OF THE PANCREAS

C Sperti, C Pasquali, F Capellazzo, *R Polverosi, S. Pedrazzoli

Semeiotica Chirurgica - University of Padua, *Radiology - Montebelluna, Italy

From 1970 to 1991 we observed 156 patients with suspected pancreatic cystic lesions: pre-operative diagnosis suggested a cystic neoplasm in 16 cases. Histologically, a pancreatic cystic tumour was found in 24/156 patients (15%), including 3 microcystic adenomas, 9 cystadenomas, 10 cystadenocarcinomas, 1 solid and papillary epithelial neoplasm, and 1 neuro-endocrine cystic tumour. A correct pre-operative diagnosis was made in 14 patients. US and CT showed a multilocular cyst in only 3/14 cases, while angiography showed a hypovascular lesion. Serum amylase levels were high in 3 patients (2 cystadenocarcinomas and 1 papillary-epithelial). Amylase in cystic fluid performed in 6 cases, was low in 4 (2 benign and 2 malignant) and high in 2 cases. CEA and CA 19-9 in aspirated cystic fluid were within normal range in 3 cystadenomas and high in 1 cystadenocarcinoma.

CT guided fine-needle cytology revealed epithelial lining in 8 mucinous tumours (1 benign and 7 malignant). Excluding 2 occasional surgical findings the other 7 patients had a pre-operative diagnosis of pseudocyst (4), carcinoma (2), biliary neoplasm (1). In 1 patient intra-operative frozen section of cyst wall was consistent with pancreatic pseudocyst and internal drainage was performed.

In our series a single technique or investigation was not able to give us a correct diagnosis, however fine-needle cytology and fluid determination of tumoural markers may help to reduce misdiagnosis of pancreatic cystic tumours. Caution is needed in evaluating cystic amylase content for differential diagnosis with pseudocysts.

FP096 EXPRESSION OF PANCREATIC MARKERS AND SURVIVAL IN RESECTED PANCREATIC CANCER

L.Colombo, G.Carcano, L.Dominioni, A.Benevento, V.Di Carlo*, R.Dionigi.

Department of Surgery, University of Pavia-Varese, Ospedale Multizonale di Varese,Italy
*Clin. Chir., Univ. Milan, H.S.Raffaele, Italy

Recent immunohistochemical and ultrastructural studies (1) have demonstrated that exocrine pancreatic tumors represent a heterogeneous cytological population with different immunophenotypic expressions.
Histologic heterogeneity might be responsible for their different biological behaviour; that could justify the local recurrent disease in cases treated by radical exeresis and expected to have a better prognosis according to TNM staging.
PATIENTS AND METHODS. The expression of gastrointestinal antigens DU-PAN-2, M1 glycoproteic antigen, cathepsin E, pepsinogen II (PG II) and CAR-5 antigen, has been retrospectively assayed by immunohistochemical methods in 70 cases of pancreatic cancer (66 ductal carcinoma and 4 cystoadenocarcinoma) radically operated. The antigenic expression has been correlated with tumour grading and survival.
RESULTS. Statistical evaluation was possible only for CAR-5 and PG II antigens. CAR-5 antigen was never found in cystoadenocarcinoma, and was expressed more frequently in G 3 (63%) than in G 1 and G 2 carcinoma (40%). PG II was expressed in all the cases of cystoadenocarcinoma, and was present in 43% of ductal carcinoma. Mean survival correlated to antigen expression was: 17.7 months in ductal carcinoma PG II POS and 10.3 months in ductal carcinoma PG II NEG ($p < 0.01$).
CONCLUSIONS.The data show that patients undergoing surgery for resection of ductal pancreatic carcinoma expressing PG II antigen have a more favourable prognosis. This information could be of value in planning treatment of pancreatic cancer.

(1) Sessa F. et al.: Ductal cancers of the pancreas frequently express markers of gastrointestinal epithelial cells. Gastroenterology (1990), 98: 1655-1665.

FP097 MORBIDITY AND MORTALITY OF TRADITIONAL AND EXTENDED LYMPHO-ADENECTOMY IN RESECTION OF PANCREATIC HEAD CANCER (PK)

S Pedrazzoli*, V Di Carlo§, R Dionigi#, F Michelassi@

F Mosca", P Pederzoli^

Departments of Surgery, University of Padova*,
Milano§, Varese#, Pisa", Verona^, Italy. Chicago@, USA

Extended lymphadenectomy (L) is reported to improve local recurrence rate and 5-year survival after curative resection of PK in two retrospective studies (1,2). In March 1991 we started a prospective, randomized, multi-centric study aimed at comparing the short and long term results of a pancreatoduodenectomy (PD) with conventional (R1) L or extended (R2) L in the treatment of PK. We now report on the short term results obtained in our first 48 pts (33 m, 15f, mean age 59.9 ± 10.6 years) operated on between March 1 1991 and October 31 1992.

| Extent of lymphadenectomy | R1 (n=24) mean ± SD | R2 (n=24) mean ± SD |
|---|---|---|
| Age (years) | 60.2 ± 12.4 | 59.7 ± 8.6 |
| Intra-op blood transf. (U) | 2.3 ± 1.5 | 2.0 ± 1.0 |
| Post-op abd drainage (ml) | 2196 ± 1520 | 2636 ± 4361 |
| Post-op day drains removed | 11.3 ± 4.7 | 10.1 ± 4.3 |
| Post-op day diet started | 10.4 ± 4.4 | 8.9 ± 2.8 |
| Length p o hospital stay | 22.9 ± 9.8 | 18.6 ± 6.3 |
| Post-op mortal./major morbid | 2 / 11 | 1 / 6 ** |
| Pancreatic fistula | 4 | - |
| Haemorrhage | 2 | 2 (1) |
| Cardiogenic shock | 1 (1) | - |
| Subphrenic abscess, pulm. embol. | 1 | 2 |
| Acute pancreatitis, hemobilia | 1 (1) | - |
| Renal insufficiency, pneumonia | 2 | 1 |
| Peptic ulcer | - | 1 |

( ) Patients who died; ** Chi square = 2.28 0.3 > P < 0.1. Only post-op major morbidity was statistically slightly better after R2 L; the amount of post op abdominal drainage may be greater after the extended L; no other difference was statistically significant. Operative time was about 30' - 60' longer in R2 L, although local pancreatic conditions interfered greatly with it. We conclude that extended L can be performed at least as safely as a conventional L in association with a PD for hopefully curative resection of PK. The reported effect (1, 2) on long term survival will be assessable in the next years.

1) Ishikawa Ann Surg 208,1988 2) Manabe Cancer 64, 1989.

Supported by CNR, contract n. 92.02236.PF39

FP098

### CANCER OF THE EXOCRINE PANCREAS. RESULTS OF 787 RESECTIONS.

H Baumel, M Huguier, JC Manderscheid

Departments of Surgery and Biostatistics, Montpellier, Paris.

This retrospective multicentric study was carried out in patients operated on from 1982 to 1989.
Overall postoperative mortality was 10%. A multivariate analysis showed that age > 70 years and visceral failure influenced postoperative mortality (P<0.05). In patients surviving more than 30 days, the median survival time was 12.3 months, and the 5-year actuarial survival rate was 12%. This rate was lower (P=0.001) in patients with lymph node involvement (4%) than without (20%). According to the type of procedure, operative mortality was higher (P=0.01) after total pancreatectomy (n=112) than after Whipple procedure (n=555), 17% and 8%, respectively. Median survival times and 5-year actuarial survival rate after total pancreatectomy and Whipple procedure were 11 months and 14 months, and 3% versus 15%, respectively. The site of the tumour on the left pancreas, and left pancreatectomies (n=120) were related to a poor prognosis without any 4-year survivors. After Whipple procedure, a pancreatic fitulae occured in 9% of cases with pancreaticojejunal anastomosis (n=366), in none after pancreaticogastrostomy (n=16), and in 21% of cases after ductal obturation (n=24).
These results suggest that resection must be avoided in patients over 70 years old with visceral failure. The Whipple resection remains the gold standard type of resection. Total pancreatectomy is only indicated in multicentric tumours, and if a pancreatic anastomosis cannot be safely constructed. Left pancreatectomy for cancer of the corpus or tail of the pancreas may be considered as a palliative procedure. After Whipple procedure, pancreaticogastrostomy seems to be a safer procedure than pancreaticojejunostomy.

FP099

### CANCER OF THE EXOCRINE PANCREAS. RESULTS OF PALLIATIVE PROCEDURE IN 2493 PATIENTS

H Baumel, M Huguier, JC Manderscheid

Departments of Surgery and Biostatistics, Montpellier, Paris.

Opinions are still divided regarding the optimal palliative procedures in patients with cancer of the pancreas. This retrospective, multicentric study, was carreid out in patients operated on from January 1982 to December 1988 to compare the results of various procedures aimed at palliation for pancreatic cancer.

Cholecystoenteric bypasses (n=237) in comparison to choledochoenteric bypasses (n=1770) were associated with a higher postoperative mortality (20% vs 14%), a lower long-term morbidity (26% vs 35%), and a lower survival rate (means: 13.2 versus 5.2 months). Choledochoduodenostomy (n=1159) and choledochojejunostomy (n=611) had similar rates of postoperative mortality (14% vs 13%), morbidity (26% vs 27%), incidence of recurrent jaundice (8% vs 7%), and median survival (5.4 vs 5.0 months). Surgically placed biliary stents (n=114) were followed by the highest postoperative mortality (27%), morbidity (46%), rate of recurrent jaundice (14%), and the shortest median survival (2.6 months). Postoperative mortality in patients undergoing a choledochoenteric bypass and a gastrojejunostomy (n=1134) was similar to that observed in patients who had only a biliary bypass (n=636) (16% and 12%), but among the patients who had a biliary bypass alone, 16% developed a gastric obstruction. For the relief of pancreatic pain, radiotherapy was more effective than other symptomatic treatments (p=0.02).

These results suggest the need 1) in patients with obstructive jaundice to perform a choledochoduodenostomy rather than other biliary bypasses, 2) a routine prophylactic gastrojejunostomy to prevent gastric outlet obstruction, 3) and for the relief of pancreatic pain to perform radiotherapy or splanchnicectomy.

FP100

### ROLE OF OCTREOTIDE COMBINED WITH TPN AND ANTIBIOTICS IN THE TREATMENT OF PANCREATIC DISEASES AND PREVENTION OF POSTOPERATIVE COMPLICATIONS FOLLOWING PANCREATIC RESECTION

BM Wang, Y Tang, XG Hu, R Liu, QF Liu
Department of Surgery, Chang Hai Hospital
Shanghai 200433, PR of China

Pancreatic exocrine secretion decreased significantly from the stent drainage tube inserted into the pancreatic duct stump during a Whipple procedure for patients with periampullary cancer (n=10)after receiving a 7—day treatment of 100μg octreotide (Sandostatin) subcutaneously Q8h, as compared with the control group (n=8) ($P<0.01$).
Octreotide was given I. V. in saline with a dosage of 5μg/Kg/h for 3 hours in rats with acute hemorrhagic pancreatitis established by the injection of 5% sodium taurocholate into the pancreatic duct. The results showed that octreotide could decrease the serum levels of amylase and lipase effectively, reduce lung index and extravascular lung water significantly, as well as ameliorate the pathologic changes in lung and pancreas of rats, as compared with the control group ($p<0.01$).
During a period of 1 year (Dec. 1991—Nov. 1992), 3 cases of pancreatic fistula and 5 cases of acute hemorrhagic pancreatitis were treated non—operatively with octreotide, TPN plus antibiotics, and recovered uneventfully. During the same period, no pancreatic fistula was observed in any of the 21 consecutive patients folllowing a difficult Whipple procedure after receiving octreotide in conjunction with TPN and antibiotics perioperatively, as compared with the historical control. It is concluded that octreotide is an invaluable drug in conjunction with TPN and antibiotics for the treatment of certain pancreatic diseases and prevention of postoperative complications following pancreatic resection.

FP101

### PERCUTANEOUS US-GUIDED FINE NEEDLE ASPIRATION CYTOLOGY IN THE DIFFERENTIAL DIAGNOSIS OF PANCREATIC AND PERIPANCREATIC MASSES

A Campatelli, F Farina, G Sartoni, P C Giulianotti,
G Di Candio, U Boggi, F Mosca

Istituto di Chirurgia Generale e Sperimentale
Pisa University Medical School, Italy

Although recent improvements in imaging techniques (US, CT and ERCP), the differential diagnosis of a pancreatic mass still remains a challenging problem. In some cases fine needle aspiration cytology (FNAC) has been proposed as a helpful diagnostic tool.
Out of 186 patients admitted to PISA University Hospital with a diagnosis of pancreatic or peripancreatic mass, in the period between November 1987 and July 1992, forty-one (22%) (22 males; 19 females; mean age 59 ± 9.3), who had already had a routine diagnostic work-up (i.e. US, CT and ERCP), underwent US-guided FNAC to define diagnosis and treatment.
Patients were retrospectively divided into 2 groups on the basis of whether or not the clinical judgement suggested a surgical approach regardless of FNAC results: Group A (surgery group) (n=36) and Group B (no surgery group) (n=5).
Twenty-three lesions were located in the head of the gland, 9 in the body and 9 in the tail; thirty-five were solid and six cystic masses. Mean US diameter was 37mm (range 10 - 80mm). A 22-gauge spinal needle was inserted under US guide. Sufficient material for cytology was available after the first sampling in thirty-eight patients (92.68%) and in three a second or third attempt was required.

GROUP A (n=36)

| FNAC diagnosis | Operative Specimen |
|---|---|
| 18 AdenoCa | 18 AdenoCa |
| 4 possible Ca | 4 AdenoCa |
| 1 Carcinoid tumour | 1 Carcinoid tumour |
| 2 Cystoadenoma | 2 Cystoadenoma |
| 9 Pancreatitis | 3 Pancreatitis |
| | 6 AdenoCa |
| 1 Adenoma | 1 AdenoCa |
| 1 Lymphoadenitis | 1 Lymphoadenitis |

7 False negative: Sensitivity 80%

GROUP B (n=5)

| FNAC diagnosis | Notes |
|---|---|
| 1 Hodgkin lymphoma | Previous positive lymph node biopsy |
| 1 AdenoCa | Recurrence after PPPD* |
| 1 AdenoCa | Dead from carcinosis |
| 1 Metastasis of HHC | Dead from HHC |
| 1 Chronic pancreatitis | Alive |

*PPPD: pylorus preserving pancreatoduodenectomy

No procedure (FNAC) related complication was recorded. Neither local nor diffuse peritoneal metastatic spread was observed (samples of peritoneal lavage cytology were routinely examined after surgery).
In conclusion in Group A, patients in whom surgery is indicated after a standard diagnostic work-up, FNAC does not add any further element in treatment decision making. Findings suggestive for chronic pancreatitis do not actually exclude the presence of an underlying cancer and resection should be performed whenever the clinical judgement suggests to do so. On the other hand a positive cytology (specificity 100%) may be of help in planning therapy (i.e. combined antibalstic pre-operative neo-adjuvant treatment).

In Group B FNAC may be useful in clarifying diagnosis (specificity 100%), guiding follow-up therapy.

FP102

### 153 PYLORUS PRESERVING PANCREATODUODENECTOMIES: A CLINICAL AND PHYSIOLOGICAL SURVEY.

F Mosca, P C Giulianotti, U Boggi, G Sartoni, F D'Elia, G Di Candio, T Balestracci, M Oleggini

Istituto di Chirurgia Generale e Sperimentale
Pisa University Medical School, Italy

From January 1982 to November 1992 153 pylorus preserving pancreatoduodenectomies (PPPD) (94 m, 59 f),including 10 total pancreatectomies, were performed at the Department of General and Experimental Surgery of Pisa University. 20 patients had chronic pancreatitis, 73 ductal adenocarcinoma (including 3 patients who had a synchronous neoplasm: two of the papilla of Vater and one of the kidney), 28 papillary cancer, 13 common bile duct cancer, 4 duodenal cancer, 4 cystadenocarcinoma, 2 cystadenoma, 2 non-functioning endocrine tumour, 1 carcinoid tumour, 1 squamous carcinoma, 1 inslet cell carcinoma, 1 duodenal leiomyosarcoma, 1 lymphoma, 1 papillary-cystic neoplasm and one benign tumour of papilla of Vater.

Mean age was 63.41 $^{+}/_{-}$ 12.9 yrs. and 48.85 $^{+}/_{-}$ 12.03 yrs. in the neoplastic and in the chronic pancreatitis group respectively. Operative mortality (overall rate 7.18% [11/153]) has shown a significant decrease while gaining further surgical experience and is now 2.6% (2 deaths out of 75 operations in the last 4 yrs.). Half of the deaths occurred from cardiovascular diseases. Morbidity rate was 41.8% [64/153] and 6 patients required re-operation. Postoperative naso-gastric suction maintained for an average of 12.3 days (range 5-27) and for more than two weeks ( delayed gastric emptying) in the 26% of cases (36/146). Food re-introduction was satisfactorily accomplished in the majority of the patients. The weight gain average, six months after surgery (excluding those who had an early neoplastic recurrence) was 3.8 kg (some patients gained as much as 7-10 kg). Specific late complications were observed in 5 patients: 2 perforated gastric ulcers requiring emergency surgery (one due to chemotherapy for lymphoma and the other to aspirin abuse 5 months following surgery), 1 duodenal bleeding (42 months after surgery) and 2 antral gastritis with mild bleeding. The latter three were successfully treated with medical therapy.

All the patients operated on for chronic pancreatitis but one, who died of laryngeal cancer, are still alive. Late complications in this group were: 1 stricture of hepaticojejunostomy (successfully treated by percutaneous balloon dilation) and one small intestinal obstruction due to adhesions, which was relieved by medical therapy. A satisfactory pain control was obtained in every case.

Out of 73 patients operated on for pancreatic cancer 14 are still alive (with three surviving longer than three yrs.). The overall three yrs. survival rate is 25.64% (10/39). 47 patients died of cancer recurrence: the mean survival time in this group was 14.19$^{+}/_{-}$ 12.9 months. A retrospective analysis comparing two homogeneous groups of patients who underwent PPPD (n=41) or Whipple procedure (n=16) did not show significant differences in the survival time (median survival time: PPPD 14$^{+}/_{-}$ 1.9 months; Whipple 13$^{+}/_{-}$ 3 months) (Life table and survival function [Breslow and Mantel-Cox test] and Cox proportional regress hazard model).

FP103

### LIVER TRANSPLANTATION FOR VIRAL HEPATITIS A REVIEW OF 31 CASES

S Bhattacharya, G Dusheiko, A Burroughs
B Davidson, K Rolles

Royal Free Hospital and School of Medicine
London NW3 2QG, U.K.

From October 1988, 32 liver transplantations were performed on 31 adults with acute or chronic viral hepatitis.

Two were transplanted for fulminant viral hepatitis B. Both are alive 8 to 12 months later, with no evidence of reinfection.

Of 11 patients with chronic hepatitis B, 2 died of early post-operative complications. Three have had their HBsAg eradicated, and remain well at 10, 26 and 43 months. Six suffered HBV reinfection of the graft. Return of HBsAg and HBV DNA in serum was followed by clinically apparent graft dysfunction. All six have died of liver failure, with a mean post-operative survival of 9 months.

Two patients had chronic hepatitis B and D. Both are now HBsAg negative, though one suffered an episode of acute HBV graft infection. Two patients had chronic hepatitis B and C, and both succumbed to HBV graft reinfection at 3 and 7 months respectively.

All but one patient with HBV infection received prophylactic hepatitis B immune globulin (HBIG). Of the 8 patients who died of HBV reinfection, 4 were negative for HBV DNA and HBeAg when transplanted.

Of 14 patients transplanted for chronic hepatitis C, there was one early mortality. Two were cleared of HCV RNA, though one died later from unrelated causes. The remaining 11 all developed viral reinfection (positive HCV RNA). One died of liver failure at 23 months, and 3 have liver dysfunction attributed to HCV reinfection of the graft. The mean follow-up of the 10 HCV RNA positive survivors is 9 months.

Excluding early mortalities, 9 out of 10 deaths were due to viral reinfection of the graft. In chronic hepatitis B, despite careful patient selection and attempted prophylaxis with HBIG, graft failure from HBV reinfection remains the major cause for late morbidity and mortality. In patients with chronic hepatitis C, reinfection with HCV is common, but it does not have as damaging an effect on graft function.

FP104

### A PROSPECTIVE SERIES OF LIVER TRANSPLANTATION (OLT) FOR SMALL HEPATOCELLULAR CARCINOMA(HCC) IN CIRRHOSIS

V.Mazzaferro,E.Regalia,G.Colella,
R.Doci,F.Bozzetti and L.Gennari

Istituto Nazionale Tumori Milan-Italy

From January 1991 to December 1992 24 OLT were performed in 22 patients affected by HCC in cirrhosis. The acceptance criteria chosen for the prospective accrual of such patients were:1) non resectable single nodule < 5 cm or multifocal HCC (< 3 nodules, < 3 cm ) 2) pre-operative T1-2,No,Mo 3) histologically proven cirrhosis. 12 cirrhosis were HCVAb+, 7 had a HBsAg+/HBV DNA- status and in 3 cases B and C viruses were associated. Pre-operative Child-Pugh stage was A=5 pts, B=7 pts, C=10 pts.

In 15 pts (68%) pre-transplant chemoembolization (CE) with Lipiodol, Gelfoam and Doxorubicin, Mitoxantrone or Mitomycin C was feasible. Although a necrosis of > 50% was observed in 41% of the HCC-nodules,the exact role of CE on pts-survival is not clear since no tumor recurrence was detected neither in the CE-pts nor in untreated OLT.

Two pts (9%) underwent re-OLT (primary non-function and fulminant HBV reinfection) while complex arterial reconstruction (iliac graft) occurred in 41% of cases. Perioperative mortality (1st month) was 18%, median stay in ICU was 10 days (4-62). Post-transplant HCC stage was pTx=2, pT1-T2=13, pT3=6, pT4=1. Lymphnodes were free of tumor in all cases. Six out of 15 pts (40%) had a mild form of HCV recurrence and 2 out of 10 pts (20%) had a severe HBV recurrence.

One-year survival of the present series is 78% and up to now all deaths are due either to post-OLT complications (ARDS, GvHD, multiple organ failure and sepsis) or to HBV recurrence. No tumor recurrence has been observed after a median follow-up of 12 months. OLT for HCC in cirrhosis seems to be justified in early tumor stages.

FP105

### ARTERIAL CHEMOEMBOLIZATION (CE) BEFORE LIVER TRANSPLANTATION FOR HEPATOCELLULAR CARCINOMA IN CIRRHOSIS

E.Regalia, G.Colella, C.Spreafico, A.Marchiano', S.Andreola,F.Montalto, R.Doci, F.Bozzetti, L.Gennari and V.Mazzaferro.
Istituto Nazionale Tumori Milan-Italy

From November 1990 to December 1992, 24 orthotopic liver transplants (OLT) were performed on 22 patients with small unresectable hepatocellular carcinoma (HCC) in cirrhosis. 15 out of 22 pts (68%) were treated pre-operatively with CE with Lipiodol, Gelfoam and different drugs schedule: Doxorubicin(30 mg/m2) =9pts; Mitoxantrone(14 mg/m2) =4pts and Mitomycin-C(20 mg/m2) =2pts. A total number of 33 cycles were given with a mean of 2.2 for patient (range 1-4). CE was not performed in 7 pts either for advanced Child stage or for technical problems.
Complication rate was 40% ( medullary aplasia:2 pts; fulminant liver failure requiring emergency OLT:3 pts; hepatic artery intimal dissection:4 pts ) but no pts died from the procedure.
Hystological response in terms of % of tumor necrosis after total hepatectomy was evaluated on 22 nodules of HCC (mean diameter 1.9 cm - range 0.8-5.1).
Necrosis >50% was observed in 9 nodules (41%), partial response (<50% necrosis) in 7(32%) and 5 nodules (23%) were unaffected by CE. Number and diameter of nodules, presence of capsule, type of drug and number of cycles were not significantly associated with response to CE while the hypervascolar aspect on the angiogram was significantly associated with necrosis > 50% (p=0.004). The pT stage of the tumor was correlated with response to CE ( necrosis >50% in 57% of T1-T2 nodules vs 12% of the T3 p=0.05).
Longer follow-up is needed to assess the influence of CE on patients-survival since no tumor recurrence was detected after OLT at a median follow-up of 12 months.

FP106

### LIVER TRANSPLANTATION IN CHILDREN WITH CIRRHOSIS AND SEVERE HYPOXEMIA

J HOBEIKA*,D HOUSSIN*,O BERNARD°,D DEVICTOR°, G GRIMON°, Y CHAPUIS*

Clinique chirurgicale,Hôpital Cochin,PARIS FRANCE

Département d'hépatologie pédiatrique Hôpital Kremlin-bicêtre,le Kremlin-Bicêtre.FRANCE

Liver transplantation has been considered until recently as an absolute contraindication in hypoxemic patients with intrapulmonary shunting (IPS)because of a high mortality. We report our experience of orthotopic liver transplantation (OLT) in nine patients with cirrhosis-related hypoxemia.

Patients and methods: Nine patients with a median age of 9 years (28 months-17 years) having cirrhosis-related hypoxemia had orthotopic liver transplantation (OLT) between June 86 and June 92. The arterial blood oxygen pressure (PaO2) at room air ranged from 47 to 78 mm Hg. Hypoxemia was related to intrapulmonary shunting(IPS) in all cases as documented by technetium 99-m macroaggregated albumin scintigraphy (MAA* scintigraphy) and pulmonary angiogram. OLT was performed 9 years (2-15 years) after the first symptom of liver disease and 36 months (4-108 months ) after the first respiratory symptom. As a mean,operation time and cold ischemia time were 11 hours (6-19 hours) and 10 hours (4.5-22 hours),respectively. Two patients had veno-venous by-pass during OLT.

Results:Four patients who had preoperative PaO2 at air level lower than 60 mmHg died between the second and the 37th post-operative day ,three from worsening hypoxemia requiring 100% oxygen ventilation and leading to cardiopulmonary failure and one from primary graft non-function. Five patients survived.Two of them had preoperative PaO2 level lower than 60 mmHg. Two patients had acute graft rejection successfully treated by corticosteroids on the 12th and the 21th post-operative day. One patient required reoperation for biliary anastomotic stricture. Two patients had symptomatic cytomegalovirus infection without pulmonary infection successfully treated by DHPG. The median hospitalization time in intensive care unit was 9 days (4-22 days). Median endotracheal tubing time was 6.8 days(2-19 days) All patients demonstrated normal blood gas analysis 3 months after OLT with closure of IPS, as documented by MAA*scintigraphy.

Conclusion: Severe hypoxemia is no longer a contraindication to liver transplantation .Patients having PaO2 levels higher than 60 mm Hg should have OLT as soon as possible before reaching lower levels of PaO2. Because of the high operative risk of liver transplantation, combined lung-liver transplantation should be considered in patients with more severe hypoxemia(PaO2<60 mm Hg).

FP107 VALUE OF DONOR MEGX LEVELS AND HISTOLOGY IN PREDICTING GRAFT OUTCOME

K Karayalcin, D Mirza, R Harrison,
R DaSilva, S Hubscher, AD Mayer,
JAC Buckels, P McMaster.

The Liver and Hepatobiliary Unit, Queen Elizabeth Hospital, Birmingham, U.K.

Despite their limitations, conventional tests of liver function and macroscopic appearance of the donor liver have been the mainstay in selection of donor livers for transplantation. The use of donor liver biopsies and the cytochrome P-450 mediated formation of lignocaine metabolite mono ethyl glycine xylidide (MEGX) has been reported to be useful in predicting early graft function.

This prospective study included 63 consecutive livers harvested and transplanted over a six month period. Serum MEGX levels were assessed 15 minutes after an intravenous bolus of 1 mg/kg lignocaine. Preperfusion liver biopsies were obtained at harvesting prior to any dissection and postperfusion liver biopsies after completion of vascular and biliary anastomoses, and these were examined for features of pre-existing disease and effects of preservation injury. No grafts were discarded on the basis of MEGX levels or histology, and all harvested livers were transplanted.

The median MEGX level was 89 μg/L (16-250); 15 had MEGX levels < 50 μg/L, 17 between 50 and 90 μg/L, and 31 > 90 μg/L. There ware no significant differences in early graft function and outcome in the three groups. Preperfusion biopsies were reported as normal (n=33), mild steatosis (n=17), moderate/ severe steatosis (n=11). All grafts showed good function, including those with moderate/ severe steatosis. There was no case of primary nonfunction. MEGX levels failed to correlate with donor histology. Examination of postperfusion biopsies for effects of preservation injury (minimal, mild, moderate) revealed no difference in graft outcome, although the livers with moderate/ severe steatosis showed a significantly greater degree of preservation injury ($p<0.01$).

FP108 LIVER TRANSPLANTATION IN PATIENTS WITH POSITIVE T LYMPHOCYTOTOXIC CROSSMATCH

T. R. Kurzawinski, Z. Varghese, S. Hardy, J. Bed, G. Shirling,
A. Burroughs, K. Rolles.
Hepatobiliary & Liver Transplantation Unit, Tissue Typing
Laboratory, Royal Free Hospital, London, U.K.

The effect of a positive T lymphocyte crossmatch on the outcome of orthotopic liver transplantation (OLT) is conflicting.

The aim of this study is to evaluate the influence of a positive T cell crossmatch on the outcome of orthotopic liver transplants performed in the Royal Free Hospital between 1988 and 1992.

One hundred and forty-two patients undergoing OLT had a T cell crossmatch performed retrospectively by the cytotoxicity method. The results of the crossmatch did not influence recipient selection, which was based on medical indications for transplantation only.

Eight patients (6%) (four males, four females, median age 46 years, range 31-64) had circulating cytotoxic antibodies to donor T cells detected in serum taken hours before transplantation. Indications for transplantation in these patients were Hepatitis B (1), Hepatitis C (2), alcoholic cirrhosis (1), epithelioid haemangioendothelioma (1), primary biliary cirrhosis (1), chronic liver graft rejection (1) and cryptogenic cirrhosis (1). The past medical history of these patients included pregnancy (4/4) and blood transfusion (6/8) before transplantation. Five patients were treated with Cyclosporin A, Methylprednisolone and Azathioprine post-operatively and three patients with anti-thymocyte globulin, Cyclosporin A and Methylprednisolone.

Cytotoxic antibodies against donor T cells disappeared from the circulation within the first 48 hours after liver transplantation. All patients experienced one to four episodes of biopsy proven moderate to severe acute cellular rejection. Rejection episodes were treated with 1 gram of Methylprednisolone per day for three days. Additional OKT3 therapy was given to three patients following an unsatisfactory steroid response. No case of primary graft non-function or graft loss for any reason were seen. The earliest episode of acute cellular rejection was seen on the fifth post-operative day. A single patient developed chronic rejection of the liver graft at 12 months. Thrce of the eight patients died; one chronic rejection at 17 months, one tumour recurrence at 4 months, one infection at 5 months. Five out of eight patients are currently alive with normal liver function. No radiological or histological evidence of vanishing bile duct syndrome has been seen in any of the patients. Actuarial one year survival in this group is 69%.

We conclude that a positive T cell lymphocytotoxic crossmatch has not significantly effected the outcome of transplantation in our small series.

FP109

### ZOLLINGER ELLISON SYNDROME. SURGICAL TREATMENT FOR "CURE" OR LONG TERM PALLIATION?

C Pasquali, C Sperti, D Miotto*, A Alfano D'Andrea, C Filipponi, S Pedrazzoli

Semeiotica Chirurgica & Radiology*, University of Padua, Italy

Since 1970 we observed 51 patients with Zollinger-Ellison syndrome; 11 had a MEN type 1 with two or more endocrine glands involved. 24 patients had a Portal Venous Sampling (PVS) before surgery for tumour localization and evaluation of resectability. 28/50 patients underwent surgery; 11 had only a total gastrectomy because the tumour was not found. 17 patients underwent surgery with resective purpose; 13/17 had a PVS performed before surgery which in 8 cases showed only a single source of gastrin release, then hopefully "curable". All these 8 cases became normogastrinemic after operation (follow-up 5 to 16 years). Only 1/5 cases in which PVS was not localizing a single tumour was cured by surgery. Only 1/4 patients who underwent surgery without prior PVS was made normogastrinemic by resection of a large liver tumour involving the right lobe.Then in 10 out of 28 patients surgically treated,gastrin fell to normal,but in two cases a late recurrence of hypergastrinemia was found after 5 and 14 years.All the patients in which gastrin did not fall to normal after surgery were treated with antisecretory drugs (if not gastrectomized) as well as those who had no surgery at all.Out of 26 patients who had medical treatment 3 escaped and needed emergency surgery later (3 mo.-3 yrs.) while 9 required omeprazol for long term failure of H2 blockers (3-14 yrs.)

5 patients had liver metastases at the time of diagnosis; 2 died within 12 months. Two cases (1 total gastrectomy and 1 who had only medical treatment) developed liver metastases 21 and 15 years later respectively. PVS greatly enhanced the chance for tumour resection and gastrin normalization after surgery; late recurrence may occur even after 5 years due to slow growing tumour. So, despite gastrin falling to normal, tumour resection may represent a long term palliation; careful and lifelong follow-up is mandatory even in "cured" and total gastrectomized patients.It is strongly advisable not to leave patients in chronic medical treatment without periodical re-evaluation for possible resective surgery.

FP110

### PREOPERATIVE LOCALIZATION OF INSULINOMAS BY ENDOSCOPIC ULTRASONOGRAPHY.

J Pitre, O Soubrane, L Palazzo, Y Chapuis

Clinique Chirurgicale, Hôpital Cochin, Paris, France

Pre-operative radiological localization of insulinomas often fails because of the small size of these tumors. However, the tumor localization is of great interest before surgery in order to avoid any blind pancreatic resection. Thus, the aim of this study was to assess the value of endoscopic ultrasonography (US) in preoperative localization of insulinomas.

Between 1983 and 1992, 14 patients with hyperinsulinism were evaluated for tumor localization before surgical resection. Radiological assessment included: abdominal US (n=13), CT-scan (n=13), angiography (n=5), magnetic resonance imaging (n=5), portal venous sampling (n=1), and endoscopic US for the last 7 patients. All patients underwent laparotomy with intra-operative US.

During surgery, the association of palpation and US localized 12 solitary tumors and 2 multiple tumors (including a case of multiendocrinopathy type I), confirmed at histological examination. Four tumors were found malignant because of lymph node involvement and/or liver metastasis. The mean size of the tumors was 19±11 mm. The sensitivity of conventionnal preoperative methods of localization was 6/14 cases whereas the sensitivity of endoscopic US was 6/7 cases with no false positive case. No blind pancreatic resection was done. The surgical procedures were: 5 enucleations, 8 distal pancreatectomies (4 without splenectomy) and one total pancreatectomy. No postoperative death occurred.

We conclude that because of its high sensitivity and safety, endoscopic US is the best method for preoperative localization of insulinomas whereas the conventional methods are of limited interest and should be abandoned.

FP111 LOCALIZATION AND SURGICAL MANAGEMENT OF INSULINOMAS

C Pasquali, C Sperti, G Liessi §, A Alfano D'Andrea, C Filipponi, M Guido*, S Pedrazzoli

Semeiotica Chirurgica - University of Padua, § Radiology - Castelfranco * Pathology - Cittadella, Italy.

From 1966 to 1992, in our Department 65 cases with organic hyperinsulinism were observed. A single case had a MEN type 1. 58 patients underwent surgery; 5 of them had a second operation because the first failed to find the tumour. Pre-operative investigations included: arteriography in 56 cases, a CT scan in 32, US scan in 21, portal venous sampling in 28, NMR in 12. 23 patients had also a pre-operative US scan. Arteriography was positive in 20/60 performed, (33 %), with 10 false positive (10 %). Only 12/33 CT (36 %) and 9/21 (43 %) US scan were positive; false positives were 9 % and 5 % respectively. NMR was positive in 7/12 cases. Portal venous sampling (PVS) was positive in 23/28 cases (82 %). Operative US localized the tumour in 17/23 (74 %) cases, including 4 cases in which pre-operative US was negative and 2 cases of "occult" insulinoma. In the last 9 year period (22 patients) the positivity rate for imaging techniques rose to 50 % for angiography, 56% for CT scan and 64% for US in patients with adenoma, but three patients underwent surgery having only a positive PVS.

In 63 operations performed, 18 cases (29%) had a negative surgical exploration and at least 1 adenoma was missed by palpation and occasionally found in three more cases. Multiple tumours were found in 5 while in 5 were found a nesidioblastosis or Beta cell hyperplasia. Only 2 cases had liver metastases at surgery. An explorative laparotomy was performed in 4 cases, a pancreatico-duodenectomy in 2, a left pancreatectomy in 34 (in 15 the spleen was preserved), a tumour excision in 18, and an atypical pancreatic resection in 3. 53/58 patients (91%) became euglycaemic after surgery, 2/58 died from tumour progression and in 2 cases the tumour was not found (3.4%). Morbidity was 23% and mortality was 6%, however only one death, unrelated to surgery occurred in the last 45 cases.

FP112 NON-FUNCTIONING ISLET CELL TUMOURS OF THE PANCREAS.

R.C.N. Williamson, S. Cheslyn-Curtis

Department of Surgery, Royal Postgraduate Medical School and Hammersmith Hospital, London

Pancreatic neuroendocrine tumours are rare and most produce a clinical syndrome due to the excess production of a single hormone. Between 15-41% appear to be non-functioning producing neither a clinical syndrome nor an excess of any active peptide. We have reviewed the natural history and management of non-functioning tumours diagnosed between 1982 and 1991. There were 20 patients of median age 44 yr (22-75 yr). Presenting features were obstructive jaundice (7), abdominal pain (7), weight loss (6), abdominal mass (6), and severe haemorrhage (4). Gut hormone profiles were normal except in one patient with a raised pancreatic polypeptide level. Contrast-enhanced computed tomography localised the tumour in 17 patients and visceral angiography in 14 of 15 patients; all but three tumours were highly vascular. Ten patients underwent curative resection and the remainder were managed palliatively by resection, bypass procedures or biopsy alone. There were two postoperative deaths and 7 early complications. Seven of the remaining 18 patients have died of their disease at a median 16 months (4-30 months) following presentation. The 11 survivors, 8 of whom had "curative" resections have been followed for a median 42 months (7-72 months). Ten patients are asymptomatic although only 5 are free of disease. These tumours are seldom curable by radical surgery but patients may remain free of symptoms for many years.

FP113 The use of an isotope labeled somatostatin analog in the visualization of pancreatic tumors enlarges the indication for operative therapy.

C.H.J. van Eijck, S.W.J. Lamberts, E.P. Krenning, J. Jeekel.
Ac. Hosp. Dijkzigt,
Dr. Molewaterplein 40, 3015 GD Rotterdam, The Netherlands.

Since palliative surgery in patients with islet cell tumors is not only of value to relief clinical symptoms but also to decrease tumor burden, which facilitates the effect of causal medical treatment, preoperative differentiation between pancreatic adenocarcinomas and "non-functioning" islet cell tumors is essential.
We recently developed a new technique for visualization of "non-functioning" islet cell tumors preoperatively. We tested this new technique in 25 patients with islet cell neoplasms after the intravenous administration of the isotope labeled somatostatin analog $^{111}$In-Octreotide.
The primary tumors, as well as previously often unrecognized distant metastases were visualized in 20 of these 25 patients (80%) with islet cell tumors.
All 6 "non-functioning" islet cell tumors could be visualized. In contrast in 20 patients with primary pancreatic adenocarcinomas the tumor could not be seen.
This new technique of in vivo localisation of somatostatin receptor positive tumors may select more candidates preoperatively for palliative surgery, especially patients with "non-functioning" islet cell tumors which by other means cloudn't be recognized and the detection of somatostatin receptors on these tumors in vivo predicted a good suppressive effect on hormonal hypersecretion by these tumors and may help to predict and monitor the growth inhibitoiry effect of octreotide.

FP114

### PANCREATIC MICROCYSTIC ADENOMAS (GLYCOGEN-RICH CYSTADENOMAS). A CLINICOPATHOLOGIC STUDY OF 20 CASES.

D. BRASSIER - L. De CALAN
Association de Recherche en Chirurgie
Association Universitaire de Recherche en Chirurgie
Hopital R. Ballanger - Service de Chirurgie Viscérale
93600 AULNAY SOUS BOIS

Pancreatic microcystic adenoma is a rare cystic neoplasm which is benign. This retrospective multicentric study (from january 1978 to december 1987) reports the clinicopathologic features and results of treatment of 20 patients with microcystic adenoma of the pancreas.

There were 4 men and 16 women. The median age was 62 years (31-82). The tumour was found incidentally in 10 patients (3 times during an operation for other disease, 7 times during abdominal investigation for other disease); 10 patients had a palpable abdominal mass with or without abdominal pain. One patient has two tumours, 19 patients had a single tumour with a mean size of 7cm, which was located in the head (6 patients), the body (5 patients) or the tail of the pancreas (8 patients). Two patients had only a biopsy of their tumour, 18 patients underwent a complete excision of their tumour : 4 Whipple procedures, 12 distal pancreatectomies, 2 tumorectomies. There were two post-operative deaths (one patient operated on for a carcinoma of the colon, one patient operated on for a carcinoma of the rectum). One patient was lost to follow-up. The remaining 17 were alive and well, without evidence of recurrence, with a median follow-up of 16 months.

This results confirm that microcystic adenoma of the pancreas is benign and can be managed conservatively. When the tumour occurs in the body or tail of the pancras a distal resection can be carried out. On the other hand, when the tumour occurs in the head of the pancreas, surgical removal may not be necessary if the diagnosis can be made by biopsy with frozen section, especially in the elderly. If there is a gastrointestinal or biliary obstruction a bypass can be performed.

FP115 RISK FACTORS AND OUTCOME OF GUT PERFORATION AFTER LIVER TRANSPLANTATION FOR BILIARY ATRESIA.

O Soubrane, M El Meteini, D Houssin, O Bernard, Y Chapuis.
Clinique Chirurgicale, Hôpital Cochin, Paris
FRANCE

The incidence of gut perforation after liver transplantation (LT) for uncorrectable biliary atresia (BA) ranges from 5 to 30 %. Because it is a potentially lethal complication in immunosuppressed patients, the present study aimed at evaluating the risk factors and prognosis of such gut perforations.

From april 86 to february 92, 61 LT were performed in 51 children with uncorrectable BA. Gut perforation occurred in 20 % of the patients. Two groups of patients were therefore individualized: group A of 10 patients with 19 episodes of gut perforation arising 5 to 68 days after 14 LT and group B of 41 patients without gut perforation after 47 LT. These two groups were compared in order to identify risk factors and a stepwise regression analysis about 12 factors possibly associated with the occurrence of digestive perforations was carried out.

The recipients' age and body weight, the number of cholangitis episodes after Kasai operation, pre-transplant liver biological tests and the number of reduced-size grafts used were not different between the two groups. On the contrary, the amount of red blood cells transfused during the recipient hepatectomy and the duration of LT were significantly greater in group A. The incidence of severe fungal infections was 65 % in group A vs 2.5 % in group B ($p<0.001$). There was no difference between the two groups regarding early occurrence of acute rejection episodes and subsequent use of steroid pulses, as well as early CMV infections. The stepwise regression analysis identified 3 factors significantly and independently associated with the occurrence of gut perforation: duration of LT, post-transplant intraperitoneal bleeding requiring reoperation and post-transplant portal vein thrombosis. The survival rate was not different between the two groups: 70 vs 80 %, respectively after a median follow-up of 32 months.

In conclusion, the occurrence of gut perforations seemed especially related to the technical difficulty of the recipient's hepatectomy whereas the influence of steroids and CMV infections was not significant. This type of complication, formerly known to bear a poor prognosis in organ transplant recipients, led in this series to a survival rate as high as 70 % despite the immunosuppression and high incidence of fungal infections.

FP116

### CORRELATION BETWEEN INFLAMMATORY CYTOKINES, ENDOTOXIN, AND ISCHEMIA IN PATIENTS DURING AND AFTER ORTHOTOPIC LIVER TRANSPLANTATION

S Tange, Y Höfer, W Ertel, M Welte*, E Pratschke, K Jauch, E Faist, F-W Schildberg,

Department of Surgery, Klinikum Grosshadern, LMU Munich * Department of Anaesthesiology, Klinikum Grosshadern, LMU Munich, Germany

Inflammatory mediators released by residual liver macrophages (Kupffer cells) regulate metabolism and synthesis of hepatocytes. Although ischemia activates Kupffer cells to produce and secrete cytokines, a potential correlation between ischemia and cytokine release into blood remains to be determined. Moreover, as a result of veno-venous bypass during liver transplantation, the effect of gut ischemia with bacterial translocation and endotoxemia on cytokine release is unclear. It was the objective of this study to investigate, whether duration of liver ischemia correlates with cytokine and endotoxin serum levels in the early post operative course. Forty patients undergoing liver transplantation were studied. Arterial blood samples were taken before laparotomy, at the beginning of the anhepatic phase, 10 minutes before reperfusion,5,15,30,60,90,120 and 240 minutes after reperfusion. TNF- and IL-6 serum levels were determined using bioassays (WEHT 164 cytotoxicity assay (TNF); 7TDI-proliferations assay (IL-6)), and endotoxin plasma levels with a turbidimetric limulus amoebocyte assay. The duration of ischemia varied from 270 up to 1050 min with a mean of 566±20% min. Peaks for circulating TNF were observed 5 min(18.7±10.5 U/ml) and 30 min (20.0±17.7 U/ml) after reperfusion compared with 8.1±2.4 U/ml before operation and 4.5±1.9 U/ml 4 hrs after surgery. Serum levels of circulating IL-6 raised from 54.6±27.3 U/ml before surgery to 842.0±177 U/ml during reperfusion (5-120 minutes) and decreased to 376.8±217.8 U/ml 4 hrs after surgery. Increased endotoxin levels (with a range from 6-150 pg/ml) were detected in 23 patients. Duration of ischemia did not correlate with TNF(r=0.15), IL-6 (r=0.05), and endotoxin serum levels (r=0.15) using the Bravais-Pearson regression analysis. Moreover, no correlation was detected between serum levels of TNF-, IL-6 and endotoxin. Orthotopic liver transplantation resulted in detectable cytokine and endotoxin levels during reperfusion. Cytokine levels did neither correlate with endotoxin levels nor with the ischemic time of the transplant. Increased cytokine serum levels may predominantly be due to activation of Kupffer cells. Duration of liver ischemia does not seem to represent a major factor for induction of the inflammatory response by residual liver macrophages.

FP117 EXPERIENCE WITH COMBINED LIVER AND PANCREAS-FOR-ISLETS PROCUREMENT.

V.Mazzaferro,E.Regalia,C.Socci,
G.Colella,V.Sansalone,F.Romani,
G.Rossi,M.Colledan,F.Bertuzzi,
V.Di Carlo,G.Pozza and L.Gennari
Istituto Naz.Tumori,Ist.Sci.S.Raffaele,
H.Niguarda,H.Policlinico Milan-Italy

Transplantation of the liver and pancreas are accepted clinical procedures; nevertheless some questions regarding the combined procurement of both organs from the same donor are still pending. During 1992, with respect to islet preparation 40 pancreas were procured in combination with the liver. At the time of the procurement 3 main surgical steps were followed:
1) Arterial(aortic) perfusion of < 2000 ml of UW-Solution (i.e. avoid pancreas overperfusion),2) Decompression of portal system after the first 200 ml of arterial perfusion (i.e. early vent of the splenic vein), 3) Injection of collagenase in the pancreatic duct within 45' from the aortic cross-clamp.

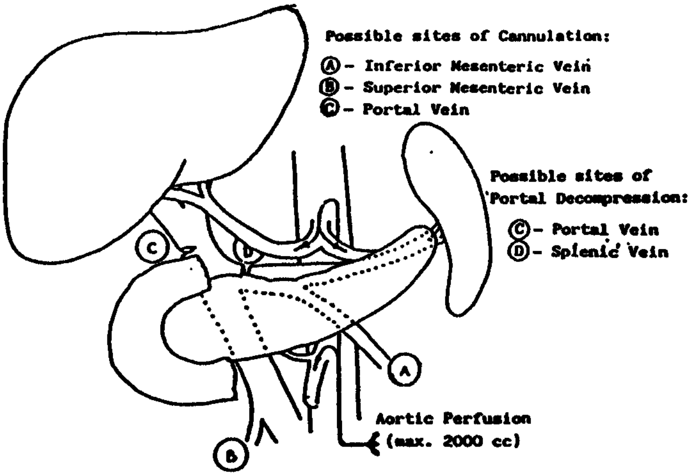

Islet function was tested by in vitro perfusion of two samples of 150 islet/equivalent from each preparation. 209.473 ± 179.250 purified islets were separated from 69.64 ± 19.95 gr. of pancreas. Insulin release in response to glucose increased significantly (Δ - AVC = 81.31 ± 18.49 g/islet/min ) indicating the functional integrity of the isolated islets.
A primary non-function of the liver graft was observed only in 2 out of 37 cases (5.4%).
Reproducible surgical recommendations allow good quality liver and pancreas grafts.

FP118 **ISLET ALLOTRANSPLANTATION IN COMBINATION WITH LIVER REPLACEMENT FOR METASTATIC NEUROENDOCRINE PANCREATIC TUMOR.**

C Socci, V Mazzaferro, E Regalia, F Bertuzzi, S Andreola, G Colella, P Magistretti, F Gavazzi, L Piemonti,P De Nittis, Doci R, Bozzetti F, Di Carlo V, Pozza G and Gennari L.
Istituto Scientifico San Raffaele, Università degli Studi di Milano; Liver Transplant Unit, Ist. Nazionale dei Tumori, Milano, Italy.

Islet transplantation is a new procedure for the replacement of pancreatic endocrine secretion in type I diabetic patient. In this study a patient with a carcinoid of the pancreas and multiple metastasisis of the liver underwent to medical and surgical treatment. After 7 cicles of chemotherapy (5FU + DTIC + EPIADH) the tail, the body of the pancreas and the spleen were removed. Further 4 cicles of chemotherapy were performed during the following six months. Then the liver and the head of the pancreas were removed with a subsequent liver - islet allograft. The patient received 485,000 (Absolute Number) fresh islets from three pancreases plus 700,000 cryopreserved islets from one gland. The islets were separated using a modification of the automated method for isolation of human islets and purificated by centrifugation on EuroFicoll gradients. All the organs were compatible with the recipient for blood group without regarding HLA matching. The islets were injected in the portal vein immediatly after the liver revascularization. Serum C- peptide increased from < 0.15 to 3.92 ng/ml but the liver function quickly worsened (AST 6390, ALT 5040, PT < 15%). Two days later, another liver transplantation had to be performed. The organ came from a 26 years old donor. The pancreas allowed to obtain 190,000 fresh islets. The day after another gland compatible with the recipient was processed and 620,000 islets were separated. The islets were injected in the portal vein by a percutaneous trans - hepatic approach. Immunosuppression consisted in cyclosporine and prednisone. Due to a transitory renal failure cyclosporine had to be stopped between the 5th and the 9th days p.o. During this period a short course of anti - lymphocyte serum was administrated. However no significant decrease in the blood lymphocytes count occurred. Four days after the transplantation, a biopsy of the liver allowed to identify two islets well shaped with ß - cells.
Three weeks after the transplant the liver and the islet grafts showed a good function (AST 30, ALT 79, PT 54%, Serum C-Peptide between 3 and 6.95 ng/ml). Insulin requirement is about 50 units/day but with 20 mg of prednisone for immunosuppressive purpose and a parenteral administration of 1250 calories in glucose. No side effects related to the percutaneous islet transplant occurred.
In conclusion, the high levels of serum C-peptide indicate the integrity of islet graft making islet transplantation an alternative to pancreas transplant in total pancreasectomized patients.

FP119

### THE INFLUENCE OF COLD ISCHEMIA TIME ON BILIARY COMPLICATIONS FOLLOWING LIVER TRANSPLANTATION

**B Dousset, M Scotte, Y Calmus, F Conti, D Houssin, Y Chapuis.**

Clinique Chirurgicale, Hôpital Cochin, Paris, France.

The aim of this study was to determine whether cold ischemia time and preservation injury judged on early liver function tests were acting upon the incidence and the type of biliary complications (BC) following orthotopic liver transplantation (OLT).

**Patients and methods:** 92 adult patients, who received 100 full-size liver allografts between 87 and 91 were analyzed. All grafts were ABO-compatible and preserved in UW solution. 18 patients (24 grafts) died within 7 days following OLT or urgent retransplantation without BC, leaving 74 patients (76 grafts) eligible for statistical analysis. The methods used for biliary reconstruction were end-to-end choledochocholedochostomy (n=26) over a T-tube in most cases (19/26) and Roux-en-Y choledochojejunostomy (n=50). Mean cold ischemia time was 10.2 +/- 0.5 hours (range 3.6-19). The numbers of graft preserved beyond 13 and 15 hours were 21 and 14, respectively.

**Results:** 18 patients (19.6%) developed 25 biliary complications: 8 anastomotic leakages (AL), 8 anastomotic strictures (AS), 6 non-anastomotic strictures (NAS), 2 cystic duct mucoceles and 1 biliary fistula following T-tube removal. Despite a high rate of reoperative surgery (70%), there was no death related to BC. Cold ischemia time was not significantly shorter in patients without BC (10.3+/-0.6 h) than in those with BC (10.1+/-1.1h) , biliary strictures only (10.5+/-1.3 h) or NAS only (12.0+/-1.8 h). Biochemical parameters of early graft function including highest value of AST, serum bilirubin and prothrombin rate during the first 3 days did not significantly differ in these 4 groups of patients. An univariate analysis of several features including initial liver disease, age, sex, pre-transplant steroid therapy, previous surgery, volume of blood transfusion, type of biliary reconstruction, acute rejection and CMV infection failed to identify predisposing factors for BC or strictures. Despite a small number of patients, chronic rejection appears to be the only significant risk factor for NAS (p=0.05).

**Conclusions:** (1) Prolonged cold ischemia time up to 19 hours is not affecting the rate or the type of biliary complications after OLT. (2) In view of these data, there is no warrant for reconsidering prolonged cold ischemia up to 15 hours in UW solution, as it has transformed liver transplantation from an emergency operation to a semi-elective procedure and allows longer back-table preparation for graft reduction or splitting.

FP120

### CHOLANGIOCELLULAR CARCINOMA - RESECTION AND TRANSPLANTATION

**P.Lamesch, B.Ringe, A.Weimann, J.Klempnauer, R.Pichlmayr**

Medizinische Hochschule Hannover

Klinik für Abdominal- und Transplantationschirurgie

Konstanty - Gutschow - Str.8

3000 Hannover 61 - FRG

### INTRODUCTION

Cholangiocellular carcinoma(CCC) can be defined as intrahepatic cholangiocarcinoma and must be distinguished from hilar or proximal bile duct carcinoma. In comparison with hepatocellular carcinoma (HCC), CCC represents the second most common primary hepatic malignancy accounting for 10% of all primary liver cancer. The rarity of this malignancy becomes evident in the relative small number of experiences that can be drawn from the literature. In the present retrospective study 40 consecutive patients treated for CCC by resection and transplantation were reviewed.

### PATIENTS AND METHODS

40 patients (15 m, 25 f, age 32-81 years, mean 52 years) were treated for CCC between 6/1979 and 12/91. According to UICC, pathologic tumor classification and stage grouping was applied. 23 patients (group 1) underwent partial liver resection (LR), 17 patients (group 2) were treated by removal of the liver and subsequent transplantation (LTX). The postoperative course was analysed with regard to survival and tumor stage.

### RESULTS

In group 1 (LR) 57% of the patients had an advanced tumor stage III, IV (I n=1; II n=9; III n=6; IV A n=7; IV B n=0) compared to group 2 (LTX) 82% (I n=0; II n=2; III n=2; IV A n=11, IV B n=2). The mean survival after hepatic resection was 15 months compared to 5 months following transplantation (p<0,01). After transplantation all patients died, the majority within 1 year after LTX of tumor recurrence, the longest survival was 25 months after transplantation. Actually there are 4 resected patients surviving between 27 and 46 months without tumor.

### CONCLUSIONS

These results show that prognosis of CCC is more unfavorable as compared to HCC and proximal bile duct cancer. Whereas curative partial resection allows prolonged survival, hepatic transplantation from our experience does not seem justified.

FP121

### LIVER TRANSPLANTATION WITH COMBINED ADJUVANT TREATMENT IN HEPATOCELLULAR CARCINOMA

D Cherqui, P Piedbois, D Vavasseur,JY Pierga, C Duvoux,
F Charlotte, D Mathieu, M Julien, D Dhumeaux, PL Fagniez

Hôpital Henri Mondor 94010 Créteil, France

The results of liver transplantation in the treatment of hepatocellular carcinoma (HCC) have been poor because of a high rate of local and distant recurrences with survival rates below 30% in most series. In order to reduce the risk of relapse, we initiated a protocol of adjuvant treatment including preoperative hepatic arterial chemoembolization (HACE), radiotherapy (RT) and postoperative chemotherapy (CT).

**Methods** : HACE consisted of injection of adriamycin, spongel and lipiodol in the hepatic artery during angiography. It was performed after inclusion of the patient in the protocol and repeated if the transplantation was not performed within 2 months. RT was performed in the immediate preoperative period at a dose of 5 Gy in one fraction on the liver volume. CT consisted of weekly doses of mitoxanthrone (5mg/m2) for 4 months, starting on postoperative day 1 if the patient condition allowed it.

**Results** : From 1989 to 1992, 10 patients (9 men, 1 woman, 27-56 year-old) were included in this study. HCC was developed in all cases in a cirrhotic liver. The tumor was solitary in 4 cases (4-9 cm) and multifocal in 6 cases (2-9 nodules, 2-7 cm). Eight patients had 1 to 4 courses of HACE (mean 2), and 2 did not have it because of portal vein thrombosis in one case and reversed portal flow in the other. One patient with Child's class C cirrhosis and HCC died of liver failure after HACE. Seven patients received RT while it was not done in 2 for technical reasons. Standard liver transplantation was performed in 9 patients. Ciclosporine, steroids and azathioprine were given for immunosuppression. One patient died at day 5 of heart failure without receiving CT. The other 8 patients survived and received CT (4-12 courses, mean 9). The first dose was administered at day 1 in 6 cases and delayed to day 8 and 15 in 2 cases because of renal failure and pulmonary edema respectively. Morbidity included mainly hematologic toxicity of CT with leucopenia which led to suppress azathioprine from the immunosuppression regimen in the last patients. Two patients relapsed and died 7 and 11 months after transplantation, and 6 (60%) are alive and free of disease (mean follow-up 17 months, 6-34 months).

**Conclusions** : These results show the feasibility of an aggressive adjuvant therapy in patients transplanted for HCC and suggest a possible effect of such a protocol in the prevention of tumor recurrence.

FP122

### RESECTION VERSUS TRANSPLANTATION FOR HEPATOCELLULAR CARCINOMA ASSOCIATED WITH LIVER DISEASE

B Launois, F Siriser, E Bardaxoglou, B Chareton, B Meunier G G Jamieson, M Messner

Department of Digestive Surgery and Transplantaion Unit, Pontchaillou Hospital, Rennes-France

Management of hepatocellular carcinoma associated with liver cirrhosis remains controversial. Liver transplantation appears particularly indicated because it allows treatment of the liver malignancy as well as the underlying liver condition. Long term results are however poor. Thus, liver resection is considered by many to be the treatment of choice, even though recurrences in the remaining liver parenchyma are frequent. The aim of this study was to compare these treatment modalities.

Between April 1978 and February 1992, 74 patients underwent surgery for hepatocellular carcinoma associated with liver cirrhosis (70 males, 4 females, mean age : 60 ± 9 years, range 31-80 years). Fifty-seven patients were rated Child A (77%) and 17 were rated Child B (23%). The etiology of cirrhosis was alcoholic (58%), hepatitic (30%) and hemochromatosis (12%). Tumour staging was the following: T1, 6.7 %, T2, 39.2 %, T3, 44.6 %, T4, 9.5 %. Sixty patients underwent liver resections and 14 patients underwent liver transplantation. Total operative mortality was 15 %; 10 % following liver resection and 35.7 % following liver transplantation ($p < 0.05$). Factors that were found not to influence survival were: tumour size and number, presence of a tumour capsule, vascular spread, staging and differentiation of tumour, and Child rating. Actuarial survival at 3 years (operative mortality included) was 38.6 % following liver resection and 27% following liver transplantation (N.S.).

Liver resection attained similar results to those of liver transplantation when 3 year actuarial survival and tumour recurrence was considered. Thus, we believe that liver transplantation should be considered only when liver resection is contra-indicated.

FP123

### MANAGEMENT AND PROGNOSIS IN PROXIMAL CHOLANGIOCARCINOMA

A Principe, R Golfieri, ML Lugaresi, F Ruberto, A Colangelo, A Frena, G Gozzetti

2° Dpt. of Surgery, Univ. of Bologna, S. Orsola Hospital, Bologna, ITALY

Proximal cholangiocarcinoma is a rare lethal disease and associate with a poor prognosis. They are often invasive of the intraepatic bile ducts along the wall and so we have been performing preoperatively percutaneous transhepatic cholangiography to this patients for the diagnosis of this neoplastic invasion. A review of our experience revealed the only those patients undergoing resection had an opportunity for long survival. **PATIENTS AND METHODS** During on eleven years period 43 patients were assessed for treatment of hilar cholangiocarcinoma. The patients selection for a correct management stems from an accurate preoperative work-up. In all patients, percutaneous transhepatic cholangial drenaige was done. Operative ultrasonography aids the decision making and engineers radical surgery. We classified tumours according to Bismuth classification. **RESULTS** Two patients died before the operation. Radical surgery was carried out in 11 patients (27 %). In type I and II the tumor with the bile duct bifurcation was resected in 3 cases (in 1 case along with a W-R) and an hepatectomy was performed in the others 8 patients (type III). There was no operative mortality. Twenty-one patients (51 %) were diemed irresectable and the treatment was palliative: 9 bilio-digestive anastomoses, 7 internal biliary drainage and 5 external biliary drainage. Two patients died in postoperative period. The mean survival rate after radical surgery in 7 patients being 56.4 months (range 13-130 months), 9.6 months after palliation and 4.1 months after explorative laparotomy only. **CONCLUSIONS** An improved prognosis can only be achieved with early diagnosis and radical surgical resection.

FP124

### RADICAL RESECTION FOR AMPULLARY CARCINOMA. LONG-TERM RESULTS.

C Sperti, C Pasquali, # A Piccoli,

* C Sernagiotto and S Pedrazzoli.

Semeiotica Chirurgica, # Medicina Interna, University of Padua, * Surgery, C.H. Montebelluna, Italy

Cancers arising from the region of the ampulla of Vater have a better prognosis than other tumours originating from the peri- ampullary area. Several factors are known to affect survival after radical resection for ampullary carcinoma, but conflicting results have been reported. We reviewed our experience with ampullary carcinoma in order to identify pathological factors influencing survival and long-term follow-up after resection. From 1970 to 1990, 30 patients underwent potentially curative resection for adenocarcinoma of ampulla of Vater (25 duodenopancreatectomy, 1 total pancreatectomy and 4 local excisions). According to UICC staging system 19 patients were in stage I-II and 11 in stage III-IV. Tumour size ranged from 1.1 to 5.2 cm; 7 patients had lymph-node metastases, 9 had pancreatic invasion. The tumour was well differentiated in 19 cases, moderately-poorly differentiated in 11 cases. One patient died in the peri-operative period (3%). Overall 5-year actuarial survival rate was 37%, 45% in the absence of lymph- node involvement versus 0% of patients with nodal metastases (p=0.03). Estimated 5-year survival rate was 51% for well-differentiated tumours (p=0.0001) versus 0% for moderately-poorly differentiated tumours.Survival was significantly better (p=0.0002) in patients with tumour stage I-II than in III-IV. Tumour size, pancreas invasion and jaundice, did not correlate with survival. After local excision, 2 patients developed local recurrence after 6 months and 3 years respectively. Among the patients (15) without nodal metastases and treated with duodenopancreatectomy, local and hepatic recurrence were found after 3,3,4,5,9 years respectively.

In conclusion, long-term survival may be obtained by radical resection in patients with ampullary carcinoma, but late tumour recurrence requires a long and careful follow-up. Lymph-node involvement, tumour differentiation and stage, are the most important factors influencing long-term survival.

FP125

### GASTRIC EMPTYING AND GASTROINTESTINAL MOTILITY ARE DISTURBED IN PANCREATIC AND PERIAMPULLARY CANCER PATIENTS WITH BILIARY OBSTRUCTION.

W.A.J.J.M. Haagh, L.M.A. Akkermans,
K.J. van Erpecum, H. Obertop.

Depts. of Surgery and Gastroenterology, University
Hospital Utrecht, Utrecht, The Netherlands.

Pancreatic and periampullary (PP) cancer patients can present with jaundice, anorexia and symptoms of indigestion. These symptoms may be related to disturbances in gastrointestinal (GI) function, in conjunction with the presence of biliary obstruction. The aim of this study was to assess GI function in PP cancer patients in relation to the degree of biliary obstruction.

We studied gastric emptying (GE) of a liquid nutrient-rich meal in 28 preoperative PP cancer patients using Applied Potential Tomography, a technique measuring changes in resistivity in a thick cross-section through the abdomen at the level of the stomach. All patients had undergone endoscopic biliary drainage before the study with variable response as assesed by the serum bilirubin level. No duodenal obstruction was present in any of the patients as evidenced by endoscopy. Normal values of GE were determined with the same method in 25 sex and age-matched healthy controls. $T\frac{1}{2}$ in these controls was 109 $\pm$ 34 min (mean $\pm$ SD). Delayed gastric emptying was defined as $t\frac{1}{2} > 180$ min (mean + 2 SD). Also six channel 24-hour ambulatory antroduodenal manometry was carried out in obstructive jaundice patients before biliary drainage to assess interdigestive GI motility (n=4).

Gastric emptying was delayed in 8 PP cancer patients (29 %), two of whom had external biliary drainage. The bilirubin level was 70 $\pm$ 90 $\mu$mol/l (mean $\pm$ SD) in these patients versus 20 $\pm$ 20 $\mu$mol/l in patients with a normal gastric emptying rate ($p < 0.05$). There was a significant correlation between gastric emptying and a bilirubin level above normal (>17 $\mu$mol/l) ($r = 0.5273$; $p = 0.043$). Ambulatory antroduodenal manometry showed gross disturbances of normal interdigestive motility in all patients. There was predominant irregular contractile activity with some clusters of contractions. Phase III of the migrating motor complex was absent or occured very infrequently and was not propagated.

Conclusion: Biliary obstruction has adverse effects on both GE and interdigestive GI motility. These effects may be related to diminution or absence of intraluminal bile in the GI tract.

FP126

### PROGNOSTIC SIGNIFICANCE OF TUMOR DNA CONTENT IN CARCINOMA OF THE HEPATIC DUCT CONFLUENCE

Y Sato, TM van Gulik, A Bosma, NJ Lygidakis,
K.Koyama, MN van der Heyde.
Dept.of Surgery and Pathology, Academic Medical
Center, Amsterdam, The Netherlands.

**Introduction.** In an attempt to identify prognostic factors in carcinoma of the hepatic duct confluence, the value of tumor DNA content was studied in 58 cases of this type of malignancy.

**Methods.** Of 58 patients (age 26-74 years) surgically treated for carcinoma of the hepatic duct confluence, tumor DNA content was assessed in relation with clinical-pathological characteristics and patient survival. The resected specimens were analyzed for nuclear DNA content of the tumor by flow cytometry. 33 patients underwent additive radiotherapy.

**Results.** Resection was radical in only 3 patients with negative surgical resection margins, as well as dissection (cleavage) margins. 28 patients (48%) had diploid tumors and 30 patients (52%) aneuploid tumors. No significant correlation was found between tumor DNA ploidy, degree of tumor differentiation, lymph node status, and hepatic infiltration. Aneuploid tumors were significantly associated with neural invasion. The median overall survival was 18 months. Survival of patients with a diploid tumor was significantly ($p<0.0003$) longer than those with aneuploid tumors (median survival 26 months and 11 months, resp.). Additive radiotherapy improved survival significantly only in patients with aneuploid tumors. When tested by univariate survival analysis, DNA ploidy, additive radiotherapy and the state of the surgical resection margins were significant prognostic factors. With multivariate survival analysis, only DNA ploidy, age, hepatic infltration and lymph node status were significantly related to prognosis.

**Conclusion.** This survival analysis shows that DNA ploidy is a powerful prognostic determinant of carcinoma of the hepatic duct confluence.

FP127

### LONG TERM RESULTS OF THE MUCOSAL GRAFT OPERATION FOR BENIGN BILE DUCT STRICTURES.

T.Kakavoulis, A.E.Ziros

A'Surgical Unit, "Agia Olga"
General District Hospital,
Athens, Greece.

From 1979 through 1992, fifteen patients aged 40 to 70 years old, with benign strictures of the axtrahepatic ducts were treated. Percutaneous transhepatic cholangiography was optimal preoperative diagnostic procedure to define the site of strictures. In 11 patients the stricture was iatrogenic and in 4 was due to abdominal trauma. Mucosal graft technique with two months stending was performed in every patient. All of them had undergone one or more attempts at stricture repair before referral to us. The mean follow up has been 8 years [5 to 11 years]. Further surgery because of anastomotic stricture has been necessary in one patient. Four patients presented recurrence of stricture 10 years postoperatively. Two of them underwent a hepaticojejunostomy with a cutaneous access stoma in the Roux en Y jejunal loop of anastomosis. The other two underwent a hepaticojejuno- stomy with the end of the extended limp of the Roux en Y loop fixed to the abdominal wall with a wire stitch around it for later radiological intervention. According to our late results the mucosal graft repair is an effective procedure in patients who have had previously unsuccessful bile duct stricture repair, but most of them after a period of 10 years may develop stricture of the anastomosis.

FP128

### SURGICAL TREATMENT OF INTRAHEPATIC BILIARY STONE AND STRICTURE

Yang Sen-hua, Yang Yi-cun

Department of Surgery, Chengdu Third People's Hospital, Chengdu, Sichuan, China

Intrahepatic stone (IHS) and intrahepatic duct stricture (IHDS) are common in China. Due to prolonged biliary obstruction and repeated infection, IHS and IHDS might result in liver and biliary lesions widely. Their treatment might be very difficult and there are various kinds of operations for managing them. Lately, in this hospital, elective operations for 63 cases were as follows. In 19 cases of choledocotomy and drainage with T tube stones were retained in 16 (84.21% ). Bilio-enteric drainage in 29 cases without treating the co-existing lesions only gave fair results in 8 cases, and this procedure, if abusively adopted, might result in more trouble such as reflux cholangitis. 17 cases (58.62%), developed mild or severe recurrences and 2 deaths resulted from intrahepatic infection. Typical hepatic lobectomy alone was rarely indicated, 3 cases had been operated on. 11 cases were treated with combined operations including thoroughly removing stones, properly relieving the IHDS, resecting the severely damaged liver and establishing synthetically the external and the internal biliary drainage. Post operatively 10 (90.91%) remained fair for 4.88 years on average. Above results seem to suggest in treating hepatolithiasis, the liver and biliary system should be considered as a whole, for delayed patients the operation should be performed directly against various lesions.

FP129 BENIGN STRICTURES OF THE LOBAR HEPATIC DUCTS AND THE REGION OF THEIR JUNCTION

E I Galperin, N F Kuzovlev, K M Khubiev.

I.M. Sechenov Medical Academy, Moscow, Russia

The report presents the experience of 230 operations performed in patients with cicatricial stenosing of the lobar hepatic ducts and the region of their junction. (Type 3,4,5 after H.Bismuth) from 1973 through 1991, 75% of patients having a restricture.

When restoring the passage of bile from the liver into the intestine two basic operative techniques were used: 170 patients (Group 1) the biliodigestive anastomosis was created using a O-ring transhepatic drain which was left in place for a period of no less than 2 years; in 60 patients (Group 2) no drain was used at all. The post-operative complication rate in Group 1 was 11.7%, in the majority of cases this being due to the use of a transhepatic drain (hemobilia, collection of bile in the subphrenic space, subphrenic abscess), that in Group 2 was 6.7%. Of 170 Group 1 patients 11(6.4%) died. The main cause of death was endotoxemia due to unresolved suppurative cholangitis and liver cholangitic abscesses. There were no fatal outcomes among Group 1 patients.

Long-term results were evaluated in 219 patients followed up from 1 to 17 years. The O-ring transhepatic drain was removed in 136 Group 1 patients. Among 118 Group 1 patients in whom no less than 2 years had elapsed after removal of their transhepatic drain the recurrence of hepatic duct strictures occurred in 3 patients (2.5%) and in group 2 only in 1 patient (1.7%).

In our view, both techniques should not be opposed one against the other. Each has its own indications for use and if there is no avoiding the incorporation of the scarred tissues into the biliodigestive anastomosis it is expedient to use the transhepatic drain.

FP130 CONGENITAL DILATATION OF COMMON BILE DUCT IN ADULTS

A MULTICENTRIC STUDY OF 41 CASES.

LENRIOT J.P., OTTE J.B., ADLOFF M., A.U.R.C.

178, rue des Renouillers - 92700 COLOMBES FRANCE

Congenital Dilatation of Common Bile Duct (CDCBD) in adults are rare in European countries and few cases have been reported. The purposes of this multicentric study were to assess the frequency of abnormal pancreaticobiliary junction, evaluate the risk of cholangiocarcinoma (CC) associated with CDCBD, and analyse the results of surgical treatment.

Patients and methods. From 1975 to 1990, 41 patients with CDCBD, 32 women and 9 men were operated in 17 Centers members of AURC or ARC ; their mean age was 36,6 +/- 19 years. Patients with Caroli's disease were excluded. Jaundice with (n=16) or without (n=6) cholangitis, abdominal pain (n=13), and acute pancreatitis (n=3) were the main presenting symptoms. Six patients (15 %) had undergone previous drainage procedures. According to Todani's classification, there were 14 type I, 3 type II, 1 type III, 22 type IV A and 1 type IV B CDCBD.

Results. Anomalies of pancreaticobiliary junction were classified according to Kimura. Of the 36 patients with type I or IV A CDCBD, 29 were corectly opacified : 27 had the C-P type and 2 had the P-C type. Of the 3 patients with type II CDCBD, 2 had the P-C type. Five patients (12 %) experienced CC associated with CDCBD : one CC of the gallbladder, 3 intra-cystic CC, and 1 CC arising from ductal bifurcation. Thirty two patients underwent cystic excision with hepaticojejunostomy and cholecystectomy, associated with Whipple resection (n=2), or left hepatic lobectomy (n=2). Four patients had internal drainage. Two patients with type II CDCBD had elective cyst resection and one with type III CDCBD had transduodenal sphincteroplasty. There were one palliative biliary drainage for unresectable CC and one hepatic transplantation for CC of the ductal bifurcation.
Postoperatively, one patient died (2,4 %) and 7 (16 %) experienced abdominal complications. None was reoperated. There were 6 later deaths, from unrelated disease (n=1), and from recurrences of CC, 3 to 24 months after surgery (n=5). After cyst excision with hepaticojejunostomy 23/27 patients (85 %) followed more than 2 years have had excellent results ; 4 experienced recurrent abdominal pain or cholangitis and 2 (type IV A) have been reoperated for intra-hepatic lithiasis. After internal drainage, poor results occured in 3/4 patientes with one death (unrelated disease) and 2 reoperations.

Conclusions. 1) Anomalies of pancreaticobiliary junction are very frequent in patients with type I or IV A CDCBD. 2) Incidence of carcinoma associated with CDCBD seems to be similar in European and in Eastern countries. 3) Cyst excision with hepaticojejunostomy is the best treatment for adults but does not eliminate risk of intra-hepatic lithiasis.

FP131

### DYNAMIC GALLBLADDER SCINTIGRAPHY IN ACALCULOUS BILIARY PAIN : CHRONIC CHOLECYSTITIS VS CHOLESTEROLOSIS

W Kmiot, I Donovan, R Wolverson, M Lee, E Perry, K Harding, J Neoptolemos

Depts of Surgery & Nuclear Medicine, Dudley Road Hospital, Birmingham, UK

The management of patients with chronic acalculous biliary pain is difficult. One of the promising approaches is the use of computerised dynamic chole-scintigraphy following gallbladder stimulation with cholecystokinin (CCK-PZ).

We evaluated 55 patients with 'acalculous biliary pain' whose median (range) symptom duration was 24 (12-120) months. The patients were assessed by a Visick score (1=no symptoms; 2=mild pain; 3=moderate pain; 4=severe pain) and were followed up 3-6 monthly for a median (range) of 24 (12-60) months. The patients were divided into three groups according to the gallbladder ejection fraction (EF): low EF (<35%), N=29; normal EF (35-50%), N=10; high EF (>50%), N=16. 35 patients with Visick scores of 3 or 4 underwent cholecystectomy because of persisting symptoms; 20 did not have surgery because of symptomatic improvement (N=4) or an alternative diagnosis (N=16). 22 cases with a low EF had surgery, of whom 21 (96%) improved with Visick scores of 1 or 2 (p<0.01) compared to only 4 out of 9 (44%) with a high EF (NS). All 4 patients with a normal EF improved after cholecystectomy and all had EF values of <39%. Histology, which was available in 32 cases, revealed chronic cholecystitis in 32 (100%) and cholesterolosis in 20 (63%) of whom 10 also had microlithiasis. Only 7 out of 12 (58%) patients with chronic cholecystitis alone improved after cholecystectomy compared to in 19 out of 20 (95%) patients with cholesterolosis (p=0.03). ERCP and duodenal bile collection was performed in the first 12 patients: the ERCPs were all normal and only 7 patients were positive for bile crystals. None of the US examinations were diagnostic of cholesterolosis.

This study supports the use of dynamic CCK-stimulated gallbladder scintigraphy in patients with acalculous biliary pain, cholecystectomy being particularly indicated in those with a low EF.

FP132

### PAPILLARY ADENOCARCINOMA AND SQUAMOUS CELL CARCINOMA OF THE GALLBLADDER ARE LIKELY TO BE ASSOCIATED WITH DIFFERENT FACTORS OR CONDITIONS

F Cetta, *A Cariati, F Lombardo, M Giubbolini, A Cappelli

Institute of Surgical Clinics, University of Siena and
*University of Genoa, Italy

Fifty-one consecutive patients with gallbladder carcinoma (GBC) [11 men (M) and 40 women (W); mean age, 69.4 years] were found during the study of 2750 patients who underwent surgery for biliary tract diseases (GBC = 1.85% of all operations). Forty-eight had gallstones, which were symptomatic in 23 cases and asymptomatic in 25. In particular, 21 of these patients (4 M, 17 W, mean age 68.7) who had complete removal of the gallbladder, isolated or as a part of a more radical operation, had systematic bile (pH, culture, trypsin) and stone analysis. Data in patients with GBC were compared with clinical and laboratory findings, histologic examination, stone and bile analysis in 1000 consecutive patients with non malignant bile tract diseases, who were analyzed in the course of a prospective study. Patients with non squamous GBC (n=16) (13 adenocarcinoma (ADC), 1 papillary, 2 poorly differentiated) had cholesterol or combination stones in 14 cases, black mud in 1 case and no stone in the patient with papillary ADC. Stones were larger than 15 mm in 9 cases and smaller in 6 cases. The mean time lapse (TL) between documented lithiasis and operation was 16.3 years (n=7). Bile culture was positive in 24% of patients, pancreaticobiliary reflux (PBR) was evident in 3 of 18 patients (16.6%). Patients with squamous cell carcinoma (SQC) (n=5 patients, 1 squamous, 4 adenosquamous), always had cholesterol stones larger than 15 mm. Time lapse was 31.6 years (p=0.002). Bile culture was negative. Evident PBR was not detectable. On the basis of present data and of the literature review concerning GBC and premalignant lesions or conditions it is suggested that adenocarcinoma (ADC) (in particular papillary ADC) and SQC seem to be associated with different factors and conditions. ADC, the most frequent histologic type, is sometimes associated with PBR, but less frequently than SQC with gallstones of large size and long duration. Papillary ADC can be found in the absence of gallstones. On the contrary, SQC is more closely related to longstanding cholesterol or combination stones and to risk factors affecting their formation (female sex, parity, obesity). Instead of classifying GBC as a unique entity, it is suggested to separate carcinomas associated with or related to gallstones from those without gallstones. Distinction between these 2 groups as well as between ADC and SQC could be of importance for both epidemiologic and clinical purposes, i.e., for a proper recognition of the risk factors, a better knowledge of the natural history of the illness and a correct evaluation of the therapeutic options.

FP133

### PROSPECTIVE RANDOMIZED TRIAL OF ELECTROSURGERY VS ULTRASONICALLY ACTIVATED SCALPEL FOR LAPAROSCOPIC CHOLECYSTECTOMY

J Amaral

Department of Surgery, Rhode Island Hospital
Brown University, Providence RI U.S.A.

The ultrasonically activated scalpel (UAS) is composed of a blade that vibrates axially 55,500 x per second resulting in cutting and simultaneous coagulation. Animal studies have shown little lateral thermal damage (1mm), no smoke, no grounding needed and minimal tissue injury. The purpose of this study was to compare the UAS to conventional electrosurgery (ES) for human laparoscopic cholecystectomy. Thirty-five patients were randomized to either ES or UAS by choice of their surgeon. All patients had normal preoperative liver function tests. Preoperatively there was no difference between ES and UAS for hemoglobin, white blood cell, LDH, SGOT, SGPT, alkaline phosphatase and bilirubin. These values were obtained preop and 24 hours postop. (*=p 0.05)

| Postop | N | Hgb | WBC | Bili | Alk | LDH | SGOT | SGPT |
|---|---|---|---|---|---|---|---|---|
| UAS | 15 | 12.7 | 8.71 | 0.7 | 75.6 | 559* | 62* | 89* |
| ES | 20 | 12.2 | 8.17 | 0.7 | 68.0 | 375 | 40 | 55 |

There was no difference in length of hospital stay or operative tissue. There were more gallbladder perforations with ES (3) than UAS (0). These data are consistent with the hypothesis that there is less tissue damage with UAS thqn ES since postoperative LDH, SGOT and SGPT were significantly greater for ES than UAS. Despite the reduced tissue damage UAS was as hemostatic as ES as reflected in hemoglobin. In conclusion, UAS is as effective as ES with less tissue damage and greater safety.

FP134 **COMPARATIVE EVALUATION OF EARLY CHOLECYSTECTOMY VERSUS INTERVAL CHOLECYSTECTOMY IN ACUTE CHOLECYSTITIS**

T.K. Malik, S.Madaan, R. Malik

Deptt. of Surgery, Maulana Azad Medical College, New Delhi.

A total of 224 patients with a provisional clinical diagnosis of acute cholecystitis were taken up for study. All the patients were subjected to ultrasonographic examination and/or DISIDA scanning for the confirmation of diagnosis. Initial clinical diagnosis proved to be wrong in 11.6% (26) of patients. The remaining 198 patients were randomly allocated into early (n=98) and delayed (n=100) surgery group. Those taken up for delayed surgery were continued on conservative management till acute symptoms subsided and were discharged to be readmitted for elective surgery 3 months later. The patients undergoing early surgery were operated on within 7 days of the onset of acute symptoms under antibiotic cover. Per-operative saline biliary manometry was done in all cases to detect any unsuspected CBD stones and CBD explored whenever necessary.

In delayed surgery group 8% of patients had to be operated early due to failed conservative management and 25% of patients had to be readmitted due to recurrence of symptoms before the planned date of surgery. Incidence of CBD stones was similar in two groups (Early surgery - 8.16%; Delayed surgery - 8%). No undue technical difficulty was encountered during early surgery with operating time being similar in two groups. The morbidity and mortality was comparable in two operative groups. Total duration of hospital stay was 6.3 days fewer in the early surgery than in the delayed surgery group, thereby reducing hospital costs. On the basis of this study we recommend early cholecystectomy as a routine in the management of patients with acute cholecystitis.

FP135

### ASSESSMENT OF SECRETED MUCOPROTEINS IN THE GALL-BLADDER WITH CALCULOUS

Alvarez J, Lobato R, Carabias A, Maillo, Jover J, Lucia P, Moreno Azcoita M.

Hospital Universitario de Getafe. Madrid, Spain

Different substances like cholesterol, calcium carbonate and proteins are consolidated upon mucoproteins secreted by the gall-bladder forming a lithiasis. Mucoproteins play an essential role in lithogenesis.

OBJECTIVE: Determine pattern and amount of secretion of gallbladder mucoproteins.

PATIENTS AND METHODS: Prospective study in cholecystectomy specimens. By means of different histochemical analyses every mucoprotein was determined, and the intensity of the colour showing the amount.

RESULTS: Overall mucoprotein was 1-24 (11.6 ± 5.2). In mucous membrane was 1-9 (3.7 ± 1.8), and in glands 0-9 (4.7 ± 2.1). Neutral mucine showed a mean intensity graduation of 0-12 (4.4 ± 1.9), and acid low sulphated 0-9 (2.4 ± 2.4).

CONCLUSION: Mucoproteins are produced in secretory grains by distal pole of the cell, with a larger intensity in the glands than in epithelial cells. In cholelithiasis an unspecific secretion of mucoproteins is produced.

FP136 OPEN CHOLECYSTECTOMY IN THE '90s
STILL A COMPETITIVE OPTION

PG SFIKAKIS, SB KONTOSTOLIS, NL LEKACOS

2nd Surgical Unit, Tzanion General Hospital,
Piraeus, Greece

Open surgery remains the most prominent treatment for gallstone disease although recently, the laparoscopic option was the breakthrough which established minimal access surgery with minimum postoperative discomfort and accelerated recovery with early return to full activity or work. We have been persuaded that mini-laparotomy for cholecystectomy, has similar results with the laparoscopic one with significant cost savings.
All 81 patients admitted for elective cholecystectomy had cholelithiasis diagnosed preoperatively by ultrasound and confirmed at surgery. All had a mini subcostal incision. Dissection of the gallbladder, was from above downward. Complete hemostasis of the liver bed was achieved with a sheet of absorbable collagen hemostatic sponge (HELISTAT) in the liver bed. A closed system drainage was employed in only 6% and was removed within 24 hours. The abdominal wall was being closed in routine fashion with two layers of 2 vicryl, after the cut surfaces of peritoneum, fascia, subcutis and skin were infiltrated with 200 to 250mgr (40 to 50ml) of 0.5% bupivacaine hydrochloride (Marcaine). The skin was closed with 3/0 subcuticular continuous plain cat gut. Nasogastric decompression was used in 12% of patients and the tube was removed within the first 12 to 24 hours. Perioperative antibiotics prior and 2 doses after the operation were used. The patients were instructed intensivelly about the need for early ambulation and pulmonary toillet and 93% of them were dismissed the 3d postoperative day. No complication developed, was aggravated, or went unrecognized because of early discharge. Two subjects had superficial hematomas treated on an outpatient basis.
We conclude that concerning the financial point of view, mini-laparotomy remains the gold standard for patients undergoing elective cholecystectomy. On the other hand, concerning the patients' comfort, it remains still competitive to laparoscopic cholecystectomy. Patients can be discharged soon after major operations and such programs have demonstrated the safety of outpatient surveillance.-

FP137 CHOLEDOCHODUODENOSTOMY(CDD) IN THE MANAGEMENT OF CHOLEDOCHOLITHIASIS - LONGTERM RESULTS

A.M.Almeida, N.M.Santos, F.J.Aldeia

Surgical Unit I,University Hospital Sta Maria
Lisbon,Portugal

Reported as a more efficient approach to choledocholithiasis,as compared to T-tube drainage(1,2),CDD is regarded as a last resort measure due to fears of higher operative morbidity,cholangitis,sump syndrome and liver dysfunction.We aimed to assess the aforementioned issues analyzing prospectively our experience(1973-Dec 91).METHODS: CDD was performed in 89 F/36 M,60.2±8.7(m±sd)yrs old,26 as resurgery,for CBD stones 94(75%),papillary dysfunction 23(18%),pancreatitic nodule 8(6%).Peroperative liver bx was obtained,upon written consent in 44 pts.The follow-up schedule(>2.5 yrs in 110)included clinical interview and liver biochemistry profile every 4 months for the 1st year and once a year thereafter and USG every 1-2 yrs.ERCP was obtained in 10 symptomatic pts plus 25 others for academic purposes,upon written consent.Liver bx's were obtained,upon consent,4-9 yrs postop, in 11 pts(5 at relaparotomy for unrelated pathology,6 fine needle). Duct mucosa bx was obtained in one pt,at endoscopy.The anastomotic technical details were previously described(3).Longterm results were classified as previously defined(3),**poor** meaning the need for further invasive therapy.RESULTS:The operative mortality rate was 1.6%. The postoperative hospital stay was of 8.2±2.3(m±sd)days.The longterm results(123 survivors)were considered as **excellent** 89(72%),**good** 22(18%),**fair** 9(8%),**poor** 3(2%).Three pts died from unrelated causes and 8 others ceased the follow-up evaluation 3-5 yrs postsurgery,all of them classified as excellent or good result at the end point.A widely patent anastomosis(>2.5 cms)was documented in every patient assessed via ERCP,without duodenal or duct mucosal inflammatory changes,including those classified as poor result.Food "debris" were detected in the distal "cul-de-sac" of 4 pts,easily floating and flushed through the stoma.In none of the longterm liver bx's could we observe histological abnormalities,although definite cholestatic changes had been demonstrated on peroperative tissue exams.CONCLUSIONS:1)CDD is a highly efficient short and longterm treatment of choledocholithiasis,without the alleged long run morbidity such as bacterial or "chemical" cholangitis,sump syndrome or hepatic dysfunction,provided a technically correct and wide anastomosis(>25 mms) is accomplished,2)The longterm efficiency,in terms of retained and/ or recurrent calculi,of the recent laparoendoscopic approaches should be matched to what is achievable by a suitable and correct CDD.
REFERENCES:1)Br.J.Surg,1985;72:428, 2)Br.J.Surg,1987;74:1095, 3)Am.J.Surg,1984;147:253

FP138 SYMPTOM SCORING OF RIGHT UPPER QUADRANT PAIN

M Harper, IS Bailey, C D Johnson

University Surgical Unit, Southampton
General Hospital, Southampton, UK

Many patients complain of right upper quadrant (RVQ) pain, but only a small proportion of these will have gallstones. Ultrasonography (US)is the most accurate diagnostic test for gallstones, but to submit all patients with RUQ pain to US is wasteful of resources. We have investigated symptoms in patients referred for US for this indication, in an attempt to identify symptoms which can indicate the presence or absence of gallstones.

174 patients were asked to complete a detailed symptom questionnaire and the US findings were recorded. Questionnaires were then analysed by univariate and multivatiate analysis to identify symptoms associated with the presence or absence of stones.

Six features were identified which could predict the absence of gallstones: pain not severe; pain not relieved by vomiting; sharp pain; age<60 years; oesophageal reflux; and absence of jaundice.

If all six features were present, there is a 92% likelihood that the patient will not have gallstones. All six features were absent in 62% of those who had stones. Predictive values for stones were presence: 74%: absence:88%.

This analysis has given promising results in the search for a symptom score which will predict the absence of gallstones. With a high negative predictive value, it is possible to exclude from investigation patients who are unlikely to have stones. The positive predictive value is lower, but this is less important clinically, as US will be necessary for all patients in whom stones cannot be excluded.

